# Programmable Interface Atomic Rearrangement for Spatiotemporal Thermal Radiation Tailoring

**DOI:** 10.34133/research.1141

**Published:** 2026-03-06

**Authors:** Xinye Liao, Mingyu Luo, Zhaojian Zhang, Qi Jiang, Xin Li, Junxiang Zeng, Yule Wang, Xingpeng Jiang, Jie Nong, Jiagui Wu, Dongqing Liu, Huan Chen, Xin He, Xiaohu Wu, Qiang Li, Junbo Yang

**Affiliations:** ^1^ College of Sciences, National University of Defense Technology, Changsha 410073, China.; ^2^ Photonics Research Institute, Department of Electrical and Electronic Engineering, The Hong Kong Polytechnic University, Kowloon, Hong Kong SAR 999077, China.; ^3^ College of Advanced Interdisciplinary Studies, National University of Defense Technology, Changsha 410073, China.; ^4^ School of Physical Science and Technology, Southwest University, Chongqing 400715, China.; ^5^ Science and Technology on Advanced Ceramic Fibers and Composites Laboratory, College of Aerospace Science and Engineering, National University of Defense Technology, Changsha 410073, China.; ^6^ Thermal Science Research Center, Shandong Institute of Advanced Technology, Jinan 250100, Shandong, China.; ^7^ State Key Laboratory of Extreme Photonic and Instrumentation, College of Optical Science and Engineering, Zhejiang University, Hangzhou 310027, China.

## Abstract

The modulation of thermal radiation is essential for advanced photonic applications, offering a novel perspective for information carriers. However, current carriers are limited to a few discrete radiation states at isolated time points and cannot capture the entire dynamic process along the full temporal axis, resulting in substantial information loss. Here, we propose and experimentally validate a novel spatiotemporal modulation strategy that dynamically tailors emissivity across the entire temporal domain, where gentle and uniform modulation of atomic configurations enables the creation of entirely novel permittivity libraries. To realize flexible thermal radiation tailoring, we utilize an Ag–In_3_SbTe_2_ (IST) interface atomic rearrangement metamaterial (ARM). As a dynamic optical control platform, ARM exhibits continuous spectral modulation with remarkably high amplitudes (64.74% in the 3- to 5-μm band and 73.94% in the 8- to 14-μm band), together with rich variations across the temporal domain to realize tailored infrared emissivity and infrared information encryption. This work establishes a unified framework for continuous, atomic-scale spatiotemporal control of thermal radiation, opening new pathways for spectral modulation and photonic information regulation in the time domain.

## Introduction

The advent of Industry 4.0 is accelerating the integration of smart technologies, where information sensing and transmission play pivotal roles [1–4]. Meeting these demands requires not only the detection of increasingly subtle signals but also the development of richer information carriers to support intelligent systems. In this condition, thermal radiation serves as a compelling candidate. Unlike electronic or optical signals that typically require external energy input [5–8], thermal radiation is ubiquitous across physical processes, offering a readily accessible and energy-efficient channel for functional exploitation [9–14]. According to Planck’s law [15,16], thermal radiation at room temperature is predominantly concentrated in the mid-infrared, which is shaped by atmospheric absorption into 2 transparency windows: the mid-wave infrared (MWIR; 3 to 5 μm) and long-wave infrared (LWIR; 8 to 14 μm) [14,17,18]. Accurate spectra alignment across these bands, where emissivity plays a decisive role, is essential for information applications. Recent advances in thermal photonics, including multilayer thin films [11,13,19,20], metamaterials [10,21], and photonic crystals [22], have enabled active control over emission properties, demonstrating the feasibility of tailoring thermal radiation at will. The field is now moving beyond static or single-parameter modulation toward precise spatiotemporal control of thermal radiation [5,23,24], a critical step for encoding complex information and achieving programmable functionalities.

Storing and processing infrared information requires materials and devices capable of supporting multiple, stable emissive states, as each additional state directly increases information density [21,25]. A wide range of approaches has been explored to dynamically modulate thermal radiation, from quantum well [26,27] and graphene structures [28] with voltage-tunable emissivity to stretchable elastomers [9,18,24] that achieve spectral shifts under mechanical strain. However, these technologies remain constrained by high power demands and limited durability, which hinder their adoption in practical applications such as infrared anti-counterfeiting [29], secure encryption [25], and high-density data storage [30] in compact consumer devices. Nonvolatile phase-change materials (PCMs), such as Ge_2_Sb_2_Te_5_ (GST) [31–34] and Sb_2_S_3_ [35,36], have emerged as a compelling alternative, enabling reversible and reconfigurable control of emissivity in the mid-infrared while consuming virtually no static power. Their ability to preserve encoded states without continuous energy input offers distinct advantages for low-power, miniaturized systems [37–39]. Yet, conventional PCMs are intrinsically limited [21,25,30,37–42]: Their narrow thermal operation windows confine intermediate states to such limited temperature ranges that precise control and stabilization of phase fractions are difficult, leaving insufficient tunable points for emissivity adjustment. Moreover, their rapid phase transitions typically drive emissivity to evolve monotonically with increasing crystalline content, making it challenging to achieve complex and unpredictable time-dependent variation of thermal radiation.

Atomic manipulation, first articulated in Feynman’s seminal 1959 lecture [43], has long been regarded as a central objective in science and engineering. By enabling control of material properties at the atomic scale, it has opened up transformative opportunities across interdisciplinary frontiers—particularly in photonics, where it allows optical responses that are unattainable in naturally occurring materials [44,45]. For example, tailoring atomic composition during synthesis has produced transparent conducting oxides such as indium tin oxide (ITO) and aluminum-doped zinc oxide (AZO) [46,47], which possesses novel permittivity within targeted spectra ranges [48–51]. Beyond compositional engineering, atomic rearrangement can also be induced thermally via diffusion processes [52,53]. This approach provides a pathway to actively control material properties. Moreover, since the changes arise from gradual, atom-by-atom migration and mixing rather than abrupt, bulk transformations, it enables extremely precise tuning. Similarly, the continuity of the atomic migration process allows for ultra-high temporal resolution in control. Crucially, such dynamic and continuous modulation of optical constants can manifest as spectral tuning in photonic devices [53–57], unlocking new possibilities for programmable optical functionalities. Despite this potential, atomic rearrangement as a mechanism for dynamic photonic control remains largely unexplored.

In this study, we demonstrate an Ag–In_3_SbTe_2_ (IST) interface atomic rearrangement metamaterial (ARM), which can be classified as a category of metal-PCMs system. It exhibits 3 distinctive characteristics: (a) The permittivity of the mixed layer rapidly changes with increasing metal volume filling fraction, allowing for huge modulation of mid-infrared emissivity. (b) Atomic rearrangement occurs spontaneously and permits rate control through heating control, enabling ultra-wide temperature and duration tunability. (c) Crystalline IST (cIST) can achieve metallic-to-dielectric transition via atomic migration, thereby expanding its programmable functionalities. Moreover, we can achieve controllable rearrangement (temporal domain) in the patterned regions (spatial domain) based on laser direct writing, as the IST thickness and laser energy density are all adjustable. These functionalities endow ARM with information storage capabilities, serving as the foundational basis for high-redundancy encryption. Above all, the ARM has a giant modulation amplitude (see Table S1 for the comparison of infrared regulators), a flexible modulation method (see Table S2 for the comparison of thermal control IR regulators), and a robust encoding technique, which opens a new way to realize high-resolution and ultra-broadband spectra (detailed in Text S11) spatiotemporal tailoring.

## Results

### ARM and its performance

**Fig. 1.**
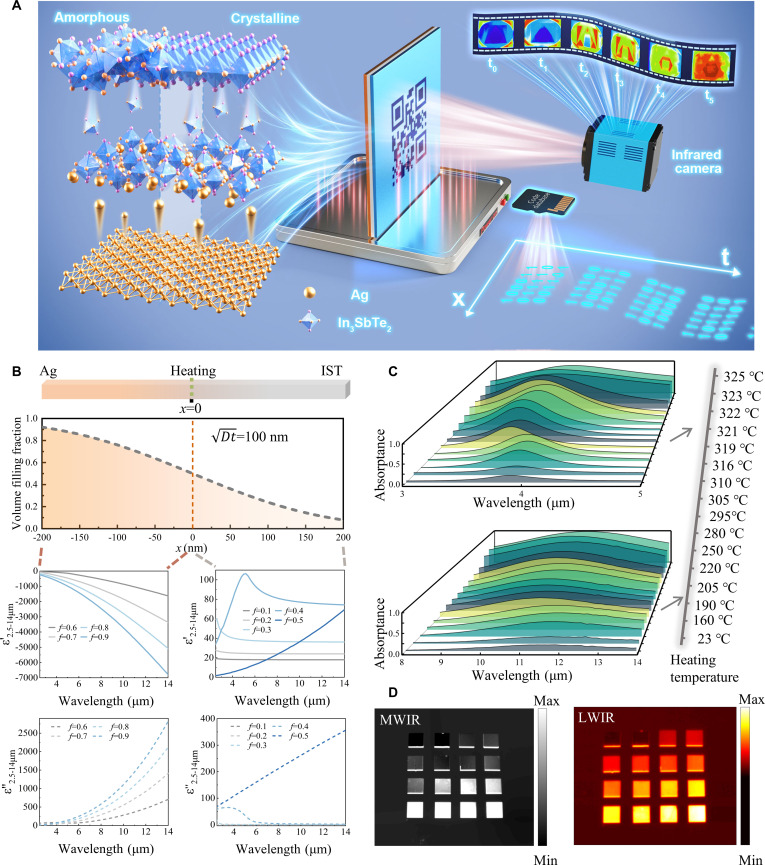
Principle and performance of ARM. (A) Schematic diagram of atomic rearrangement and the spatiotemporal encoding. (B) Ag volume filling fraction distribution across the Ag–IST interface, along with the real and imaginary parts of the permittivity. (C) Absorptance spectra. (D) MWIR and LWIR images of 16 ARM samples heated at different temperatures (Fig. S14).

Figure 1A illustrates the principle and applications of the ARM. Its temporal modulation capability arises from atomic diffusion across the IST–metal interface under thermal excitation. Both amorphous IST (aIST)–metal and cIST–metal diffusion generate a continuum of intermediate states, governed by the fractional mixing of the 2 components at different temperatures. Leveraging laser direct writing, IST can be reversibly tuned from aIST through multiple intermediate configurations to cIST, thereby greatly expanding the number of diffusion-induced optical states and corresponding emissivity levels. Furthermore, laser writing enables spatially localized phase transitions, offering precise spatial control of emissivity. On the temporal axis, the permittivity evolves gradually with temperature, allowing continuous tuning of emissivity over time and thus enabling temporal control. Demonstrated through infrared encryption, this spatiotemporal programmability facilitates ultrahigh-density thermal data storage, while the abundance of accessible emissivity levels provides substantial redundancy, importantly enhancing encryption security.

To simulate the rearrangement process, we reduced the isotropic bilayer film to a one-dimensional (1D) model. Considering a given rearrangement range, the components out of that range will obviously be unaffected. Under this boundary condition, the metal volume filling fraction *f* at a given time can be expressed as follows:

$$f=\rho \left(x,\;t\right)/\rho_{M}=\frac{1}{2}\left[1-\mathrm{erf}\left(\frac{x}{2\sqrt{\mathrm{Dt}}}\right)\right]$$ (1)

where *x* is the coordinate along the atomic migration direction, *t* is the heating time, *ρ*(*x, t*) is the metal mass concentration, *ρ*_M_ is the pure metal mass concentration, and *D* is the diffusion coefficient. The specific derivation process is detailed in Text S3.

For Ag–IST mixed layers with different volume filling fractions, the permittivity can be calculated by using effective medium theories. Considering the subwavelength conditions (Text S1), the calculation formula is as follows:

$$\varepsilon =\frac{1}{2}\left\{\left(2f_{1}-1\right)\varepsilon_{1}+\left(2f_{2}-1\right)\varepsilon_{2}\pm \sqrt{{\left[\left(2f_{1}-1\right)\varepsilon_{1}+\left(2f_{2}-1\right)\varepsilon_{2}\right]}^{2}+4\varepsilon_{1}\varepsilon_{2}}\right\}$$ (2)

where *f*_1_ is the volume filling fraction of Ag (*f*_1_ = *f*), *f*_2_ is the volume filling fraction of aIST, *ε*_1_ is the permittivity of Ag, and *ε*_2_ is the permittivity of aIST, respectively. This equation also satisfies *f*_1_ + *f*_2_ = 1, Im(ε) > 0 [judge positive and negative signs in Eq. (2)].

Figure 1B shows the spatial distribution of Ag volume filling fraction across the mixed layer under defined parameters. It presents the mixed condition in ARM after rearrangement. Next, we analyzed the permittivity of the Ag–IST across distinct rearrangement regions with different Ag volume filling fraction ($\varepsilon_{2.5-14\;\mathrm{\mu m}}^{\prime }$ and $\varepsilon_{2.5-14\;\mathrm{\mu m}}^{\prime \prime }$ for real and imaginary components). The permittivity exhibits huge variation with *f* changing. Notably, the $\varepsilon_{2.5-14\;\mathrm{\mu m}}^{\prime \prime }$ show a marked increase from *f* = 0 to *f* = 0.9, which means a large enhancement of Ag–IST’s electromagnetic loss. In Fig. 1C, we show the absorption spectra of 16 ARM samples after heating at different temperatures. ARM can achieve huge modulation in the 2 infrared atmospheric windows simultaneously, with a modulation amplitude of 64.74% (1.98% to 66.72%) in the MWIR and 73.94% (7.5% to 81.44%) in the LWIR, respectively. Additionally, by adjusting the thickness of the IST layer, single-band emissivity tuning in the MWIR was achieved (Fig. S23), where the modulation amplitude can also reach 46.7% (from 8.33% to 55.03%). Figure 1D shows the MWIR and LWIR images of the samples on the heating stage at 60 °C (detailed sample information can be found in Fig. S14). As expected, higher emissivity corresponds to brighter MWIR images and more vivid pseudo-colors in the LWIR regime. This correlation forms the foundation for implementing information storage through the modulation of infrared emissivity (see Text S5 for the power received by the infrared camera).

### Simplified simulation model and verification

**Fig. 2.**
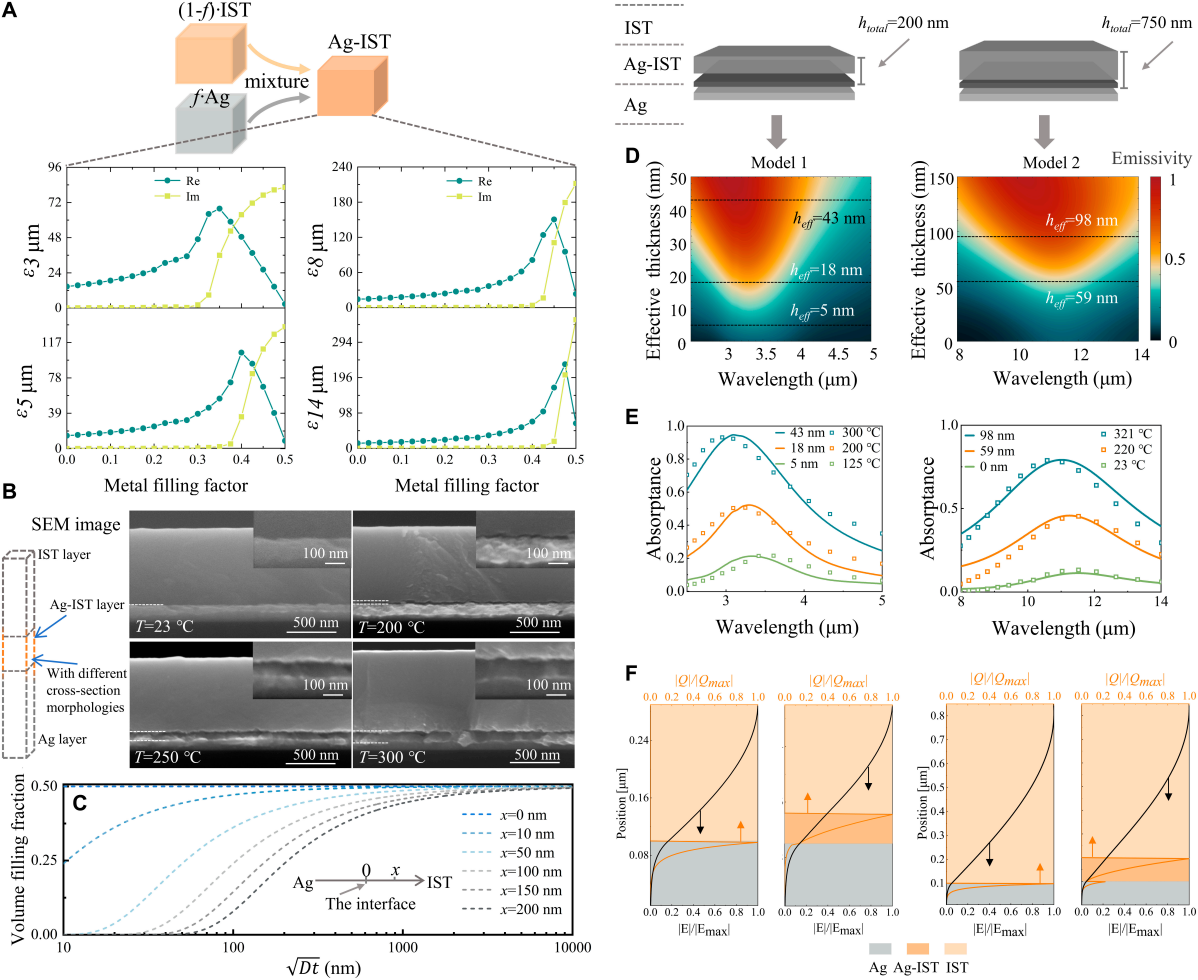
Theoretical framework and analytical verification of ARM thermal radiation modulation. (A) Equivalent permittivity of mixed materials under different Ag volume filling fractions. (B) ARM cross-sectional SEM image (model 2), heated at 23, 200, 250, and 300 °C, respectively. (C) $f-\sqrt{\mathrm{Dt}}$ curves at different diffusion distances. (D) Absorption spectra of the simulation model under different equivalent mixed layer thicknesses. (E) Comparison of experimental and simulated spectra. (F) Electric field and heat loss distribution diagram. Under model 1, the wavelength is at 3.3 μm with *h*_eff_ = 0 nm and 43 nm. Under model 2, the wavelength is at 10 μm with *h*_eff_ = 0 nm and 98 nm. In (D), (E), and (F), different bands correspond to different models: The MWIR band is model 1, and the LWIR band is model 2.

Owing to the gradient-distributed Ag volume filling fraction (*f*) in the Ag–IST layer, obtaining accurate simulation results needs a differential process (see Text S4 for the transfer matrix method in ARM). This undoubtedly increases the computational difficulty and complicates the analytical model. To simplify the permittivity model of the mixed layer in simulation, it is essential to analyze its characteristic features systematically. As shown in Fig. 2A, the imaginary parts of the permittivity are near zero for small *f* (<~0.3), whereas they increase rapidly if *f*→0.5. These suggest that optical loss will mainly occur in the high-*f* layer (i.e., close to the original interface). The real part of the permittivity initially increases and subsequently decreases. This parameter reaches its maximum at *f* exceeding 0.35 and approaches zero at frequencies above 0.5.

The structural evolution of the mixed layer is confirmed experimentally. Figure 2B shows the ARM cross-section scanning electron microscopy (SEM) images after heating at different temperatures. It reveals that as the heating temperature rises, a mixed layer emerges and its thickness increases. When the *f* in the mixed layer reaches a certain threshold, large changes in the film’s mechanical properties occur. It will lead to a distinct cross-sectional morphology compared to the upper (IST) and lower (Ag) layers [58–63].

To further analyze the relationship between volume filling fraction (*f*), the rearrangement condition ($\sqrt{\mathrm{Dt}}$), and the atomic migration distance (*x*), we calculate the curve graph shown in Fig. 2C. When *f* remains unchanged, $\sqrt{\mathrm{Dt}}$ has a large difference under distinct *x*. From this, we can conclude that the rearrangement result is highly robust to the heating temperature and duration.

Based on the above characteristics, the mixed layer can be simplified as a single effective layer with a fixed volume filling fraction (*f* = 0.5). That is, the simulation model consists of 3 layers: Ag, Ag–IST (*f* = 0.5), and IST. Further, the combined thickness of the Ag–IST layer (*f* = 0.5) and the IST layer is maintained as a constant value. As presented in Fig. 2D, increasing the thickness of the Ag–IST(*f* = 0.5) enhances the absorptance and induces a blue shift of the absorption peak. This arises from the high imaginary permittivity and low real permittivity of Ag–IST (*f* = 0.5). The extracted spectra in Fig. 2E. demonstrate strong agreement with experimental measurements.

To understand the absorptance features deeply, we calculated the electric field and heat loss distribution of ARM. In Fig. 2F, it can be observed that the electric field decreases from the top layer to the base. From the perspective of resonance, it primarily corresponds to the fundamental Fabry–Pérot (FP) mode, which consistently exhibits high intensity. In addition, other resonances have been considered, as Ag–IST exhibits epsilon-near-zero (ENZ) characteristics under specific *f* parameters. However, the ENZ layer could not enable ARM to excite the ENZ/Berreman modes [49–51,64] due to its high loss (see Text S9 for the resonance excited by high-loss ENZ materials). More interestingly, upon incorporating the Ag–IST layer into ARM, the primary heat loss shifts from the Ag/IST interface to the Ag–IST layer (*f* = 0.5). This result indicates that the atomic migration of Ag leads to increased loss. At the same time, when the real part of the permittivity is greater than 0, incident light can still couple in, thereby increasing emissivity.

### The control of atomic rearrangement and phase change by laser direct writing

Having discussed the principle of temporal spectra control via atomic rearrangement, it is equally important to explore strategies for achieving differentiated radiation behaviors in the spatial domain. In this work, we induce phase transitions in aIST through laser direct writing, thereby generating spatially patterned variations. Owing to the fact that atomic rearrangement proceeds more slowly than phase transitions, the phase state of the top IST layer and the underlying atomic rearrangement can be independently modulated, enabling versatile control over emissivity.

**Fig. 3.**
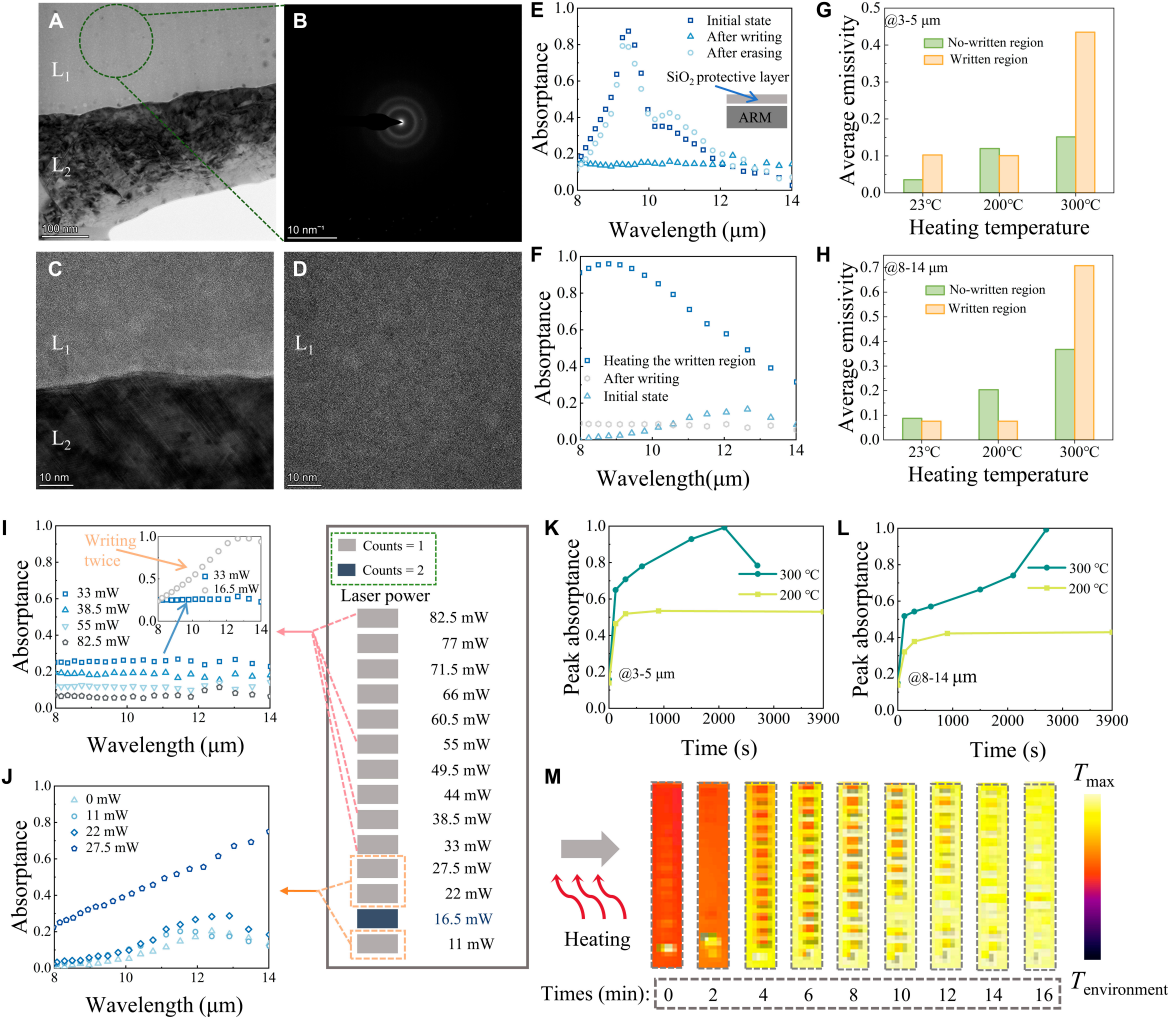
Laser direct writing application in ARM and comparison before/after direct writing. (A and B) TEM images. (C and D) High-resolution TEM images (L_1_: IST layer, L_2_: Ag layer, the sample: Ag-cIST after heating). (E) Direct writing and erasure of IST. (F) Metallic-to-dielectric transition of cIST via atomic rearrangement. (G and H) Average emissivity of written/nonwritten regions after heating at different temperatures. (I and J) Absorptance spectra of AIRM after writing at varying laser powers. (K and L) Peak absorptance variation of nonwritten ARM over time. (M) LWIR images of the sample during heating, written square: 1 mm (length) × 0.5 mm (width).

Figure 3A presents a transmission electron microscopy (TEM) micrograph of an Ag-cIST sample following heat treatment, with corresponding selected-area electron diffraction (Fig. 3B) and high-resolution TEM images (Fig. 3C and D). These results demonstrate that heating induced structural disorder, transforming the original cIST layers into an amorphous state. To investigate the associated optical property modifications resulting from this amorphization process, we measured the spectra for various samples. In Fig. 3E, we show the absorption spectra of ARM, which coats an 80-nm silica protective layer. The results demonstrate that the absorptance peak disappears after laser writing, confirming a phase change from aIST to cIST. Upon erasure, the absorptance peak reappears, confirming the reversibility from cIST to aIST. It is noteworthy that the peak of the FP resonance near 10.6 μm increases after write–erase cycles. This is because each write–erase process will induce some degree of interface atomic rearrangement, thereby enhancing the absorptance of the FP peak. Although this peak continues to rise with an increasing number of write–erase cycles, within a certain range of reconstruction cycles, the peak has little impact on the average emissivity in the 8- to 14-μm band (see Fig. S38). In Fig. 3F, we can find that the shielding effect of the cIST disappears after heating. It presents a brand-new method for the metallic-to-dielectric transition. The principle is that Ag atoms doped into cIST, destroying the original atomic arrangement (Text S10).

In Fig. 3G and H, we compare the average emissivity changes in the written/nonwritten region. The data analysis reveals that the emissivity of the nonwritten regions rises proportionally with increasing heating temperature. However, the written region’s emissivity remains unchanged until the heating temperature is high enough. That is, the metallic-to-dielectric transition in the top cIST layer occurs when the Ag volume filling fraction reaches a critical threshold during heating. Interestingly, after heating at 300 °C, the written region has a higher emissivity compared to the nonwritten region. This phenomenon is attributed to the local heating induced by laser direct writing and a higher *D* in the written region.

The crystallization fraction of the top IST layer can be tuned by adjusting laser power. As shown in Fig. 3I, increasing power (33 to 82.5 mW) leads to crystallization of aIST, eliminating the resonant absorptance peak. Lower powers reduce the crystallization fraction, reflected in higher absorptance. At fixed output power (33 mW), splitting the exposure into 2 pulses of 16.5 mW greatly increases peak absorptance (Fig. 3J), consistent with negligible crystallization and enhanced Ag–IST rearrangement. Further measurements reveal that the crystallization threshold lies between 22 and 27.5 mW, as indicated by the gradual disappearance of the absorptance peak.

We then studied spectral response and thermal imaging as a function of heating time. As shown in Fig. 3K and L, we examined the spectra evolution of nonwritten regions under prolonged heating. At a heating temperature of 200 °C, the peak absorptance no longer increases after 1,000 s, indicating that the atomic migration at this temperature has reached equilibrium. At 300 °C, ARM maintains near-perfect absorption across both the 3- to 5-μm and 8- to 14-μm bands under prolonged heating. Notably, the peak absorptance within 3 to 5 μm exhibited a decline under prolonged heating. This phenomenon arises due to the considerable alterations in boundary conditions. Under this situation, we should account for the influence of low *f* layers (Fig. S24).

Based on the results of nonwritten ARM, we selected 280 °C as the constant temperature. The sample was initially preheated, followed by constant-temperature heating. Figure 3M reveals that upon reaching the phase change threshold, the metallic-to-dielectric transition rate of the top-layer cIST in the written regions exhibits an inverse correlation with laser power. This is because higher writing power corresponds to slower removal of mid-infrared shielding caused by cIST. This establishes a pathway for controlling emissivity evolution through tailored laser power during thermal treatment.

### Tailored infrared emissivity

**Fig. 4.**
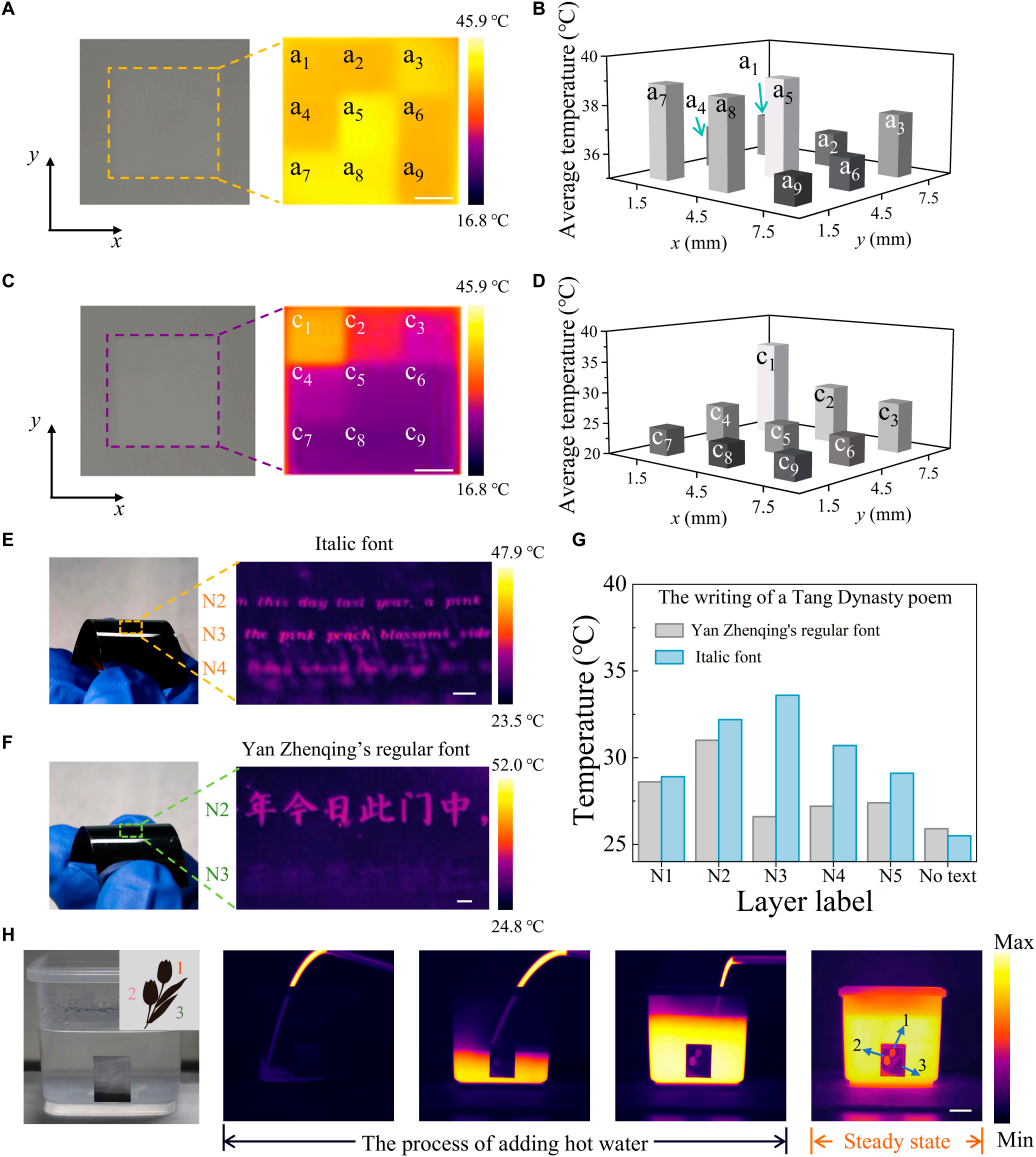
Applications of ARM in information storage and patterning. (A and C) Visible and LWIR images of a mosaic (scale bar, 2 mm). (B and D) Average thermal image temperatures of different mosaic regions (*x* and *y* coordinates of the center positions of different mosaics). (E) Visible and LWIR images of the poem “Written in a Village South of the Capital” in Yan Zhengqing’s regular font (scale bar, 500 μm; flexible substrate). (F) Visible and LWIR images of the poem “Written in a Village South of the Capital” in italic font (scale bar, 500 μm; flexible substrate; fabrication details are in Fig. S21). (G) LWIR image temperatures at selected points in different regions of the poem in 2 different fonts. (H) Presentation of adding hot water to a square box with a tulip pattern (scale bar, 2 cm). Fabrication details are in Text S6.

Benefiting from the above heating-driven atomic rearrangement and high spatial-resolution laser-induced IST phase change, the infrared emissivity can be tailored, thus enabling flexible image and text recording. Figure 4 illustrates the following 2 methods: (a) low-power laser writing (≤27.5 mW), which mainly triggers atomic rearrangement in ARM; (b) high-power laser writing (≥30 mW), which mainly induces varying crystallization degrees of IST. In the case of rewriting,the power range will change, and a smaller power will be required to achieve phase change as the dominant mechanism (Fig. S30).

Initially, mosaic patterns were written on the ARM (silicon substrate) to demonstrate its fundamental multi-bit storage capability. The pattern in Fig. 4A was fabricated using low-power laser writing (≤27.5 mW), whereas Fig. 4C employed higher power (≥30 mW). Corresponding thermal image temperatures of these regions are illustrated in Fig. 4B and D, respectively. Following this, the rigid substrate was extended to a flexible polyimide (PI) film. A miniature region was patterned with the Tang poem “Written in a Village South of the Capital” via laser direct writing (Fig. 4E and F). Italic script was created with low-power writing (≤32 mW), while Yan Zhengqing’s regular script required higher power (≥40 mW). Figure 4G shows the thermal image temperature of the selected points in different text parts (N1 to N5: title and individual verses). Like Fig. 4B and D, different temperatures can correspond to distinct coded bits.

Small-area textual information can be extracted via infrared microscopy, whereas visually discernible patterns are preferable for artistic infrared designs. As shown in Fig. 4H, a tulip motif was fabricated by assigning slightly higher emissivity to 2 petals and lower emissivity to the leaves. A flexible ARM patch bearing this pattern was attached to a square container. Upon pouring hot water to simulate irrigation, the tulip gradually emerged, resembling growth. After stabilization, a well-defined thermal image of the tulip was achieved.

### Infrared information encryption

It is critical to ensure the confidentiality of privacy-sensitive and security-critical information. Building on this motivation, we demonstrate encryption functionality in ARM for written data. The related experimental videos are presented under the themes “Guess who’s hiding in the shadow” and “Which number will appear first” in Movies S1 and S2, respectively. Below is our analysis of the specific experimental process and mechanism.

**Fig. 5.**
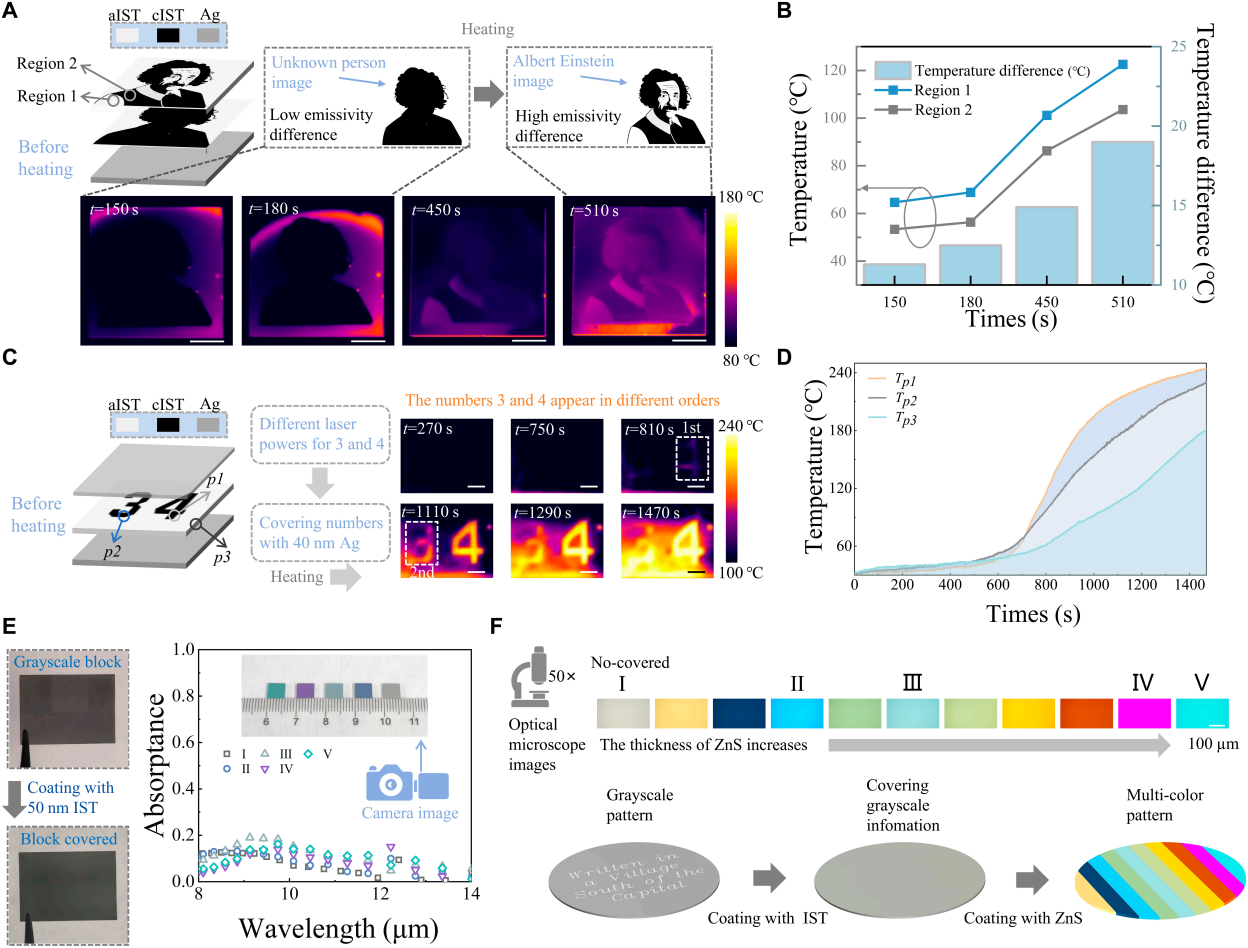
Infrared pattern encryption and visible hiding. (A) Encryption and decryption of the Einstein pattern. (B) Thermal image temperatures and temperature difference of regions 1 and 2. (C) Encryption and decryption of numerical values and their arrangement order. (D) Thermal image temperatures of *p1* to *p3*. (E) Visible hiding in the camera image. (F) Visible hiding in optical microscope images. Fabrication details are in Text S6.

Figure 5A shows the temporal evolution of infrared images during heating. In the early stage of heating (encrypted state), ARM appears as an unknown portrait without facial features. After a period of heating (decryption state), ARM is manifested as an image of Albert Einstein with facial features. The principle is as follows: Before heating, both regions 1 and 2 lack resonant excitation in the LWIR band and exhibit nearly identical spectra, concealing details within the overall pattern. As heating proceeds, Ag incorporation induces a metallic-to-dielectric transition of cIST in region 1, resulting in a rapidly increasing emissivity. In contrast, the cIST layer in region 2 exhibits greater thickness, while its overlying layer remains crystalline, keeping a low emissivity. The emissivity contrast between the 2 regions gradually reveals the hidden image. Figure 5B presents the thermal image temperature and its difference in regions 1 and 2. Notably, the temperature difference undergoes rapid expansion within the 450- to 510-s interval. This observation further confirms the encryption mechanism. In this experiment, regions 1 and 2 exhibit different distributions of aIST and cIST along the direction perpendicular to the sample surface. This configuration is designed to ensure that the initial emissivity difference is sufficiently small while enabling an evident emissivity contrast after a certain period of heating. According to simulation results, the thickness of aIST needs to be less than 320 nm (Fig. S32), yet it should not be too small to ensure adequate difference. Therefore, the experimentally selected thickness of 200 nm is suitable (Figs. S16 and S31).

In Fig. 5C, heating induces the sequential emergence of numbers 4 and 3, encoding both the values and their order. Here, the upper Ag layer shields underlying information, while controlled regional differences in Ag diffusion dictate the reveal sequence. In Fig. 5D, we compare the thermal image temperatures of the *p*1, *p*2, and *p*3 regions. It is worth mentioning that all 3 areas are in a low emissivity state for a long period of time. These results demonstrate that the encryption method exhibits strong resistance to brute-force decryption attempts. Beyond the LWIR regime, it is also necessary to prevent information leakage in visible/near-infrared bands. For example, laser direct writing generates grayscale patterns that, if left uncovered, directly expose the encryption carrier.

Figure 5E demonstrates that a 50-nm IST layer effectively conceals the underlying grayscale pattern, while subsequent ZnS plating enables color control without altering LWIR spectra. To address encryption requiring micro-infrared imaging, ARM with varied ZnS layer thicknesses was characterized via optical mirror analysis. As shown in Fig. 5F, optical microscope images reveal distinct color variations across the ARM coating with ZnS layers of different thicknesses. Based on this phenomenon, we present a schematic that demonstrates a method capable of both covering grayscale patterns and generating colorful designs in the visible band. The process is experimentally feasible, and it entails multiple cycles of ZnS deposition using different photomasks to create the required thickness gradients.

The emissivity of ARM exhibits significant rearrangement-induced alterations, which can be tailored by adjusting the writing power and cIST layer thickness. Based on this tunability, we present a temporal-spatial encryption strategy. The related experimental video is presented under the theme “A funny competition of average emissivity” in Movie S3. Contents provide a detailed analysis of the experimental process and mechanism.

**Fig. 6.**
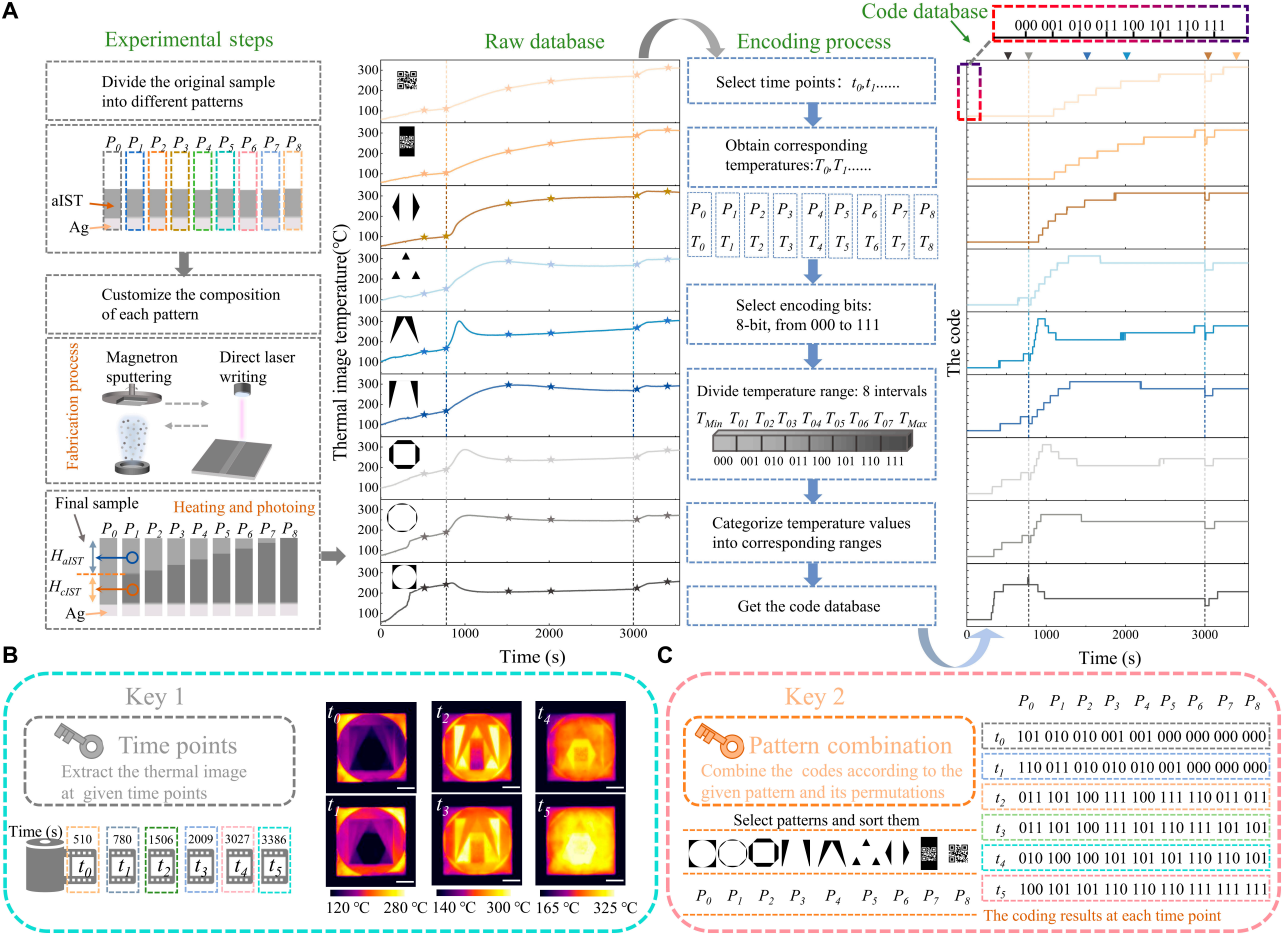
Spatiotemporal encoding encryption. (A) Experimental steps, raw database, encoding process, and code database. (B) Key 1 (time points) and the thermal images (scale bar, 1 cm). (C) Key 2 (patterns combination) and the selected coding. Fabrication details are in Text S6.

Figure 6A shows the customization of ARM via multi-step magnetron sputtering and laser writing (different power levels). This process makes region-dependent variations in both the crystallinity of cIST and the aIST-to-cIST thickness ratio. After the heating process, thermal image temperature variations across different regions were recorded to establish the raw database. By dividing temperature intervals into 8 levels (8 bits), we construct a code database that includes both the encryption content and redundant entries.

Extracting encrypted content from large data requires 2 critical keys. The first key involves time points (Key 1, Fig. 6B), which enable acquisition of LWIR images at distinct heating stages. These images contain regions with different emissivity patterns. The second key employs a pattern combination (Key 2, Fig. 6C), which means that the code data are selectively extracted from the given patterns with the combination sequence. The intentional redundancy in the database enhances spoofing resistance and strengthens encryption security. This advantage originates from the robust dynamic regulation capabilities of the ARM, primarily enabled by its continuous modulation, diverse variations, and large modulation amplitude.

Furthermore, to demonstrate the outstanding performance of our theoretical framework and experimental results, in Table 1, we made comparisons with other distinguished published works across 5 key factors, which demonstrate our advantages in 4 aspects: (a) Higher spatial resolution. Since ARM is compatible with high-precision micro/nanofabrication techniques like laser direct writing, it is easy to write high-resolution (pixel unit < 20 μm) infrared images. This means that ARM can achieve greater clarity and possess higher spatial information density. (b) Higher storage density. Although dynamic modulation allows a single device to store multiple images, its capability remains insufficient. Our approach enables storing an entire dynamic process within one device (calculated at 1 fps), which includes more than 3,500 frames (Fig. 6), greatly boosting the stored data volume. (c) Lower write error rate. As phase changes and atomic rearrangement via laser direct writing fundamentally involve localized heating, the operational temperature window corresponds directly to the laser power range. But additionally and crucially, the ARM exhibits nearly 10 times wider operation temperature range than other devices. This means that within the same error margin, ARM will have fewer write errors, demonstrating superior reliability. (d) More encoding bits. A larger modulation amplitude means that more encoding bits can be obtained at the same readout accuracy. For instance, compared to 50% achieved by a conventional device using dual-band modulation, ARM increases storage density by 29.48% and 47.88% per band, respectively. If a conventional device has sufficient memory cells to reach ultra density optical (UDO) disc capacity (30 GB), then with an identical number of memory cells, applying ARM would increase capacity by 8.844 GB (MWIR) and 14.364 GB (LWIR). This means that the increased storage capacity of a single memory device can hold over 4,000 high-definition resolution photos (1080p). In a word, this work has shown competitive performance across multiple dimensions.

**Table 1.** Comparison table of key factors with other distinguished published works

Work	Pixel unit	Number of encodable images	Operation temperature range	MWIR modulation amplitude	LWIR modulation amplitude
Ref. [5]	>1 cm	5	-	51%	41%
Ref. [23]	>1 mm	7	10 °C	~40.2%	~50%
Ref. [24]	>1 mm	5	-	-	<44.8%
Ref. [37]	~100 μm	3	-	-	~55%
Ref. [38]	>1 cm	2	20 °C	-	60%
Ref. [39]	~100 μm	8	20 °C	-	54%
This work	<20 μm	>3,500	165 °C	64.74%	73.94%

## Discussion

The inevitable phenomenon of interface atomic rearrangement induced by interfacial diffusion has long been perceived as detrimental to photonic devices. Consequently, substantial efforts have been directed toward its suppression, while its potential benefits have been largely overlooked. Herein, we leverage the interfacial atomic rearrangement in Ag–IST to implement a dynamic thermal emission control strategy with integrated temporal dimensionality. To present a spatiotemporal thermal radiation tailoring framework, we need to establish a full dynamic profile of thermal radiation variations. Firstly, conventional radiation control approaches, including stretchable liquid metal microdroplets, 2D materials, and PCM-based methods, often exhibit rapid, discontinuous state transitions. This dynamic characteristic poses considerable challenges for infrared thermography systems to effectively capture the full regulation process. For atomic rearrangement, as a continuous process governed by both rate and time, the average emissivity could change continuously and stably. Secondly, across the conventional techniques, the average mid-infrared emissivity typically demonstrates a monotonic increasing trend with respect to key control parameters. Such behavior fundamentally limits the potential for programming complex systems. As a comparison, the interface atomic rearrangement could achieve nonmonotonic behavior, because the process of atomic migration is programmable and subject to control through modifications of external parameters. Thirdly, for the materials combination, we selected metal Ag and PCM IST. This enables the mixed layer to undergo a considerable change in the imaginary part of the permittivity as *f* increases. Compared to PCMs like GST, which transition from low-loss to high-loss dielectrics, the change in the imaginary part from aIST to Ag–IST (*f* = 0.5) far surpasses it. For example, it is nearly 70 times greater at 14 μm (Fig. 1B). This results in a substantial mid-infrared emissivity variation (1.98% to 66.72% in MWIR and 7.5% to 81.44% in LWIR). In fact, ARM could exhibit even higher modulation amplitudes than this value. As recorded in Fig. 6A, at specific time points, the thermal image temperature of certain regions approached the temperature of the hot plate, demonstrating near-perfect broadband high emissivity.

In constructing the spatiotemporal coding encryption database, we inscribed only one power pattern after each sputtering of an IST film; however, a much richer pattern set is readily attainable by tuning the direct-write power. We adopted an 8-bit code for encoding, yet ARM can feasibly support higher bit depths—for example, 128-bit—because it imposes relatively loose temperature-control precision, requiring only ~0.025× the precision demanded by PCMs such as GST (Table S2). Moreover, the large modulation amplitude confers strong robustness on the resulting code library. By jointly exploiting spatial and temporal degrees of freedom, a single device can thus host a very large spatiotemporal dual-key database, enhancing both the volume of encrypted information and the encryption strength compared with traditional approaches that conceal information in a single static pattern.

In conclusion, we propose a spatiotemporal thermal radiation tailoring strategy based on interface atomic rearrangement. First, we establish a 1D component-rearrangement model to clarify the mechanisms of dynamic regulation. By analyzing the equivalent permittivity of the mixed layer, we simplify the finite-difference time-domain (FDTD) simulation, and we also investigate how laser writing tailors interface atomic migration, revealing the link between phase change and atomic rearrangement. Notably, leveraging these insights, we successfully demonstrate information storage and static encryption. Moreover, we realize a spatiotemporal encryption scheme that, owing to its continuous tunability, exhibits robust anti-counterfeiting capabilities.

Looking ahead, achieving atomic rearrangement at interfaces can also be realized through electric, magnetic, or even multi-field approaches. The materials on either side of the interface offer a vast selection range, for example, metals and PCMs, metals and metals, and PCMs and PCMs. By choosing materials with different optical properties, we can create an extensive library of permittivities that do not exist naturally. Furthermore, the controlling boundary conditions enable higher-dimensional, directional, and asymmetric rearrangement. Owing to the continuous nature of the atomic rearrangement process, the multidimensionality of manipulation methods, and the broadband character of permittivity reconstruction, it is entirely feasible to realize highly flexible, high-resolution spatiotemporal tailoring across an ultra-wide range from ultraviolet (UV) to microwave frequencies. It undoubtedly represents an important milestone supporting subsequent spatiotemporal thermal radiation and photonics research on multi-material systems and ultra-wide bands.

## Methods

### Simulation method

Absorptance characteristics under nonpolarized normal incidence were numerically simulated employing the FDTD method via Lumerical FDTD Solutions. The computational model incorporated a *z*-propagating infrared plane wave incident on the ARM. Periodic boundary conditions were implemented along the *x* and *y* axes, while perfectly matched layers (PMLs) with a mesh convergence factor of 3 were applied at the *z* boundaries. A nonuniform grid configuration with 2-nm resolution in the *z* direction was adopted for enhanced computational precision. The refractive index was adopted from previous studies (for details, see Figs. S3 and S4).

### Manufacturing of ARM

(a) A 4-inch-diameter silicon wafer was cut into 4 × 4 cm^2^ and 0.8 × 0.8 cm^2^ substrates. (b) Sequential ultrasonic cleaning was performed in acetone, methanol, and isopropyl alcohol (IPA) baths for 20 min per solvent, followed by nitrogen drying to eliminate surface contaminants. (c) Silver films were deposited using an FC-2000 electron beam physical vapor deposition system (Edwards, UK) at a constant deposition rate of 2 Å/s under vacuum conditions (<2 × 10^−3^ Pa). (d) An IST layer was sputtered through magnetron sputtering (Nordiko NSC-2000). (e) A stoichiometric target (In:Sb:Te = 3:1:2 at %, 99.99% purity) was employed at a base pressure of 1.0 × 10^−4^ Pa and deposition rate of 5 Å/s. The as-deposited IST alloy exhibits amorphous characteristics.

### Laser direct writing

The laser lithography process was implemented using a DWL 66+ system (Heidelberg Instruments) equipped with a 375-nm UV continuous-wave laser. Key parameters included a 300-nm/200-μm writing line width and adjustable power settings (10 to 100 mW). To account for power dependencies on film thickness, material composition, laser wavelength, and substrate properties, the following calibration protocol was established: (a) Gradient power tests (10-mW increments from 10 mW to 100 mW) were conducted to generate optical density variations across the sample surface. (b) Fourier transform infrared (FTIR) spectra measurements were performed, initiating analysis from the maximum power exposure region. (c) Spectra responses were correlated with surface morphology observations via optical microscopy to nondestructively identify phase transition thresholds and laser-induced damage limits.

### Spectra of ARM

Mid-infrared reflectivity characterization was performed using a Nicolet 5700 FTIR spectrometer (Thermo Fisher Scientific) equipped with a liquid nitrogen-cooled mercury cadmium telluride (MCT) detector. The system was maintained at cryogenic temperatures (77 K) throughout measurements to ensure optimal signal-to-noise ratio and radiometric accuracy.

### Imaging analysis

The infrared characterization system comprised 3 specialized modules from Guide Infrared Co.: (a) a PS610 system for microscale LWIR imaging (8 to 14 μm), (b) a PS680s platform for macroscopic LWIR analysis, and (c) a GAVIN615A imager operating in the MWIR regime (3.7 to 4.8 μm). Complementing these infrared modalities, visible images were acquired using a Canon 200D camera. Surface topological features were quantitatively analyzed with an Olympus BX53M metallurgical microscope, ensuring submicrometer-scale morphological resolution.

### Cross-sectional analysis of ARM

Cross-sectional morphology analysis was performed using a field-emission scanning electron microscope (FEI Helios Nanolab 600i, Thermo Fisher Scientific) operated at 20-kV accelerating voltage with 86-pA probe current.

## Data Availability

All data generated in this study are presented in the paper and the Supplementary Materials. Source data are provided with this paper.

## Supplementary Materials

Supplementary 1 Texts S1 to S12 Figs. S1 to S45 Tables S1 and S2 Movies S1 to S3
