# Supplementary material for: Programmable Interface Atomic Rearrangement for Spatiotemporal Thermal Radiation Tailoring
Source: Research (Wash D C). 2026 Mar 6;9:1141. doi: 10.34133/research.1141 (PMC12963646; doi:10.34133/research.1141)
Supplement: Supplementary 1 — Texts S1 to S12 Figs. S1 to S45 Tables S1 and S2 Movies S1 to S3 [file research.1141.f1.zip › S14.pdf]

The heating temperature of the sample

23℃	160℃	190℃	205℃
220℃	250℃	280℃	295℃
305℃	310℃	316℃	319℃
321℃	322℃	323℃	325℃
