# Supplementary material for: Programmable Interface Atomic Rearrangement for Spatiotemporal Thermal Radiation Tailoring
Source: Research (Wash D C). 2026 Mar 6;9:1141. doi: 10.34133/research.1141 (PMC12963646; doi:10.34133/research.1141)
Supplement: Supplementary 1 — Texts S1 to S12 Figs. S1 to S45 Tables S1 and S2 Movies S1 to S3 [file research.1141.f1.zip › S18.pdf]

Pattern 1

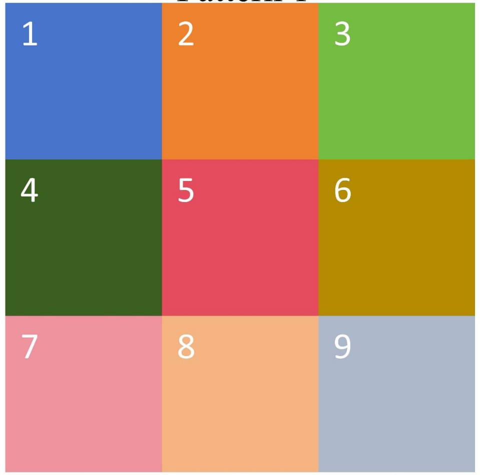

Serial number		1	2	3	4
Power		25 mW	25 mW	26.5 mW	26.5 mW
counts		2	3	1	2
Serial number	5	6	7	8	9
Power	25 mW	25 mW	27.5 mW	26.5 mW	27.5 mW
counts	1	2	1	1	2

Pattern 2

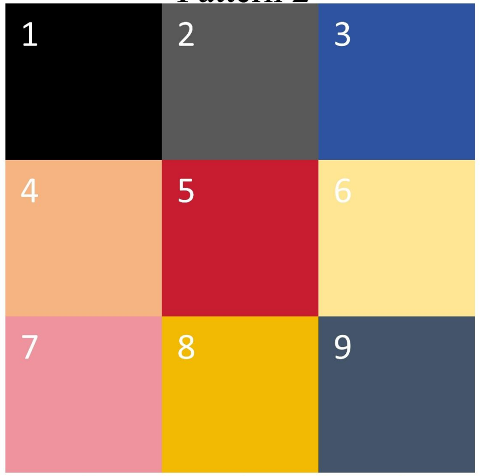

Serial number		1	2	3	4
Power		30 mW	40 mW	45 mW	50 mW
counts		1	1	1	1
Serial number	5	6	7	8	9
Power	60 mW	65 mW	70 mW	80 mW	100 mW
counts	1	1	1	1	1
