# Supplementary material for: Programmable Interface Atomic Rearrangement for Spatiotemporal Thermal Radiation Tailoring
Source: Research (Wash D C). 2026 Mar 6;9:1141. doi: 10.34133/research.1141 (PMC12963646; doi:10.34133/research.1141)
Supplement: Supplementary 1 — Texts S1 to S12 Figs. S1 to S45 Tables S1 and S2 Movies S1 to S3 [file research.1141.f1.zip › S19.pdf]

(1) *Written in a Village South of the Capital*

(2) *In this house on this day last year, a pink face vied*
(3) *In beauty with the pink peach blossoms side by side.*
(4) *I do not know today where the pink face has gone;*
(5) *In the vernal breeze still smile pink peach blossoms full–blown.*

Serial number	(1)	(2)	(3)	(4)	(5)
Power	28 mW	30 mW	27.5 mW	32 mW	27.5 mW
counts	2	1	1	1	3
