# Supplementary material for: Programmable Interface Atomic Rearrangement for Spatiotemporal Thermal Radiation Tailoring
Source: Research (Wash D C). 2026 Mar 6;9:1141. doi: 10.34133/research.1141 (PMC12963646; doi:10.34133/research.1141)
Supplement: Supplementary 1 — Texts S1 to S12 Figs. S1 to S45 Tables S1 and S2 Movies S1 to S3 [file research.1141.f1.zip › S20.pdf]

(1) 题都城南庄

(2) 去年今日此门中，
(3) 人面桃花相映红。
(4) 人面不知何处去，
(5) 桃花依旧笑春风。

Serial number	(1)	(2)	(3)	(4)	(5)
Power	50 mW	40 mW	80 mW	70 mW	60 mW
counts	1	1	1	1	1
