# Supplementary material for: Programmable Interface Atomic Rearrangement for Spatiotemporal Thermal Radiation Tailoring
Source: Research (Wash D C). 2026 Mar 6;9:1141. doi: 10.34133/research.1141 (PMC12963646; doi:10.34133/research.1141)
Supplement: Supplementary 1 — Texts S1 to S12 Figs. S1 to S45 Tables S1 and S2 Movies S1 to S3 [file research.1141.f1.zip › SI-unmarked.pdf]

Supporting information for:

Programmable interface atomic rearrangement for spatiotemporal thermal radiation tailoring

Xinye Liao[1†], Mingyu Luo[2†], Zhaojian Zhang[1†], Qi Jiang[1], Xin Li[1], Junxiang Zeng[1], Yule Wang[3], Xingpeng Jiang[1], Jie Nong[1], Jiagui Wu[4], Dongqing Liu[5], Huan Chen[1*], Xin He[1*], Xiaohu Wu[6], Qiang Li[7*], Junbo Yang[1*]

[1] Center of Material Science, College of Sciences, National University of Defense Technology, Changsha 410073, China;
[2] Photonics Research Institute, Department of Electrical and Electronic Engineering, The Hong Kong Polytechnic University, Kowloon, Hong Kong SAR 999077, China
[3] College of Advanced Interdisciplinary Studies, National University of Defense Technology, Changsha 410073, China;
[4] School of Physical Science and Technology, Southwest University, Chongqing 400715, China;
[5] Science and Technology on Advanced Ceramic Fibers and Composites Laboratory, College of Aerospace Science and Engineering, National University of Defense Technology, Changsha 410073, China;
[6] Thermal Science Research Center, Shandong Institute of Advanced Technology, Jinan 250100, Shandong, China
[7] State Key Laboratory of Extreme Photonic and Instrumentation, College of Optical Science and Engineering, Zhejiang University, Hangzhou 310027, China;
[†] These authors contributed equally to this work
*Corresponding author: H Chen, E-mail: chenhuan11@nudt.edu.cn; X He, E-mail: hexin12a@nudt.edu.cn; Q Li, E-mail: qiangli@zju.edu.cn; J B Yang, E-mail: yangjunbo@nudt.edu.cn

This PDF file includes:

Supplementary Texts 1-12
Supplementary Figures 1 to 45
Supplementary Tables 1 to 2

Other Supplementary Materials for this manuscript include the following:

Supplementary Videos 1 to 3

Contents

Supplementary Text 1 5
Supplementary Text 2 11
Supplementary Text 3 12
Supplementary Text 4 17
Supplementary Text 5 20
Supplementary Text 6 22
Supplementary Text 7 23
Supplementary Text 8 24
Supplementary Text 9 26
Supplementary Text 10 27
Supplementary Text 11 28
Supplementary Text 12 29
Supplementary Figure 1 30
Supplementary Figure 2 31
Supplementary Figure 3 32
Supplementary Figure 4 33
Supplementary Figure 5 34
Supplementary Figure 6 35
Supplementary Figure 7 36
Supplementary Figure 8 37
Supplementary Figure 9 38
Supplementary Figure 10 39
Supplementary Figure 11 40
Supplementary Figure 12 41
Supplementary Figure 13 42
Supplementary Figure 14 43
Supplementary Figure 15 44
Supplementary Figure 16 45
Supplementary Figure 17 46
Supplementary Figure 18 47
Supplementary Figure 19 48
Supplementary Figure 20 49
Supplementary Figure 21 50
Supplementary Figure 22 51
Supplementary Figure 23 52
Supplementary Figure 24 53
Supplementary Figure 25 54
Supplementary Figure 26 55
Supplementary Figure 27 56
Supplementary Figure 28 57
Supplementary Figure 29 58
Supplementary Figure 30 59
Supplementary Figure 31 60

Supplementary Figure 32 61
Supplementary Figure 33 62
Supplementary Figure 34 63
Supplementary Figure 35 64
Supplementary Figure 36 65
Supplementary Figure 37 66
Supplementary Figure 38 67
Supplementary Figure 39 68
Supplementary Figure 40 69
Supplementary Figure 41 70
Supplementary Figure 42 71
Supplementary Figure 43 72
Supplementary Figure 44 73
Supplementary Figure 45 74
Supplementary Table 1 75
Supplementary Table 2 76
Reference 77

Supplementary Text 1

Equivalent permittivity of subwavelength metal-dielectric mixed film

In the study of the optical properties of metal-dielectric mixed materials, we can assume that the mixed materials are composed of pure metal and dielectric. When the target wavelength size is much larger than the cluster, the electromagnetic response of a single metal or dielectric cluster is masked by the comprehensive response of the entire system. In this case, the optical properties of microscopic inhomogeneous composite materials can be studied by solving the macroscopic effective dielectric function. This method is called the effective medium approach[1], which uses the permittivity of each material and its volume filling fraction to obtain the effective dielectric function.

Starting from single molecules, we analyze the local electromagnetic field characteristics. We assume polarized molecules are in a small spherical cavity, large enough on a microscopic scale that the outside can be considered as a medium with uniform polarization along the x direction. The local electric field in the cavity is set to E_{in}. Since there's only vacuum between single molecules, the cavity is considered to be in a vacuum state.

As shown in **Supplementary Fig. 1**, the local field acting on the central dipole can be decomposed into three components: E_0, E_1, and E_2. These satisfy $E_{in}=E_0+E_1+E_2$, where E_0 is the external electric field, E_1 is the polarization electric field induced by the external electric field in the external medium (making the actual electric field in the external medium $E=E_0+E_1$), and E_2 is the electric field generated by the polarization charge on the cavity surface within the cavity. P_0 represents the polarization intensity of the external medium. Since the cavity is under vacuum, the surface only has polarization charges generated by external P_0. We define the vacuum permittivity ε_o and the cavity radius R, the density can be expressed as:

$$\sigma_P = P_n = P_0 \cos\theta \tag{1}$$

The charge on the annular band between θ and $\theta+d\theta$ is therefore given by:

$$\mathrm{d}q = 2\pi R^2 P_0 \cos\theta \sin\theta d\theta \tag{2}$$

Thus, the potential at any point on the surface of the cavity along the x-axis is given by:

$$d\varphi = \frac{1}{4\pi\varepsilon_0} \frac{2\pi R^2 P_0 \cos\theta \sin\theta d\theta}{(x^2+R^2-2xR\cos\theta)^{1/2}} \tag{3}$$

Integrating the total potential generated by the surface charges on the cavity yields:

$$\varphi(x) = -\frac{p_0 R^2}{2\varepsilon_0}\int_0^{\pi}\frac{\cos\theta\sin\theta d\theta}{(x^2+R^2-2xR\cos\theta)^{1/2}} \tag{4}$$

Since the observation point is inside the cavity($x < R$), the integral result is:

$$\varphi(x) = -\frac{P_0 x}{3\varepsilon_0} \tag{5}$$

Therefore, the electric field inside the cavity due to the polarization charges is:

$$E(x) = -\frac{\partial\varphi}{\partial x} = \frac{P_0}{3\varepsilon_0} \tag{6}$$

This is independent of the position, and at this point, the magnitude of the local electric field can be determined:

$$E_{in} = E + \frac{P_0}{3\varepsilon_0} \tag{7}$$

Where E_{in} is the field acting on the atomic positions in a cubic lattice.

We relate the polarization strength P_0 to the electric dipole moment of each molecule. Let α represent the polarizability of a molecule, then the macroscopic polarizability P_0 can be expressed as:

$$P_0 = N\alpha E_{in} = N\alpha(E + \frac{P_0}{3\varepsilon_0}) \tag{8}$$

where N is the volume density of electric dipoles. Additionally, the fundamental relationship between electric displacement D, polarization density P, and electric field strength E can be expressed as:

$$D = \varepsilon_0 E + P = \varepsilon_0(1+\chi_e)E = \varepsilon_0\varepsilon_r E \tag{9}$$

where ε_r is the relative permittivity of the external uniform medium, and χ_e is the electric susceptibility. From this, we can obtain:

$$\frac{N\alpha}{3\varepsilon_0} = \frac{\varepsilon_r - 1}{\varepsilon_r + 2} \tag{10}$$

The molecular polarizability is:

$$\alpha = \frac{3\varepsilon_0}{N}\frac{\varepsilon_r - 1}{\varepsilon_r + 2} \tag{11}$$

It illustrates the response of individual molecules to an electric field. If we apply this concept to a macroscopic ensemble, we can consider a mixture of material inclusions with relative permittivity ε_1 and a surrounding material with relative permittivity ε_2. Let f be the volume filling fraction of the inclusions, and let the mixture have an effective relative permittivity ε. Consider a spherical cavity of radius R within the mixture, as shown in **Supplementary Fig. 2**, where the interior is filled with the mixture and the exterior is a uniformly polarized medium with relative permittivity ε_h.

At this point, the equivalent situation shifts from solving for polarized molecules in a vacuum cavity (with a uniformly polarized medium outside) to solving for a mixture of inclusions within an external uniform dielectric material. First, consider the mixture of inclusions as a whole. Since the cavity is no longer in a vacuum state, an additional polarization P_1 is induced. With the external polarization strength being P_0, the actual polarization strength is $P_{\Delta 1} = P_1 - P_0$. Substituting p_0, p_1 into equation SI9 yields:

$$\begin{cases} \varepsilon_0 E + P_1 = \varepsilon_0 \varepsilon E \\ \varepsilon_0 E + P_0 = \varepsilon_0 \varepsilon_h E \end{cases} \tag{12}$$

Taking the difference, we obtain:

$$P_{\Delta 1} = (\varepsilon - \varepsilon_h)\varepsilon_0 E \tag{13}$$

The external dielectric environment has changed from the vacuum state originally occupied by polarized molecules to a uniform medium with a relative permittivity of ε_h. This means that the electric field inside the cavity due to polarization charges has changed, which can be modified as:

$$E_2 = \frac{P_{\Delta 1}}{3\varepsilon_h \varepsilon_0} \tag{14}$$

By solving equations SI 7, 8, 9 and 13 simultaneously, we can obtain:

$$\frac{N\alpha}{3\varepsilon_0\varepsilon_h}=\frac{\varepsilon-\varepsilon_h}{\varepsilon+2\varepsilon_h} \tag{15}$$

Next, we consider the inclusion material with a permittivity of ε_1. Since the volume filling fraction of this material is f, and in this assumption, α represents only the polarizability of a single molecule of the inclusion material, the volume occupied by a single molecule change from $\frac{1}{N}$ to $\frac{f}{N}$. At this point, the equivalent situation shifts from solving for a mixture of inclusions within an external uniform dielectric material to solving for a single inclusion material within an external uniform dielectric material. In this case, an additional polarization P_2 is induced, with the external polarization strength being P_0. The actual polarization strength is $P_{\Delta 2}=P_2-P_0$, and its relationship with the single-molecule polarizability becomes $P_0=\frac{N}{f}\alpha E_{in}$. Solving equations SI 7 and 9 simultaneously yields:

$$\frac{N\alpha}{3\varepsilon_0\varepsilon_h f}=\frac{\varepsilon_1-\varepsilon_h}{\varepsilon_1+2\varepsilon_h} \tag{16}$$

Combining equations SI 15 and 16, we can obtain:

$$\frac{\varepsilon-\varepsilon_h}{\varepsilon+2\varepsilon_h}=f\frac{\varepsilon_1-\varepsilon_h}{\varepsilon_1+2\varepsilon_h} \tag{17}$$

When considering the effective permittivity of a mixture, it is necessary to account for the effects brought by all materials. To address this, we can modify the equation through symmetric treatment.[1]

First, assume that the two composite materials are spherical particles with relative permittivity ε_1 and ε_2, respectively, dispersed in a matrix with a permittivity of ε_h. The volume filling fractions of the two inner materials are f_1 and f_2 ($f_1+f_2=1$), respectively. The corresponding single-molecule polarizabilities are α_1 and α_2. Similarly, we can obtain the following equation:

$$\begin{cases} \dfrac{\varepsilon-\varepsilon_h}{\varepsilon+2\varepsilon_h}=\dfrac{N\alpha_1}{3\varepsilon_0\varepsilon_h}+\dfrac{N\alpha_2}{3\varepsilon_0\varepsilon_h} \\ \dfrac{N\alpha_1}{3\varepsilon_0\varepsilon_h f}=\dfrac{\varepsilon_1-\varepsilon_h}{\varepsilon_1+2\varepsilon_h} \\ \dfrac{N\alpha_2}{3\varepsilon_0\varepsilon_h f}=\dfrac{\varepsilon_2-\varepsilon_h}{\varepsilon_2+2\varepsilon_h} \end{cases} \tag{18}$$

From equation SI18, we can obtain:

$$\frac{\varepsilon-\varepsilon_h}{\varepsilon+2\varepsilon_h}=f_1\frac{\varepsilon_1-\varepsilon_h}{\varepsilon_1+2\varepsilon_h}+f_2\frac{\varepsilon_2-\varepsilon_h}{\varepsilon_2+2\varepsilon_h} \tag{19}$$

This equation represents the result for materials in a three-dimensional state. For different dimensional conditions, $\varepsilon_i+2\varepsilon_j$ should be replaced with $\varepsilon_i+(d-1)\varepsilon_j$ [2]. After substitution, the equation is as follows:

$$\frac{\varepsilon-\varepsilon_h}{\varepsilon+2\varepsilon_h}=f_1\frac{\varepsilon_1-\varepsilon_h}{\varepsilon_1+(d-1)\varepsilon_h}+f_2\frac{\varepsilon_2-\varepsilon_h}{\varepsilon_2+(d-1)\varepsilon_h} \tag{20}$$

Where *i* and *j* are arbitrary indices, and *d* is the dimension. In the case of composite materials where the thickness is much smaller than the target wavelength, they should be considered as two-dimensional films. Therefore, when the film thickness is much smaller than the target wavelength, *d*=2. The expression is as follows:

$$\frac{\varepsilon-\varepsilon_h}{\varepsilon+2\varepsilon_h}=f_1\frac{\varepsilon_1-\varepsilon_h}{\varepsilon_1+\varepsilon_h}+f_2\frac{\varepsilon_2-\varepsilon_h}{\varepsilon_2+\varepsilon_h} \tag{21}$$

Considering that the external uniform medium is also the mixed material, we can get $\varepsilon=\varepsilon_h$. Therefore, the following expression can be obtained:

$$f_1\frac{\varepsilon_1-\varepsilon}{\varepsilon_1+\varepsilon}+f_2\frac{\varepsilon_2-\varepsilon}{\varepsilon_2+\varepsilon}=0 \tag{22}$$

Solving this, we obtain:

$$\varepsilon=\frac{1}{2}\left\{(2f_1-1)\varepsilon_1+(2f_2-1)\varepsilon_2\pm\sqrt{\left[(2f_1-1)\varepsilon_1+(2f_2-1)\varepsilon_2\right]^2+4\varepsilon_1\varepsilon_2}\right\} \tag{23}$$

Among these two complex solutions, the one with a negative imaginary part must be discarded.

When we set ε_1 to represent the metal permittivity and ε_2 to represent the dielectric permittivity, since the absolute value of the real part of ε_1 will be

much greater than that of ε_2, it is not difficult to see from equation SI23 that there is a zero point for the real part near f_1=0.5. In other words, as the volume filling fraction of metal increases near zero point, the composite material will change from dielectric to metallic characteristics. The physical implication is that as the volume filling fraction of metal increases, continuous metallic pathways form within the sample, creating an infinite cluster of metal components[3].

The permittivity of the materials used in the simulation are shown in **Supplementary Figs. 3 and 4**. At the same time, we calculated the permittivity of the Ag-IST and cIST-aIST mixed layers, which is shown in **Supplementary Fig. 5 and 6**. Through analysis, it can be seen that the cIST-aIST permittivity is equal to 0 is when f is between 0.6 and 0.7.

Supplementary Text 2

Calculation of the skin depth for thin films

When light with intensity I_0 propagates a distance z in a medium, the intensity decreases to $I_0 e^{(-\frac{z}{l})}$, where l is the skin depth. This means that when $Z=l$, the light intensity decays to $\frac{1}{e}$ of its initial value. Since the variation of the electric field E along the propagation direction satisfies the equation $E = E_0 e^{\left[\frac{i2\pi(n'+in'')z}{\lambda}\right]}$, where E_0 is the initial electric field strength and λ is the wavelength of the incident light, and the light intensity is proportional to $|E|^2$, the expression for the skin depth is as follows:

$$l = \frac{\lambda}{4\pi n''} \tag{24}$$

The relationship between the refractive index and the permittivity is as follows:

$$\begin{cases} n'^2 = \dfrac{\left[\varepsilon' + \sqrt{\varepsilon'^2 + \varepsilon''^2}\right]}{2} \\ n''^2 = \dfrac{\left[-\varepsilon' + \sqrt{\varepsilon'^2 + \varepsilon''^2}\right]}{2} \end{cases} \tag{25}$$

Where n' and n'' are the real and imaginary parts of the material's refractive index, respectively, while ε' and ε'' are the real and imaginary parts of the material's permittivity.

By substituting the permittivity of the material, the skin depth of Ag and cIST as well as Ag-IST with different Ag volume filling fraction *f* can be calculated, and the results are shown in **Supplementary Figs. 7 and 8**. The Ag-IST with a high volume filling fraction (*f* = 0.5) exhibits a reduced skin depth. Unlike conventional metallic layers with high mid-infrared reflection, this behavior arises from its exceptional electromagnetic loss capacity.

Supplementary Text 3

The relationship between the position and mass concentration distribution

When there is a difference in solid composition, atoms will move from areas of high concentration to areas of low concentration, and the flux of atoms during diffusion is directly proportional to the gradient of mass concentration. This is known as Fick's first law[4]:

$$J=-D\frac{\mathrm{d}\rho}{\mathrm{d}x} \tag{26}$$

Where J is the diffusion flux, D is the diffusion coefficient, and ρ is the mass concentration of the substance.

Combining Fick's first law with the principle of conservation of mass also leads to Fick's second law[5]:

$$\frac{\partial\rho}{\partial t}=D\frac{\partial^2\rho}{\partial x^2} \tag{27}$$

For ARM, within an appropriate heating range, both the migration of Ag into the top layer of IST and the infiltration of IST into the underlying Ag layer remain minimal. Under these conditions, we can assume that the ends of the double layers system always maintain their original concentrations. This satisfies the boundary condition where the composition at both ends is unaffected by atomic migration. The initial mass concentration of the material at either end is ρ_i, and at the interface, we can consider the mass concentration of any substance to be $0.5\rho_i$, setting this point as x=0. This problem can be transformed into solving the following differential equation:

$$\begin{cases} \frac{\partial\rho}{\partial t}-a^2\frac{\partial^2\rho}{\partial x^2}=0 \\ \rho\,|_{x=0}=\rho_0 \\ \rho\,|_{t=0}=0 \end{cases} \tag{28}$$

Let $D=a^2$, $\rho_0=\frac{\rho_i}{2}$, $\rho(x,t)=\rho_0+u(x,t)$, we can obtain:

$$
\begin{cases}
\frac{\partial u}{\partial t} - a^2 \frac{\partial^2 u}{\partial x^2} = 0 \\
u|_{x=0} = 0 \\
u|_{t=0} = -\rho_0
\end{cases}
\tag{29}
$$

This step converts the non-homogeneous equation into a homogeneous one. It is evident from this equation that it satisfies odd extension, which can be further simplified to:

$$
\begin{cases}
\frac{\partial u}{\partial t} - a^2 \frac{\partial^2 u}{\partial x^2} = 0 \\
u|_{t=0} = \begin{cases} -\rho_0 (x > 0) \\ +\rho_0 (x < 0) \end{cases}
\end{cases}
\tag{30}
$$

let $\varphi(x) = u|_{t=0}$

Applying the Fourier Transform yields:

$$
\begin{cases}
U' + k^2 a^2 U = 0 \\
U|_{t=0} = \varphi(k)
\end{cases}
\tag{31}
$$

The solution to the initial value problem of this ordinary differential equation is given by $U(x,t) = \varphi(k) e^{-k^2 a^2 t}$, applying the inverse Fourier Transform, we obtain:

$$
u(x,t) = \frac{1}{2\pi} \int_{-\infty}^{\infty} \left[\int_{-\infty}^{\infty} \varphi(\xi) e^{-ik\xi} d\xi \right] e^{-k^2 a^2 t} e^{ikx} dk \tag{32}
$$

To simplify the integral using integration equations, we first exchange the order of integration:

$$
u(x,t) = \frac{1}{2\pi} \int_{-\infty}^{\infty} \varphi(\xi) \left[\int_{-\infty}^{\infty} e^{-k^2 a^2 t} e^{ik(x-\xi)} dk \right] d\xi \tag{33}
$$

Let $\alpha = a\sqrt{t}$, $\beta = i(x - \xi)$, and Using the integration equation $\int_{-\infty}^{\infty} e^{-\alpha^2 k^2} e^{\beta k} dk = \frac{\sqrt{\pi}}{\alpha} e^{\frac{\beta^2}{4\alpha^2}}$,

we finally arrive at:

$$u(x,t)=\int_{-\infty}^{\infty}\varphi(\xi)\left[\frac{1}{2a\sqrt{\pi t}}e^{-\frac{(x-\xi)^2}{4a^2t}}\right]d\xi \tag{34}$$

Substitute ($\varphi(x)=u|_{t=0}$)

$$u(x,t)=\int_{-\infty}^{0}\rho_0\left[\frac{1}{2a\sqrt{\pi t}}e^{-\frac{(x-\xi)^2}{4a^2t}}\right]d\xi-\int_{0}^{\infty}\rho_0\left[\frac{1}{2a\sqrt{\pi t}}e^{-\frac{(x-\xi)^2}{4a^2t}}\right]d\xi \tag{35}$$

To connect the two integrals into a single integral expression and eliminate the introduced ξ , in the first integral on the right-hand side of the equation, let $z=(x-\xi)/2a\sqrt{t}$, and in the second integral, let $z=(\xi-x)/2a\sqrt{t}$. This yields:

$$u(x,t)=\frac{\rho_0}{\sqrt{\pi}}\int_{-x/2a\sqrt{t}}^{x/2a\sqrt{t}}\left[\frac{1}{2a\sqrt{\pi t}}e^{-z^2}\right]dz \tag{36}$$

Here, we successfully transform the infinite upper limit in the integral into a finite value. Then, using the even function property of the integrand, the equation simplifies to:

$$u(x,t)=\frac{2\rho_0}{\sqrt{\pi}}\int_{0}^{x/2a\sqrt{t}}\left[\frac{1}{2a\sqrt{\pi t}}e^{-z^2}\right]dz \tag{37}$$

At this point, the variable x only appears explicitly in the upper limit of the integral. To eliminate *z*, we introduce the error function $erf\left(x\right)=\frac{2}{\sqrt{\pi}}\int_0^x e^{-z^2}dz$, and then substitute the initial data values to obtain the final expression:

$$\rho(x,t)=\frac{\rho_i}{2}\left[1-erf(\frac{x}{2\sqrt{Dt}})\right] \tag{38}$$

Furthermore, when the substrate Ag in ARM is sufficiently thick such that the thickness of the IST layer is much smaller than that of the substrate, the initial concentration within the Ag layer is $\rho(x=0,t=0)=\rho,\rho(x\neq 0,t\neq 0)=0$ and satisfies the following differential equation:

$$\begin{cases} \frac{\partial \rho}{\partial t} - a^2 \frac{\partial^2 \rho}{\partial x^2} = 0 \\ \rho |_{t=0} = \begin{cases} \rho_{eff} \delta(x-0), x > 0 \\ \rho_{eff} \delta(x+0), x < 0 \end{cases} \end{cases} \tag{39}$$

The initial equivalent mass concentration coefficient is ρ_{eff}, and the global integral over $\rho|_{t=0}$ corresponds to a double integration of the δ function. Using the previously obtained equation SI34, we can solve for:

$$\rho(x,t) = 2\int_{-\infty}^{\infty} \rho_{eff} \delta(\xi) \left[\frac{1}{2a\sqrt{\pi t}} e^{-\frac{(x-\xi)^2}{4a^2 t}} \right] d\xi = \frac{\rho_{eff}}{a\sqrt{\pi t}} e^{\frac{-x^2}{4a^2 t}} \tag{40}$$

The solution under this equivalent condition is often related to the initial mass per unit area M_i of the thin film source. According to the law of conservation of mass, integrating over the system after time t satisfies:

$$M_i = \int_0^{\infty} \rho(x,t) dx \tag{41}$$

let $\tau^2 = \frac{x^2}{4a^2 t}$, substituting into equation SI40 and 41, we obtain:

$$M_i = \frac{2\rho_{eff}}{\sqrt{\pi}} \int_0^{\infty} e^{-\tau^2} d\tau = 2\rho_{eff} \tag{42}$$

That is

$$\rho_{eff} = \frac{M_i}{2} \tag{43}$$

Substituting the respective data into equation SI40, we obtain the final expression（Shown in **Supplementary Fig. 9**）:

$$\rho(x,t) == \frac{M_i}{2\sqrt{\pi D t}} e^{\frac{-x^2}{4Dt}} \tag{44}$$

This represents the mass concentration distribution when the thickness of the IST layer is much smaller than that of the substrate Ag. It is important to note that, according to the expression, a concentration distribution exists immediately after atomic rearrangement begins, which is unreasonable. This

implies that the sufficient heating time must satisfy $t > \frac{h^2}{2D}$ (h is the thickness of the IST film) to make the solution have adequate precision[6], because Fick's first law is a statistical rule that does not account for the inertia of individual atomic motions. Hence, the atomic migration rate cannot be infinitely large.

Additionally, the diffusion coefficients follow the Arrhenius equation:

$$D = D_0 e^{-\frac{Q}{RT}} \tag{45}$$

To demonstrate the extent of changes in the diffusion coefficient with temperature, we consider volume diffusion where the diffusing element is Ag and the matrix metal is also Ag. The activation energy $Q_{Ag} = 1.9 \times 10^5 \text{J/mol}$, the gas constant $R = 8.314\text{J/}(\text{mol} \cdot \text{K})$, D_0 is the diffusion constant, and T is the absolute temperature of the object. This leads to the expression for lg($D_T/D_{23°C}$) (Shown in **Supplementary Fig. 10**), It can be found that a small temperature increase will lead to a sharp increase in the diffusion coefficient.

Supplementary Text 4

The application of the transfer matrix method in ARM

In isotropic thin-film systems, the propagation characteristics of electromagnetic waves can be solved using the transfer matrix method (See **Supplementary Fig. 11** for schematic diagram). For such systems, incident electromagnetic waves of different polarizations are equivalent. Thus, we may consider a polarization direction as *p*. When an electromagnetic wave enters from a medium with refractive index n_0 into a layer with refractive index n_1 and then exits to a medium with refractive index n_2, its electric and magnetic field intensities change from E_0 and H_0 to E_2 and H_2. By solving the boundary conditions at the top and bottom interfaces of the N_1 layer, we obtain:

$$\begin{bmatrix} E_0 \\ H_0 \end{bmatrix} = \begin{bmatrix} \cos\alpha_1 & \frac{i}{\kappa_1}\sin\alpha_1 \\ i\kappa_1\sin\alpha_1 & \cos\alpha_1 \end{bmatrix} \begin{bmatrix} E_2 \\ H_2 \end{bmatrix} \tag{46}$$

The phase change through the Nj medium layer is given by $\alpha_j = \frac{2\pi}{\lambda_j} n_j d_j \cos\theta_j$, where λj is the wavelength of the electromagnetic wave, dj is the thickness of the *j*-th medium layer, and θj is the incidence angle at the *j*-th medium layer. The admittance of the *j*-th medium layer, denoted as κ_j, is represented by $\kappa_j = n_j \cos\theta_j$.

This transfer process is repeated until the electromagnetic wave exits from the *i*-th layer, and we can obtain:

$$\begin{bmatrix} E_0 \\ H_0 \end{bmatrix} = \left\{ \prod_{j=1}^{k} \begin{bmatrix} \cos\alpha_j & \frac{i}{\kappa_j}\sin\alpha_j \\ i\kappa_j\sin\alpha_j & \cos\alpha_j \end{bmatrix} \right\} \begin{bmatrix} E_{k+1} \\ H_{k+1} \end{bmatrix} \tag{47}$$

The characteristic matrix can be extracted as follows:

$$\begin{bmatrix} A \\ B \end{bmatrix} = \left\{ \prod_{j=1}^{k} \begin{bmatrix} \cos\alpha_j & \frac{i}{\kappa_j}\sin\alpha_j \\ i\kappa_j\sin\alpha_j & \cos\alpha_j \end{bmatrix} \right\} \begin{bmatrix} 1 \\ \kappa_{k+1} \end{bmatrix} \tag{48}$$

The intensity reflection can be expressed as:

$$R = \left(\frac{\kappa_0 A - B}{\kappa_0 A + B}\right)\left(\frac{\kappa_0 A - B}{\kappa_0 A + B}\right)^* \tag{49}$$

This method is applicable to continuous, uniform, isotropic thin-film systems[7], and it can be used to calculate the simplified ARM model. Compared with the FDTD simulation results, we can find that the calculated results are almost consistent (**Supplementary Fig. 12**). For layers where the refractive index varies with the vertical distance of the film, calculations must be made using differential methods. In this work, we can divide the mixed layer of ARM into N parts, with the mixed layer thickness being L_1 and the amorphous IST layer thickness being L_2. Then, the thickness of each equivalent layer is $dL = \frac{L_1}{N}$. If the conditions at both ends are not affected by the diffusion, the volume filling fraction of metallic silver in the j-th layer can be calculated by the equation SI38, that is:

$$f_j = \frac{\rho(x,t)_n}{\rho_i} = \frac{1}{2}\left[1 - erf\left(\frac{jdL}{2\sqrt{Dt}}\right)\right] \tag{50}$$

By combining equations SI23 and SI25, and considering the IST layer, mixed layer, and underlying silver layer of ARM, the characteristic matrix can be expressed as:

$$\begin{cases} M_0 = \begin{bmatrix} \cos\frac{2\pi}{\lambda} n_{IST} L_2 \cos\theta & \frac{i}{\kappa_{IST}} \sin\frac{2\pi}{\lambda} n_{IST} L_2 \cos\theta \\ i\kappa_{IST} \sin\frac{2\pi}{\lambda} n_{IST} L_2 \cos\theta & \cos\frac{2\pi}{\lambda} n_{IST} L_2 \cos\theta \end{bmatrix} \\ M_{1-N} = \prod_{j=1}^{N} \begin{bmatrix} \cos(\frac{2\pi}{\lambda} n_j dL \cos\theta) & \frac{i}{\kappa_j} \sin(\frac{2\pi}{\lambda} n_j dL \cos\theta) \\ i\kappa_j \sin(\frac{2\pi}{\lambda} n_j dL \cos\theta) & \cos(\frac{2\pi}{\lambda} n_j dL \cos\theta) \end{bmatrix} \\ \begin{bmatrix} A \\ B \end{bmatrix} = M_0 M_{1-N} \begin{bmatrix} 1 \\ \kappa_{Ag} \end{bmatrix} \end{cases} \tag{51}$$

Where θ represents the incidence angle, λ is the wavelength of the incident electromagnetic wave, κ_{Ag} represents the admittance of silver, while n_{IST} and κ_{IST} denote the refractive index and admittance of the amorphous IST layer,

respectively. Similarly, n_j and κ_j indicate the refractive index and admittance of the j-th equivalent layer, respectively. By combining equations S49 and S51, the reflection spectra of ARM can be obtained.

Supplementary Text 5

The power received by the infrared camera

At room temperature, the power received by a pixel of an infrared camera can be divided into two parts (See **Supplementary Fig. 13** for the actual schematic diagram) : the thermal radiation Ps from the object itself and the reflection P_r of external thermal sources. For the self-thermal radiation part, the radiant power is related to the object's own temperature Ts and the average emissivity $\bar{\varepsilon}_{\lambda_1\text{-}\lambda_2}$. Within the wavelength range [λ_1, λ_2], the average emissivity is calculated as follows:

$$\bar{\varepsilon}_{\lambda_1\text{-}\lambda_2} = \frac{\int_{\lambda_1}^{\lambda_2} \varepsilon(\lambda) I(\lambda, T_s) d\lambda}{\int_{\lambda_1}^{\lambda_2} I(\lambda, T_s) d\lambda} \tag{52}$$

where $I(\lambda,T) = \dfrac{2\pi hc^2}{\lambda^5[e^{(\frac{hc}{\lambda k_B T_s})} - 1]^{-1}}$ represents the blackbody radiation power, and

$\varepsilon(\lambda)$ is the emissivity of the sample at a specific wavelength, T_s is the temperature of the object, h is the Planck constant, c is the speed of vacuum light, k_B is the Boltzmann constant, and λ is the wavelength of the emission light.

According to Kirchhoff's law[8], the emissivity of an object is equal to its absorptance, that is $\varepsilon(\lambda)=A$, therefore, the emissivity $\varepsilon(\lambda)$ can be calculated from the absorptance. Under the condition of a metallic substrate, the transmissivity T is almost zero. Hence, the absorptance can be represented by $A=1-R$, where R is the reflection of the sample. Similarly, the spontaneous radiation power is as follows:

$$P_s = \int_{\lambda_1}^{\lambda_2} \varepsilon(\lambda, T_s) I(\lambda, T_s) d\lambda \tag{53}$$

For the reflected amount from external thermal sources, it has a similar form, with the emissivity part modified to the product of the reflection and the thermal source emissivity $(1-\varepsilon(\lambda))\varepsilon_r$. The temperature of the heat source is changed from the self-temperature to the external temperature Tr . The expression can be obtained as follows:

$$P_r = \int_{\lambda_1}^{\lambda_2} [1 - \varepsilon(\lambda, T_s)] \varepsilon_r(\lambda, T_r) I(\lambda, T_r) d\lambda \tag{54}$$

The total power received by the infrared camera can be expressed as:

$$P_t = P_s + P_r \tag{55}$$

It is worth mentioning that when selecting external temperature values, they can often be divided into indoor and outdoor scenarios. For indoor conditions, the radiation source can be selected as the building itself, which typically has a room temperature. Under outdoor conditions, due to the characteristics of the atmospheric window, if there are no clouds obstructing, the external radiation source can be considered as the low-temperature space environment, under which the reflected amount from external heat sources is almost zero. In the reception of infrared cameras, heat collection is also related to the collection angle and transmission loss. By integrating over the angle, the power collected by the infrared camera can be obtained:

$$P_{IR} = C_i \int_0^{2\pi} d\varphi \int_{\theta_i}^{\theta_j} \sin\theta d\theta P_t \tag{56}$$

where C_i is the loss coefficient, which varies under different environmental conditions. The range [θ_i, θ_j] represents the collection angle range of the infrared camera, and the emissivity is taken at the integrated angles.

Supplementary Text 6

Details on preparation and results presentation

The preparation process involves variations in layer morphology, film thickness, and laser power, as comprehensively illustrated in **Supplementary Figs. 15–21**. Figure 1 (in the manuscript) presents samples heat-treated at different temperatures; **Supplementary Fig. 14** specifies the exact temperature assigned to each sample.

Supplementary Text 7

The models establishment and expansion of ARM

In the simulation and experimental validation of ARM, two models with distinct film thicknesses (model1 and model2) were analyzed, as schematically illustrated in **Supplementary Fig. 22**. Under identical heating conditions, ARM exhibited divergent behaviors before and after laser writing. Prior to laser writing, it just followed a standard diffusive process. Conversely, post-writing introduced three distinct characteristics: (1) the overlying cIST layer shielded the underlying film; (2) laser writing induced thermal diffusion; and (3) The diffusion rate of the written area will change.

Supplementary Fig. 27 elucidates the impact of preheating on spectra changes in the laser-written region following thermal diffusion. By comparing preheated and non-preheated samples, we observed significant variations in both MWIR and LWIR ranges. This finding suggests that the heating methodology directly affects the final infrared signature, thereby enhancing the encryption capacity of ARM.

To validate the underlying principles of ARM, **Supplementary Figure 26** explores its versatility across metal substrates. Here, the bottom silver layer was replaced with gold and nickel, and absorptance spectra of these modified samples were measured before and after heating. Results confirmed persistent diffusion behaviors across different substrates. Notably, substrate alteration influenced the diffusion rate, as evidenced by changes in spectral amplitude. Additionally, comparisons of heating in nitrogen versus ambient air revealed negligible atmospheric effects on the absorptance spectra, underscoring the robustness of ARM under varying gas environments.

Supplementary Figs. 25 and **26** further demonstrate the scalability of our model. Even with combinations of different metallic and dielectric materials, the spectra of the device can still be significantly tuned via interface atomic rearrangement.

Supplementary Text 8

The other solutions to control atomic rearrangement

The phase change from aIST to cIST can be achieved by using a continuous-wave (CW) laser. In contrast, reversing this process—converting cIST back to aIST—requires an ultrafast pulsed laser, which necessitates higher power. We first deposited 80nm SiO_2 on ARM to protect it, and tested the infrared absorptance spectra, as shown in **Supplementary Fig. 34**. Then we built a laser direct writing system (**Supplementary Fig. 35**) suitable for pulsed laser (10ns).

First, we removed the objective lens and directly marking off on the sample (the power is 5.1mw), and then input an external signal to generate a single pulse on the scribing area (the power is 6.8mw). As shown in **Supplementary Fig. 37**, it can be seen that the brightness of the written area is higher than that of the non-written and erased areas. Spectra analysis reveals that the erasure process not only eliminates the shielding effect but also enhances the absorptance efficiency of the FP resonance peak in the erased region. This improvement is also attributed to diffusion during the erasure procedure. To investigate whether this improvement accumulates and affects the ability of information storage as the writing and erasing processes are repeated multiple times, we tested and compared the spectra data after one, two, and three repeats (**Supplementary Fig. 38**). Based on Kirchhoff's law and reflectivity spectra, the peak emissivity is determined to be 38.9% after one write-erase cycle, 44.6% after two cycles, and 52.8% after three cycles. The peak emissivity after the third cycle shows a 13.9% increase compared to the first cycle. This indicates that multiple write-erase cycles induce a significant change in peak emissivity. Meanwhile, the average emissivity in the 8-14 μm band is measured as 34.25%, 34.52%, and 40.94% after one, two, and three write-erase cycles, respectively. The average emissivity after the third cycle demonstrates a 6.69% increase relative to the first cycle. It can be observed that repeated cycling has a relatively minor impact on the average emissivity. We attribute this phenomenon to the fact that phase change occurs rapidly compared to interface atomic rearrangement. Even though the instantaneous temperature during amorphization may exceed 500°C, the brief heating duration (single pulse, with a pulse width < 10 ns) results in negligible interface atomic rearrangement effects.

Then we installed a 50x objective lens in the optical system, and adjusted the laser power. This process ensures localized phase change at individual points while preventing film damage by limiting power exposure to safe levels. Then, dot arrays were prepared in the aIST area and cIST area. The spectra results are shown in **Supplementary Fig. 36**. Through the analysis of the absorptance spectra, it can be found that the absorptance of the amorphous area has been greatly improved, and a new absorptance peak has been excited

between 3-5μm. The crystalline area also has an absorptance peak in this range, which is related to the plasmon resonance excited by the dot lattice structure.

Supplementary Fig. 39 compares the absorptance spectra of two processed ARM. The results revealed that laser direct writing at the same power failed to induce phase change in aIST. Instead, it augmented diffusive processes. As a result, this method can achieve decoupled modulation of diffusion and phase change in the high emissivity range. (Laser writing power: 5.1mw, heating temperature: 300℃, heating time:10min)

Supplementary Text 9

Resonance excited by high loss ENZ materials

Epsilon-near-zero (ENZ) materials commonly excite intriguing resonances, including the Berreman mode and ENZ mode. And, these resonances may induce a strong absorptance[9-12].

Interestingly, Ag-IST also exhibits ENZ characteristic at specific metallic volume filling fractions (*f*), as shown in **Supplementary Fig. 40a**. Moreover, variations in *f* could modulate the ENZ frequency. However, while Ag-IST achieves ENZ at specific *f*, the possibility of Berreman/ENZ modes' emergence still requires more investigation. In **Supplementary Fig. 40b**, we can see that the imaginary part of the permittivity are almost identical and maintain a large value with *f* varying. This makes the energy loss function almost unchanged in different *f* values and no local peaks appear (**Supplementary Fig. 40c**). From this, we can conclude that the ENZ /Berreman mode cannot be excited, the strong absorptance is induced by FP resonances (Manuscript Fig.2). To further validate it, we compared the angle reflection spectra under different *f* conditions (**Supplementary Fig. 41**) and found that they were almost no change, which once again confirmed the hypothesis.

Supplementary Text 10

Atomic migration induced metallic to dielectric transition in IST

Phase-change material IST typically requires a certain activation energy to change from the amorphous (dielectric) state to the crystalline (metallic) state, which needs overcoming an energy barrier for phase change[13-15] . Given that the crystalline state of IST possesses lower energy compared to amorphous state, reversing to the amorphous state needs melting into a disordered state and then with a cooling. Under conventional cooling rates, the material would otherwise remain in the energetically favorable crystalline state. Therefore, rapid quenching becomes essential[16]. This rapid solidification process is commonly achieved through ultrafast pulsed laser or pulsed electrical control systems[17, 18].

During atomic migration, crystalline IST exhibits a transition from metallic to dielectric characteristics (Manuscript Fig. 3). In this process, Ag atoms dope into IST. As shown in **Supplementary Fig. 42**, the formation energies of Ag atoms occupying In, Sb, and Te sites in crystalline IST are 2.42 eV, 2.61 eV, and 3.41 eV respectively[19], while the crystallization activation energy of IST stands at merely 1.8 eV[20]. The significantly higher energies indicate substantial phase separation and elemental accumulation during repeated phase change cycles. Furthermore, we characterized the XRD spectra of samples heating at different temperatures, as shown in **Supplementary Fig. 43**. Notably, the diffraction peak intensity decreased significantly when the temperature increased from 200°C to 300°C. This intensity reduction shows a disordering in the crystal structure, which can be attributed to Ag migration.

Supplementary Text 11

Ultra-broadband spectra modulation via atomic rearrangement

The atomic rearrangement proposed in this work exhibits spectra modulation that extends beyond the mid-infrared. With suitable design, its operational band can be expanded that ranging from ultraviolet to microwave. For initial verification, two structures were fabricated. Then we measured the reflectance spectra of Structure 1 in the range of 250-2500nm (**Supplementary Fig. 44**) and the S_{12} parameter of Structure 2 in the 8-12.4GHz (**Supplementary Fig. 45**). In structure 1, we got R_1 (reference), R_2 (heated at 400 °C for 10 minutes), and R_3 (preheated, then heated at 400 °C for 10 minutes). In structure 2, we got S_1 (reference) and S_2 (heated at 190°C for one hour).

Supplementary Text 12

Functional comparisons between ARM and other systems

In the field of mid-infrared radiation regulation, methodologies primarily encompass mechanical, electrical, and thermal modulation. Compared with existing approaches (**Supplementary Table 1**), this study demonstrates distinct advantages: (1) zero energy loss during operation; (2) absence of external mechanical stress; (3) ultra-wide dual-band emissivity modulation; and (4) minimal system thickness. Notably, the achieved emissivity contrast surpasses prior reports in magnitude.

Further comparisons with thermal regulation systems (**Supplementary Table 2**) highlight additional superior performance metrics: (1) exceptionally broad temperature tunability; (2) flexible temporal control; (3) diverse modulation pathways; and (4) smooth emissivity transitions without abrupt changes. In **Supplementary Table 2**, $\Delta T_{\Delta\varepsilon_{8-14}=0.01}$ represents the temperature variation required to induce a 0.01 emissivity shift at 8–14 μm wavelengths.

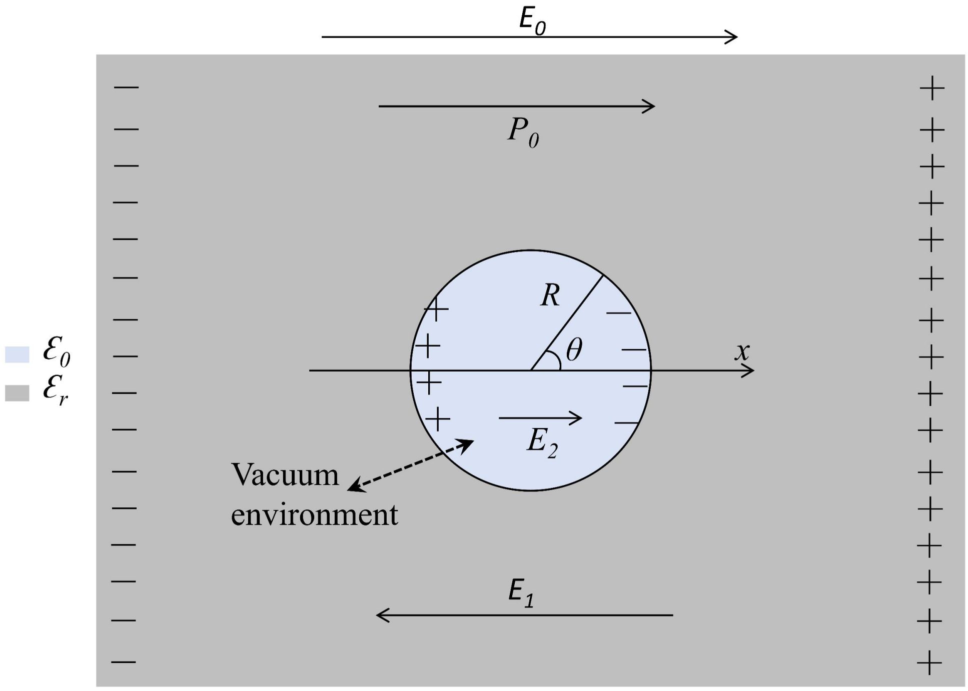

Supplementary Figure 1. Schematic of the local characteristics of the electromagnetic field in a vacuum cavity.

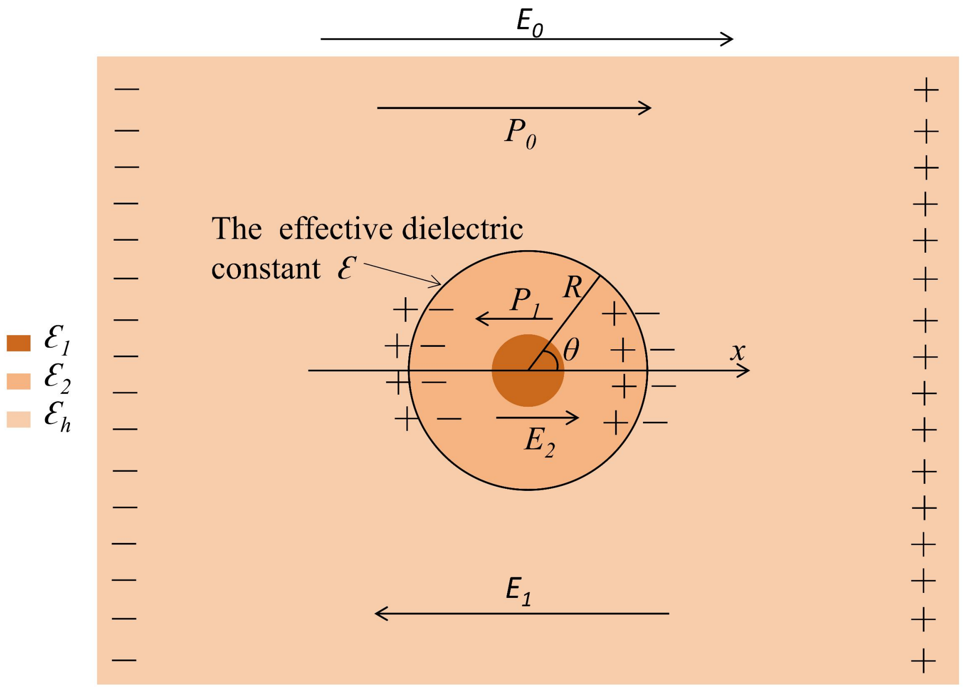

Supplementary Figure 2. Schematic of the local characteristics of the electromagnetic field in a mixed-medium cavity.

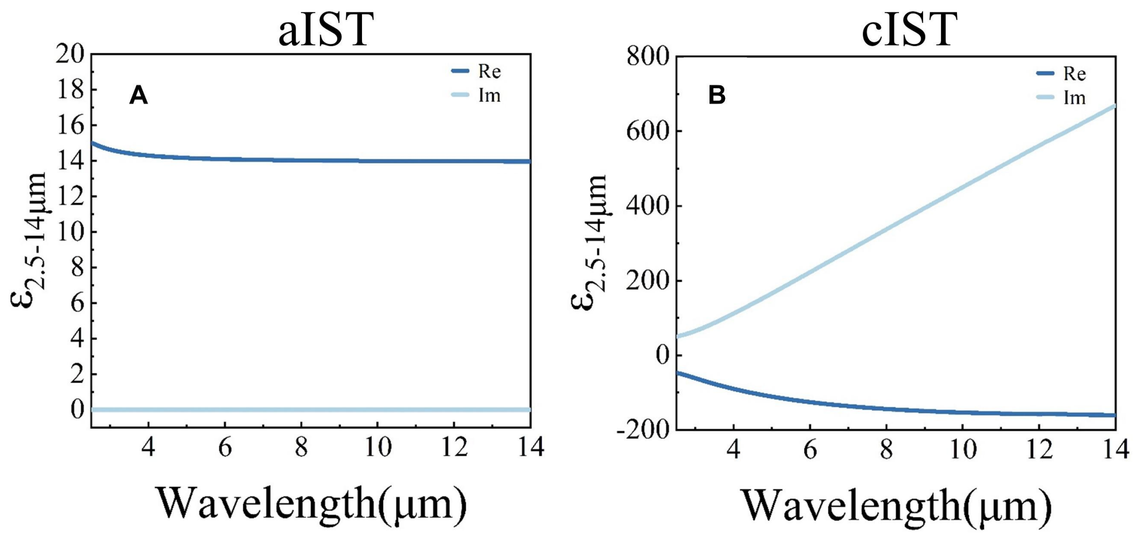

Supplementary Figure 3. Amorphous and crystalline IST permittivity. (**A**) Amorphous IST. (**B**) Crystalline IST.

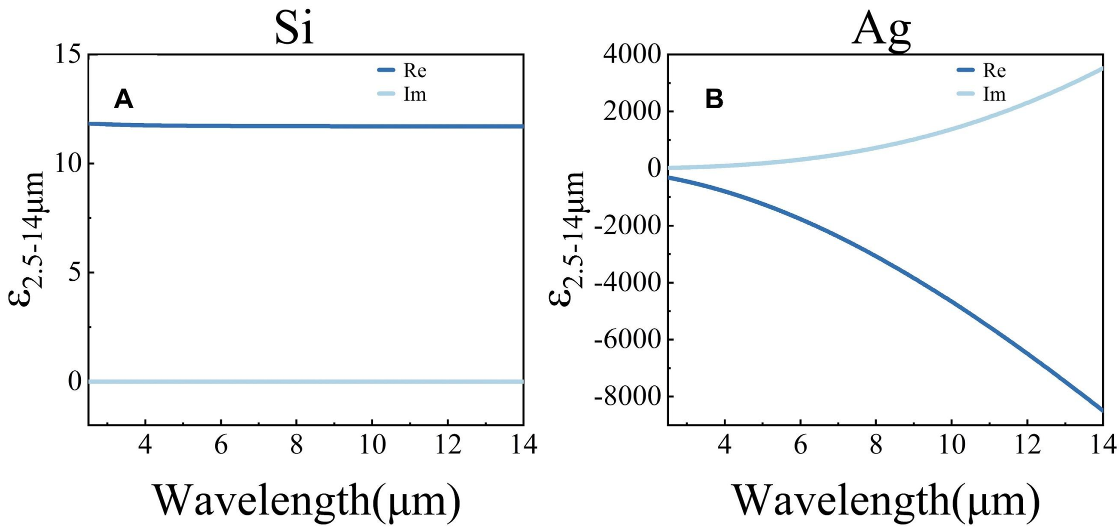

Supplementary Figure 4. Permittivity of silicon and silver. (**A**) silicon. (**B**) silver.

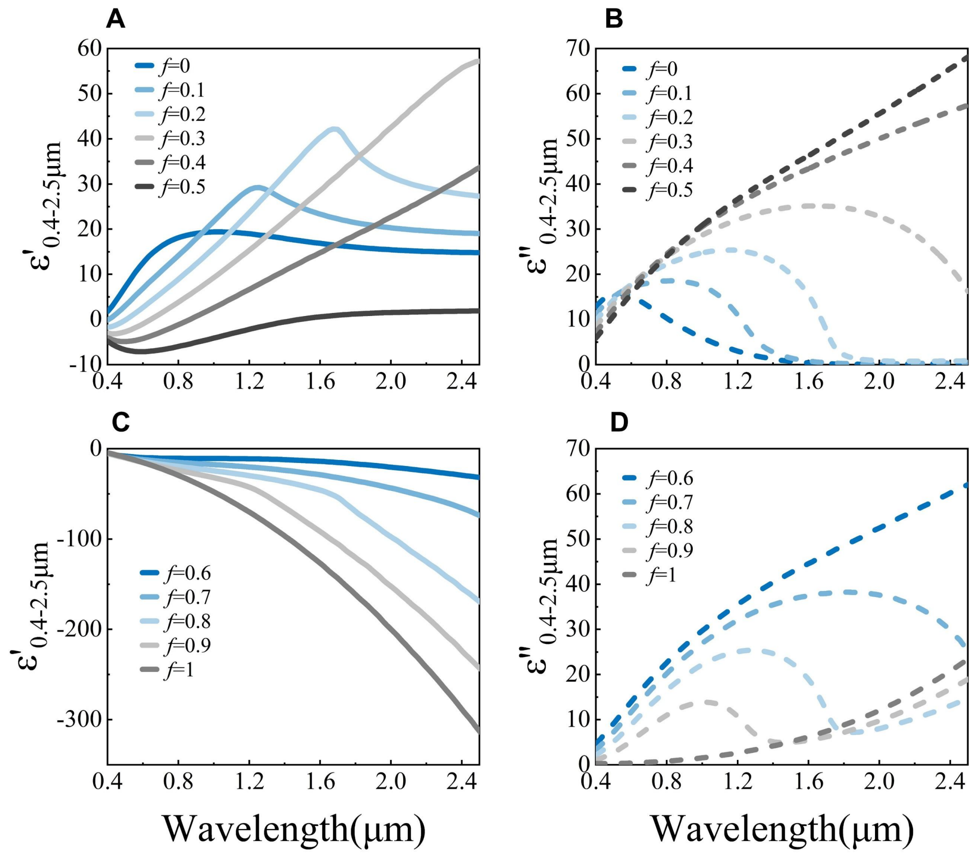

Supplementary Figure 5. Permittivity of Ag-IST (*f*: Ag volume filling fractions). (**A**) and (**C**) are the real parts. (**B**) and (**D**) are the imaginary parts.

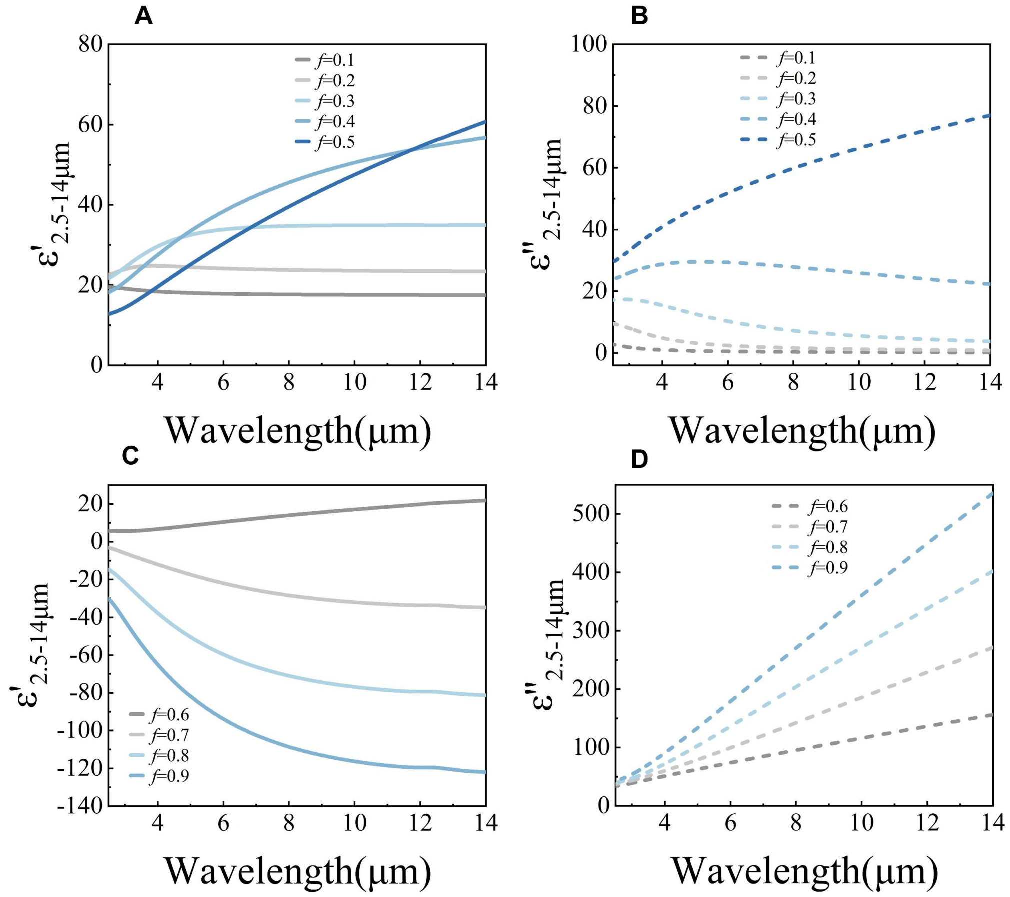

Supplementary Figure 6. Permittivity of incomplete phase change IST (*f*: cIST volume filling fractions). (**A**) and (**C**) are the real parts. (**B**) and (**D**) are the imaginary parts.

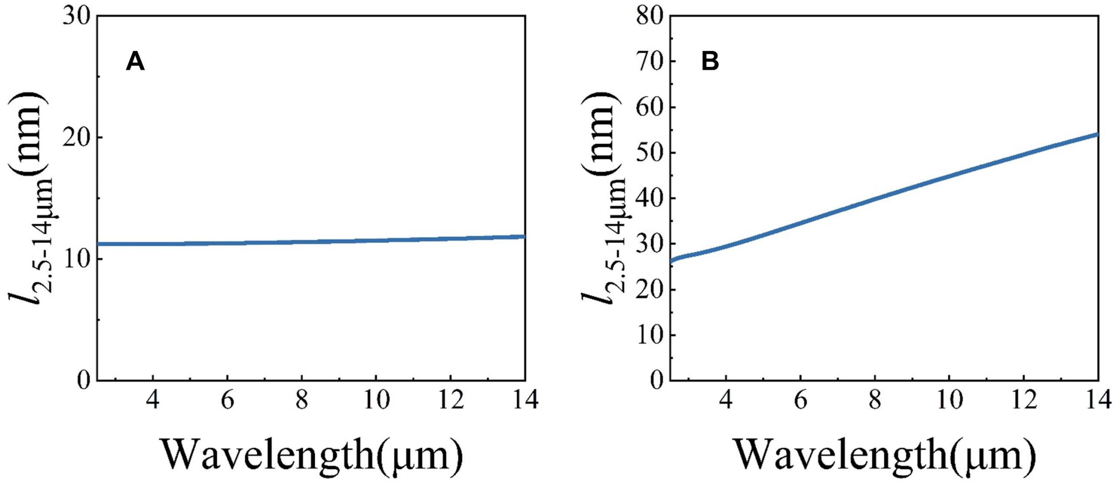

Supplementary Figure 7. Skin depth of silver and cIST. (**A**) silver. (**B**) cIST.

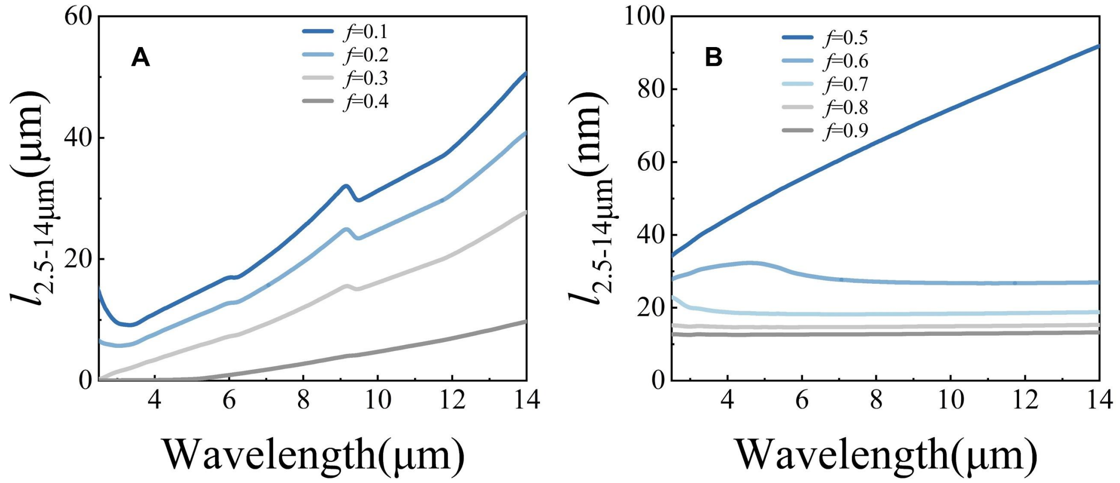

Supplementary Figure 8. Skin depth of Ag-IST with different Ag volume filling fractions *f*. (**A**) *f*=0.1-0.4. (**B**) *f*=0.5-0.9.

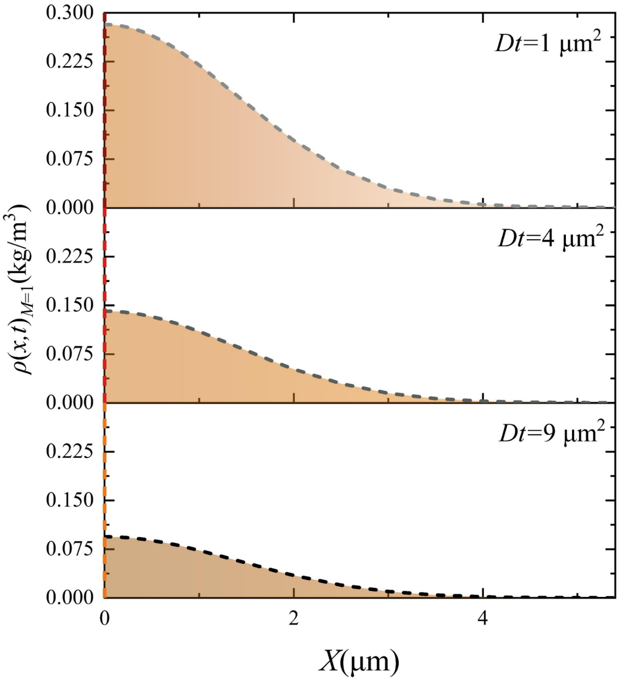

Supplementary Figure 9. Mass concentration distribution of diffusive materials at different *Dt* values with an attenuated thin film top layer.

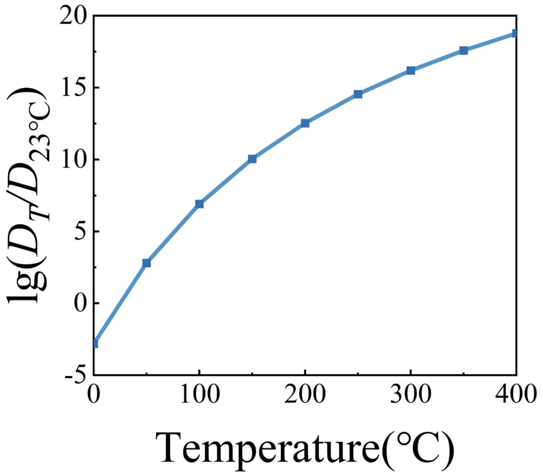

Supplementary Figure 10. Comparison of diffusion coefficients at different temperatures with that at 23℃ when both the diffusing element and the matrix metal are silver

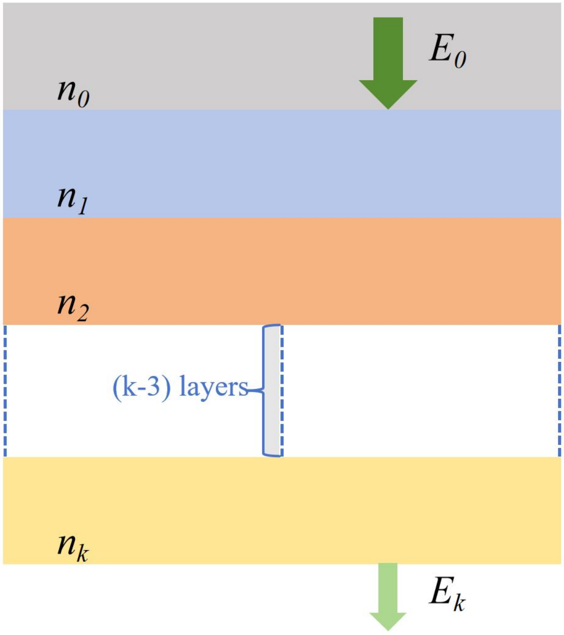

Supplementary Figure 11. Schematic diagram of the distribution of various film layers and the variation of electric fields in the calculation of the transfer matrix.

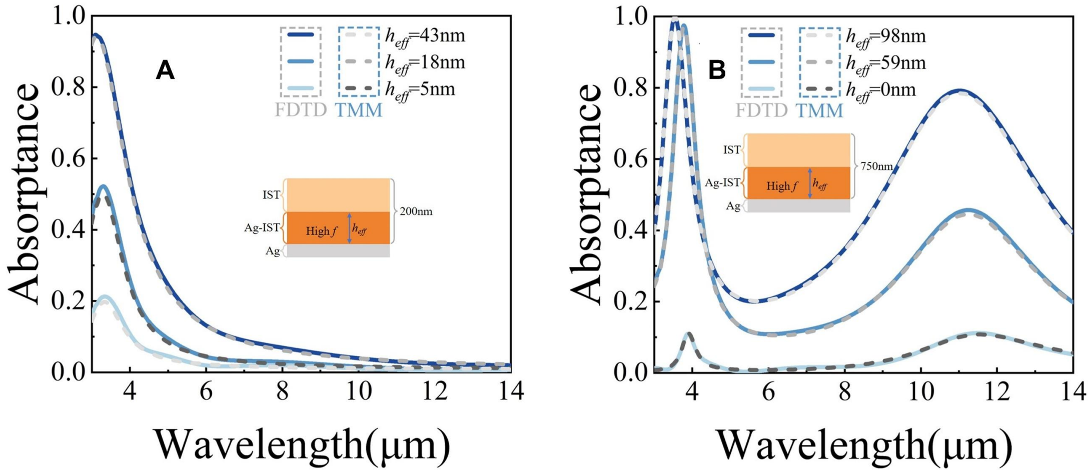

Supplementary Figure 12. Comparison of FDTD simulation results and transfer matrix calculation results. (**A**) Model 1. (**B**) Model 2.

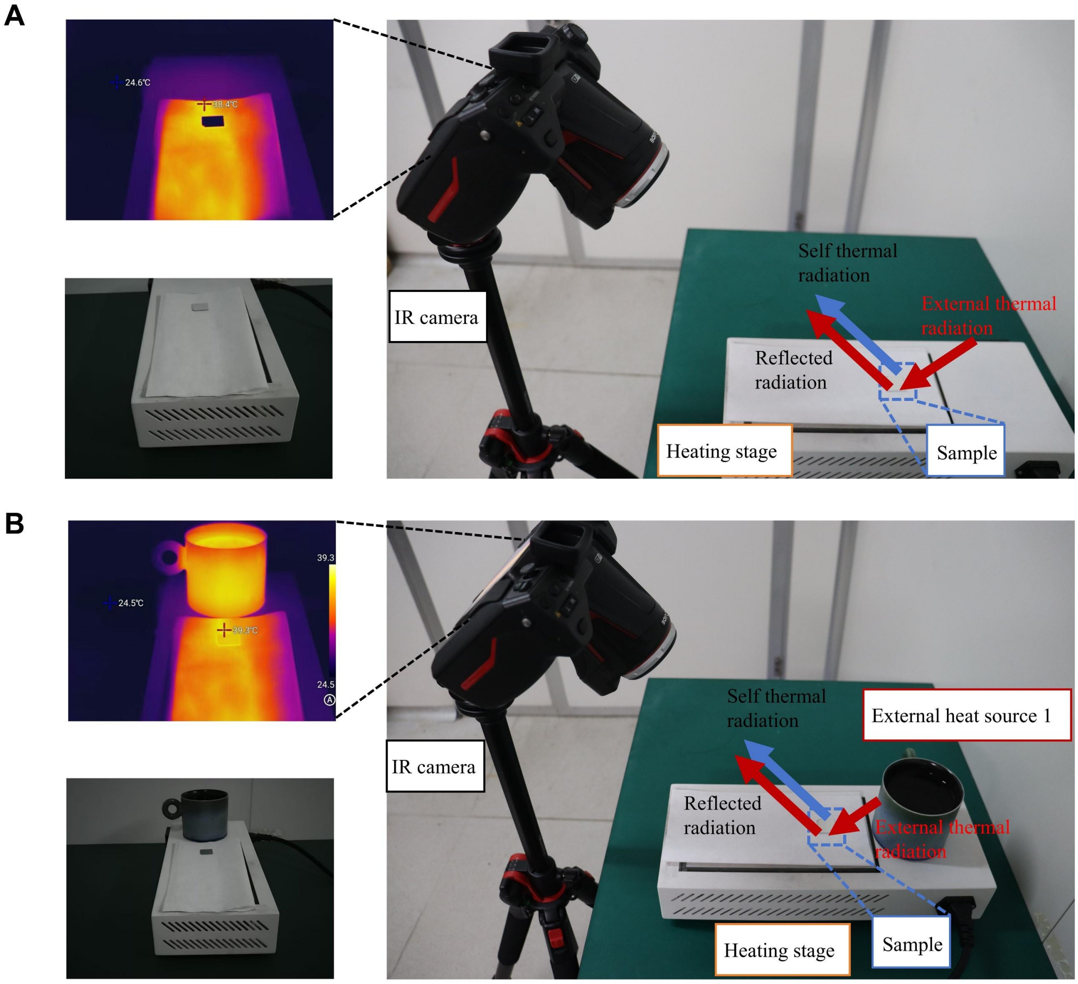

Supplementary Figure 13. Schematic diagram of the components of the thermal signal received by the infrared thermographic camera. (**A**) Without cup (low heat reflection). (**B**) With a hot cup (high heat reflection).

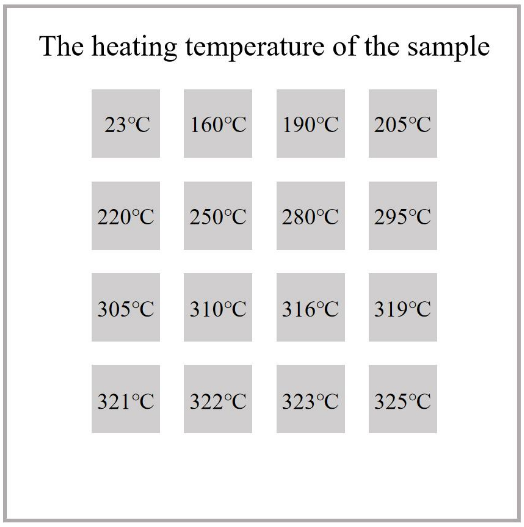

Supplementary Figure 14. The position distribution of heated samples under different temperature conditions.

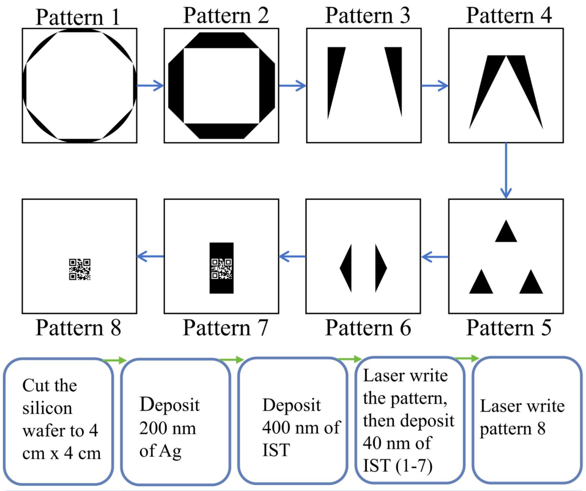

**Pattern**	1	2	3	4	5	6	7	8
**Power(mW)**	75	45	45	40	50	100	100	100

Supplementary Figure 15. Layout, processing steps, and writing power of the eight-layers system.

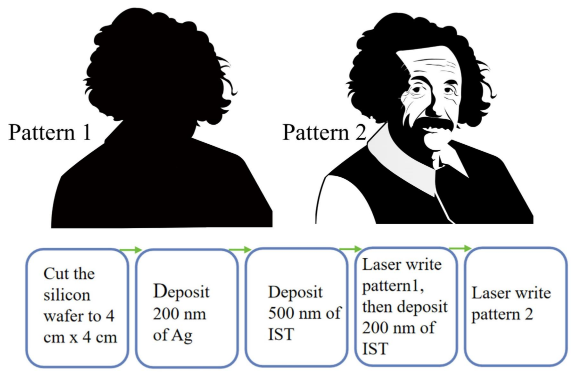

Pattern	1	2
Power(mW)	90	100

Supplementary Figure 16. Layout, processing steps, and writing power of the double-layers system with Einstein's image.

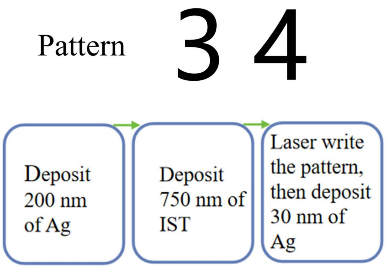

Number	3	4
Power(mW)	26.5	27.5
Counts	2	1

Supplementary Figure 17. Layout, processing steps, writing power and writing counts of the numbers 3 and 4.

Pattern 1

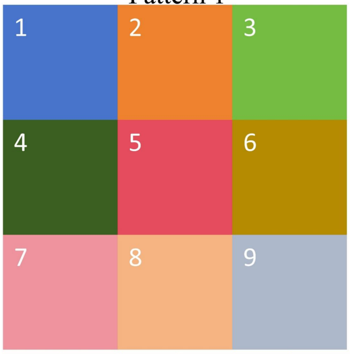

Serial number		1	2	3	4
Power		25 mW	25 mW	26.5 mW	26.5 mW
counts		2	3	1	2
Serial number	5	6	7	8	9
Power	25 mW	25 mW	27.5 mW	26.5 mW	27.5 mW
counts	1	2	1	1	2

Pattern 2

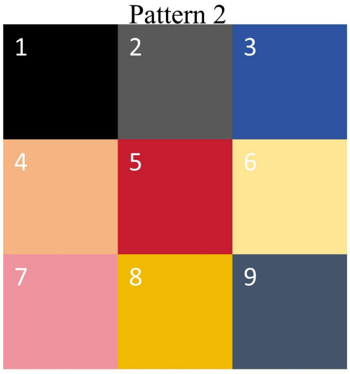

Serial number		1	2	3	4
Power		30 mW	40 mW	45 mW	50 mW
counts		1	1	1	1
Serial number	5	6	7	8	9
Power	60 mW	65 mW	70 mW	80 mW	100 mW
counts	1	1	1	1	1

Supplementary Figure 18. Layout, writing power and writing counts of the Mosaic pattern.

(1) *Written in a Village South of the Capital*

(2) *In this house on this day last year, a pink face vied*
(3) *In beauty with the pink peach blossoms side by side.*
(4) *I do not know today where the pink face has gone;*
(5) *In the vernal breeze still smile pink peach blossoms full–blown.*

Serial number	(1)	(2)	(3)	(4)	(5)
Power	28 mW	30 mW	27.5 mW	32 mW	27.5 mW
counts	2	1	1	1	3

Supplementary Figure 19. Layout, writing power and writing counts of the poem: "Written in a Village South of the Capital".

(1) 题都城南庄

(2) 去年今日此门中，
(3) 人面桃花相映红。
(4) 人面不知何处去，
(5) 桃花依旧笑春风。

Serial number	(1)	(2)	(3)	(4)	(5)
Power	50 mW	40 mW	80 mW	70 mW	60 mW
counts	1	1	1	1	1

Supplementary Figure 20. Layout, writing power and writing counts of the calligraphy style of the poem: "Written in a Village South of the Capital".

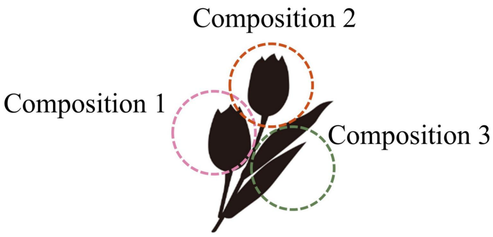

Composition	1	2	3
Power(mW)	30	35	60

Supplementary Figure 21. Layout, writing power and writing counts of the tulip pattern.

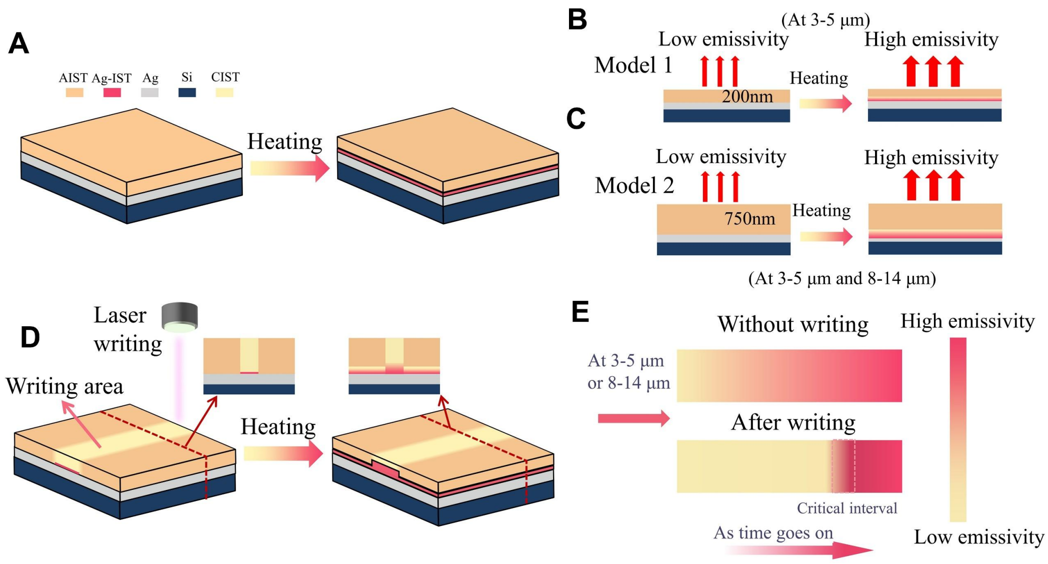

Supplementary Figure 22. Design method and composition of ARM. (**A**) 3D schematic diagram of ARM composition change when only heated. (**B**) Schematic diagram of emissivity modulation when the thickness of the aIST layer is 200nm (Model 1). (**C**) Schematic diagram of emissivity modulation when the thickness of the aIST layer is 750nm (Model 2). (**D**) Schematic diagram of composition change when heating after laser direct writing of a part of the ARM. (**E**) Schematic diagram of emissivity change of non-direct writing/direct writing area with increasing heating time.

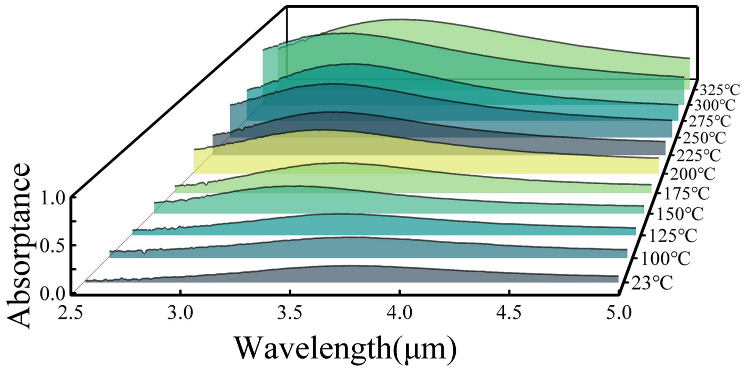

Supplementary Figure 23. Absorptance spectra of 2.5-5μm at different heating temperatures (model 1).

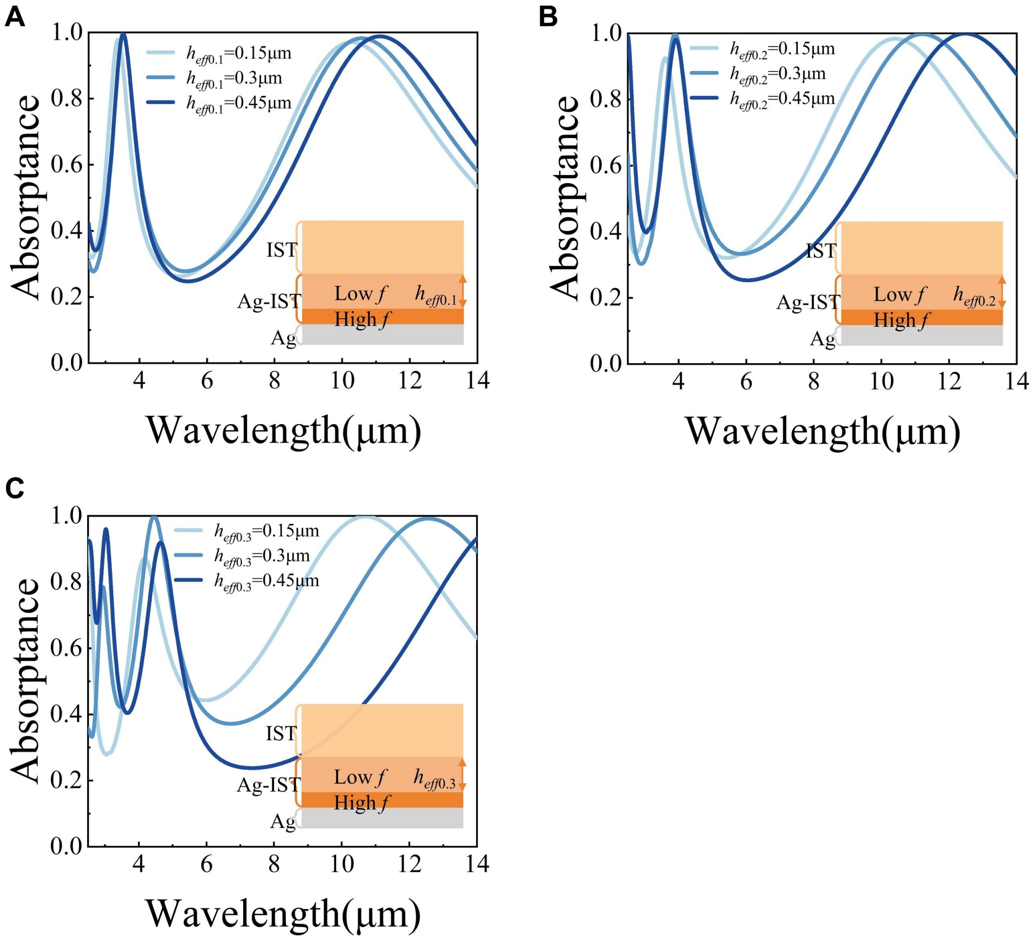

Supplementary Figure 24. Simulation of absorptance spectra for the new model (The total thickness of the Ag-IST layer and IST layer is 750nm, with the high *f* Ag-IST layer being 150nm thick). (**A**) Ag volume filling fraction *f* is 0.1 within the low volume filling fraction Ag-IST layer. (**B**) Ag volume filling fraction *f* is 0.2 within the low volume filling fraction Ag-IST layer. (**C**) Ag volume filling fraction *f* is 0.3 within the low volume filling fraction Ag-IST layer (model 2).

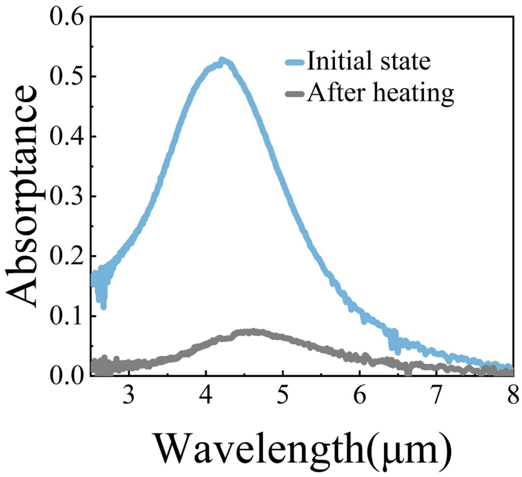

Supplementary Figure 25. Absorptance spectra of the Ag/Sb_2S_3 bilayer film device before and after heating.

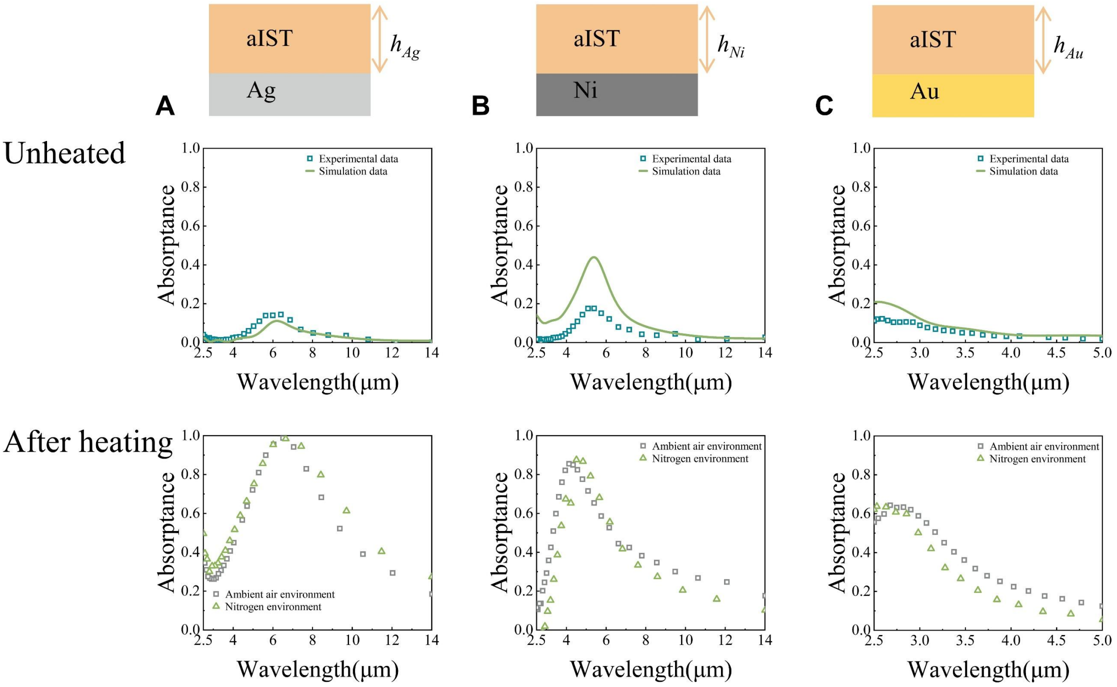

Supplementary Figure 26. Absorptance spectra of different metal substrates when not heated, heated in air, and heated in a nitrogen environment. (**A**) Silver, h_{Ag}=395nm. (**B**) Nickel, h_{Ni}=325nm. (**C**) Gold, h_{Au}=140nm.

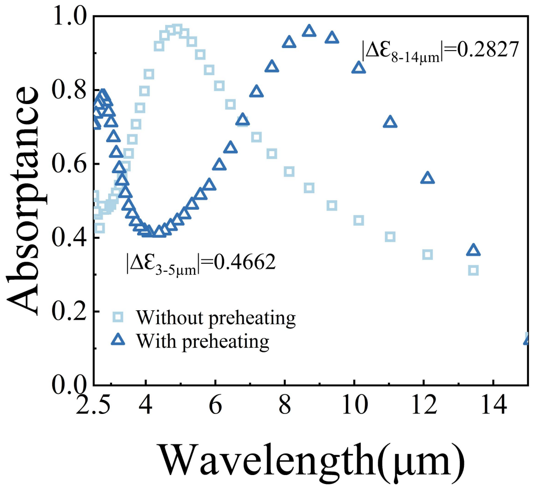

Supplementary Figure 27. Absorptance spectra of the direct-writing area of Model 2 after constant temperature heating (300℃, 6 minutes). The curves represent preheating and no preheating conditions， $\left|\Delta\varepsilon_{3\text{-}5\mu m}\right|$ is the average emissivity difference between the two conditions in the 3-5μm range. $\left|\Delta\varepsilon_{8\text{-}14\mu m}\right|$. is the average emissivity difference between the two conditions in the 8-14μm range.

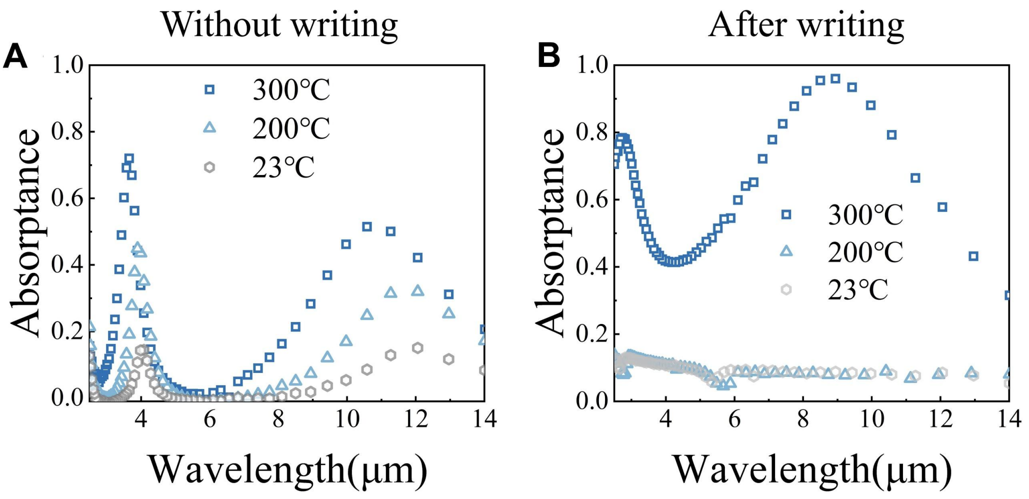

Supplementary Figure 28. Absorptance spectra of Model 2 samples after heating at different temperatures. (**A**) Without laser writing. (**B**) After laser writing.

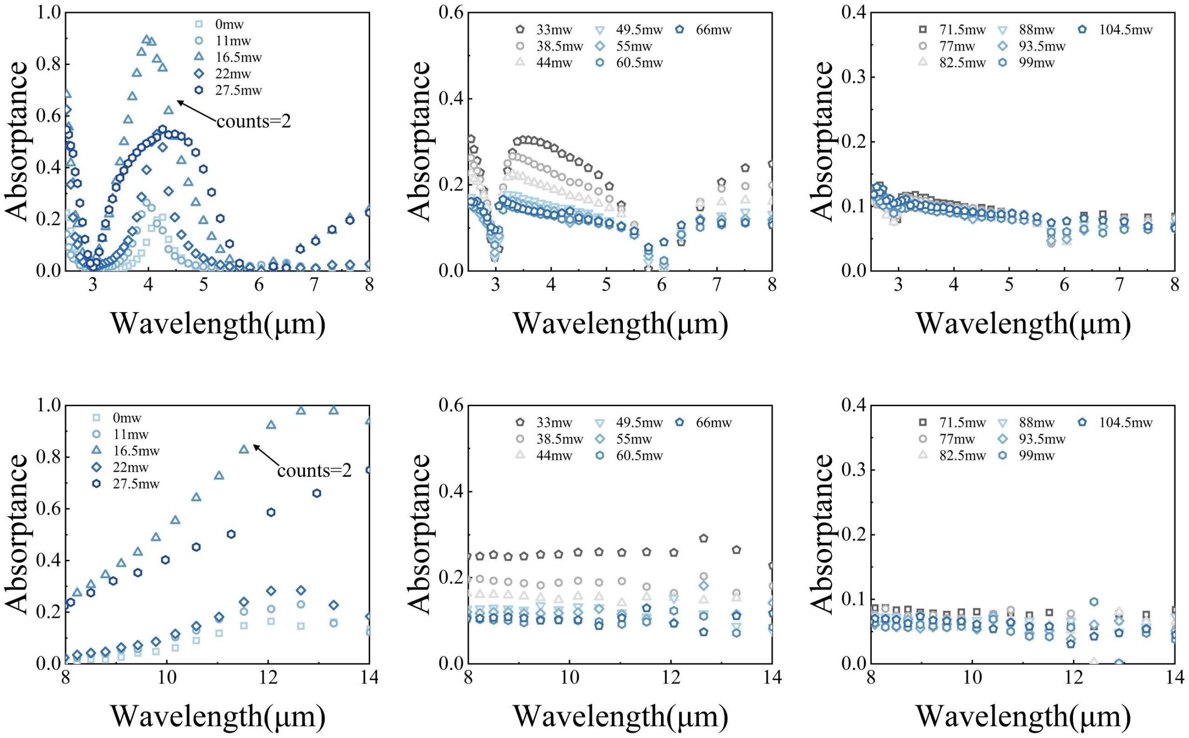

Supplementary Figure 29. Absorptance spectra of Model 2 samples after laser writing with different powers.

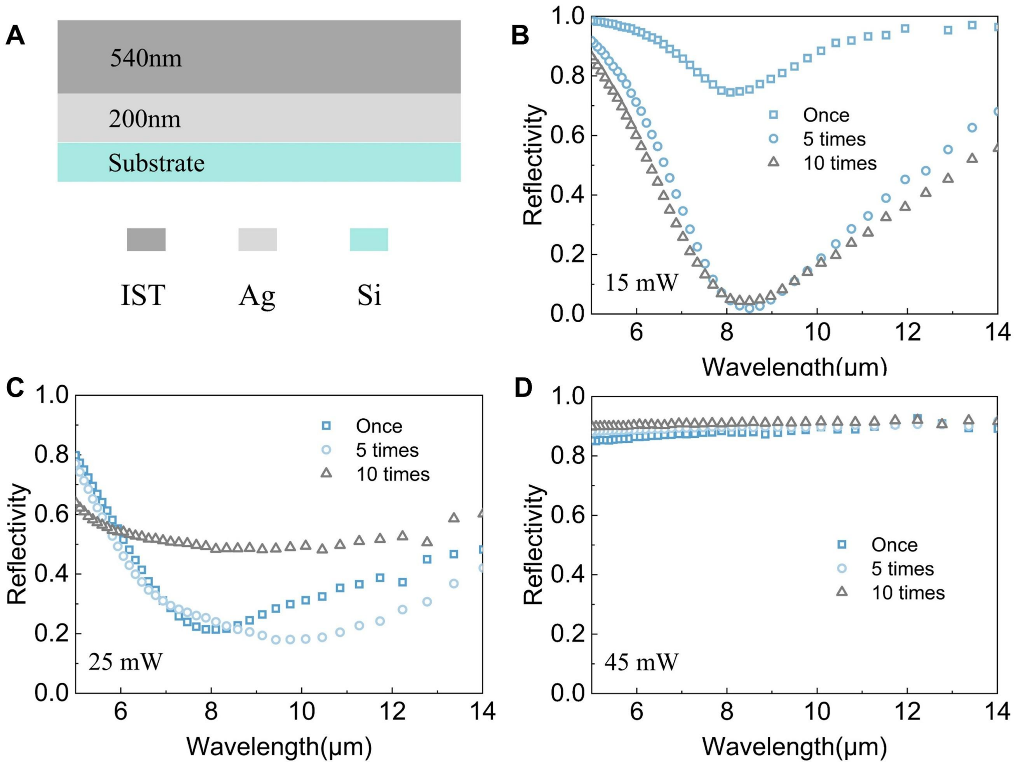

Supplementary Figure 30. The effect of multiple laser direct writing exposures under different power conditions. (A) The model. (B) 15 mW. (C) 25 mW. (D) 45 mW.

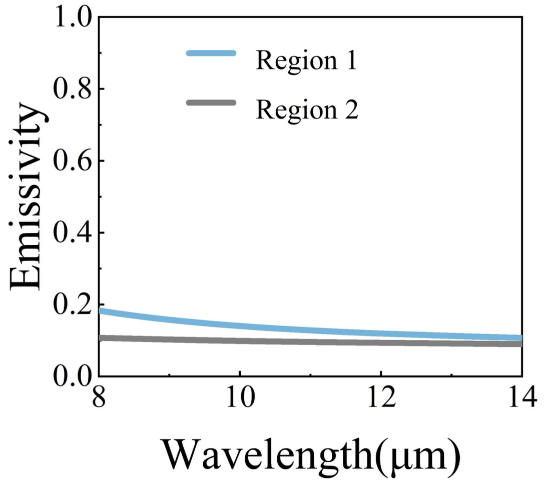

Supplementary Figure 31. Comparison of the emissivity spectra of regions 1 and 2 (shown in Fig. 5A of the manuscript).

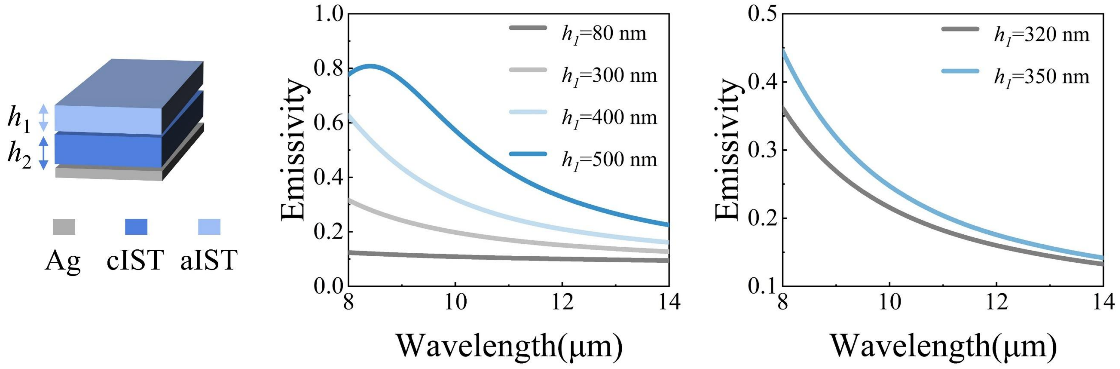

Supplementary Figure 32. Emissivity spectra for different h_1 values.

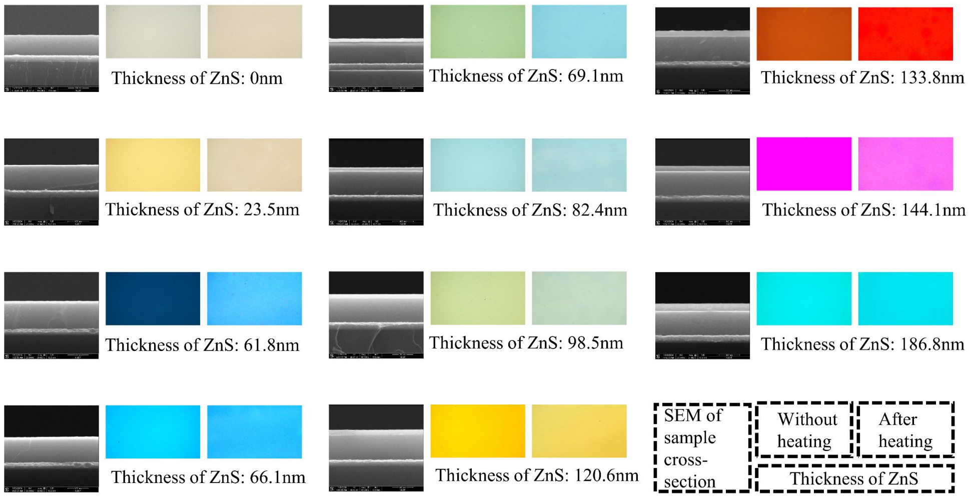

Supplementary Figure 33. The cross-sectional view and color of ARM coated with different thicknesses of ZnS. The surface color images were taken using an OLYMPUS microscopic system. The width of the unheated sample image is 330μm, and the width of the heated sample (280℃, 5 minutes) image is 165μm.

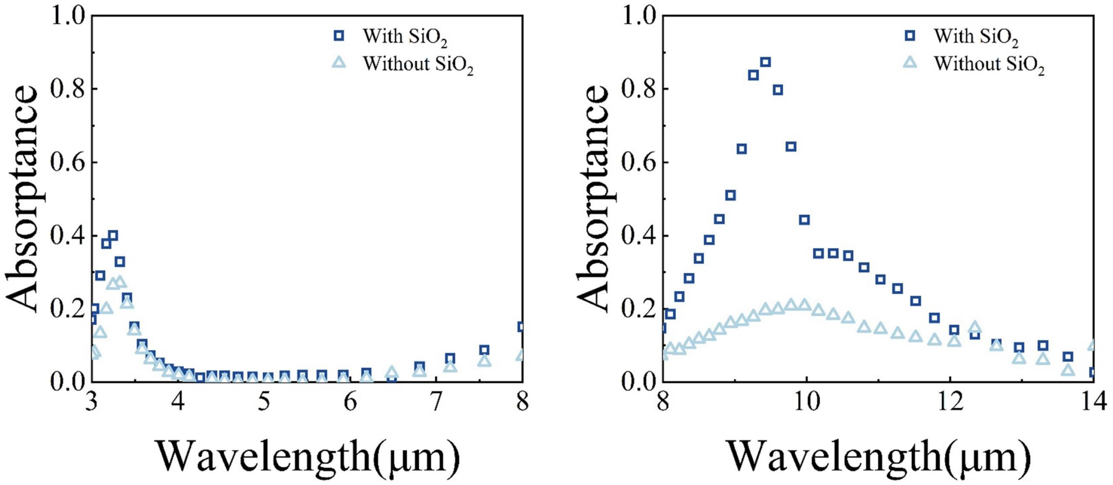

Supplementary Figure 34. Absorptance spectra of ARM coated with SiO_2(80nm), IST layer thickness is 650nm.

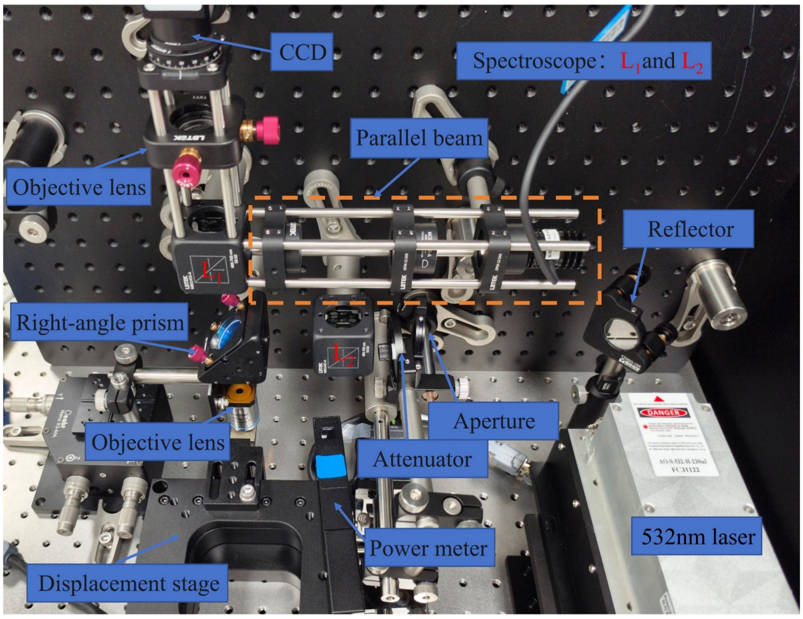

Supplementary Figure 35. Image of the laser direct writing system used for line drawing, dot marking, and erasing.

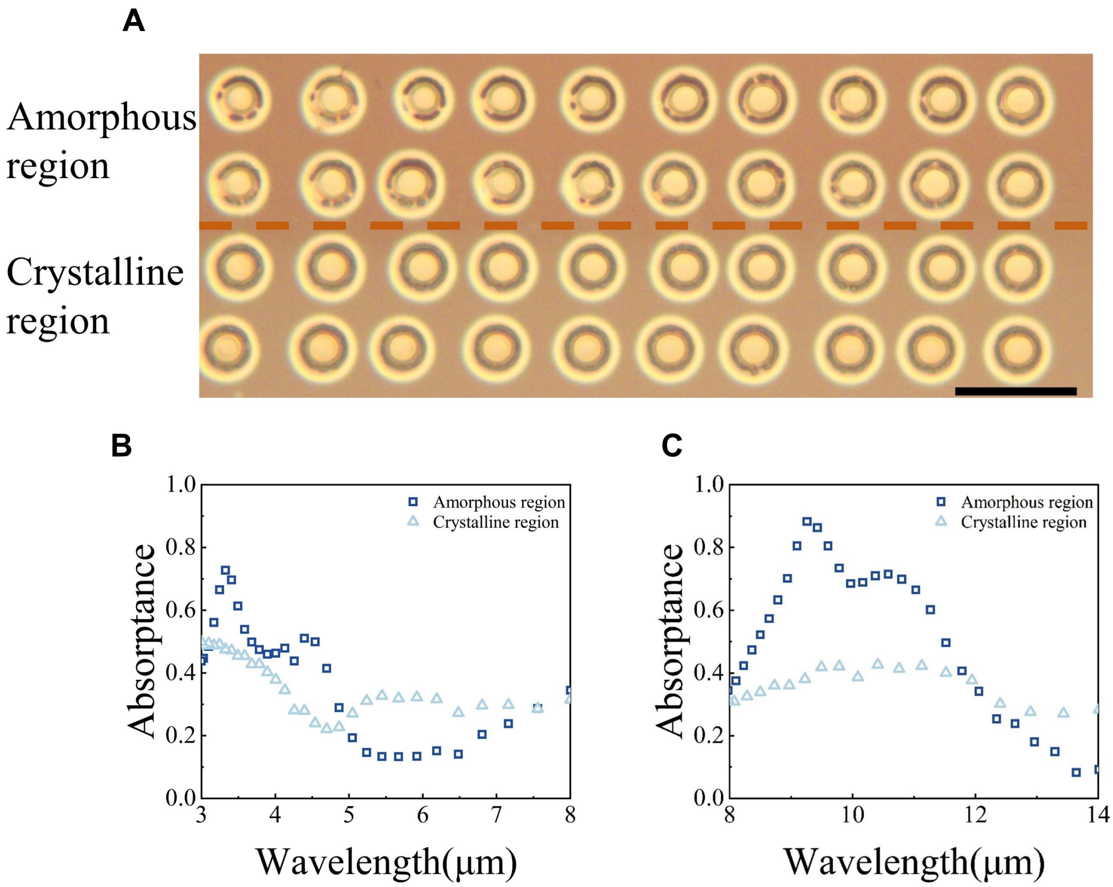

Supplementary Figure 36. Absorptance spectra and the top view of lattices in crystalline and amorphous region. (**A**) Top view of the lattice (Scale bar is 20 μm). (**B**) and (**C**) are absorptance spectra diagrams.

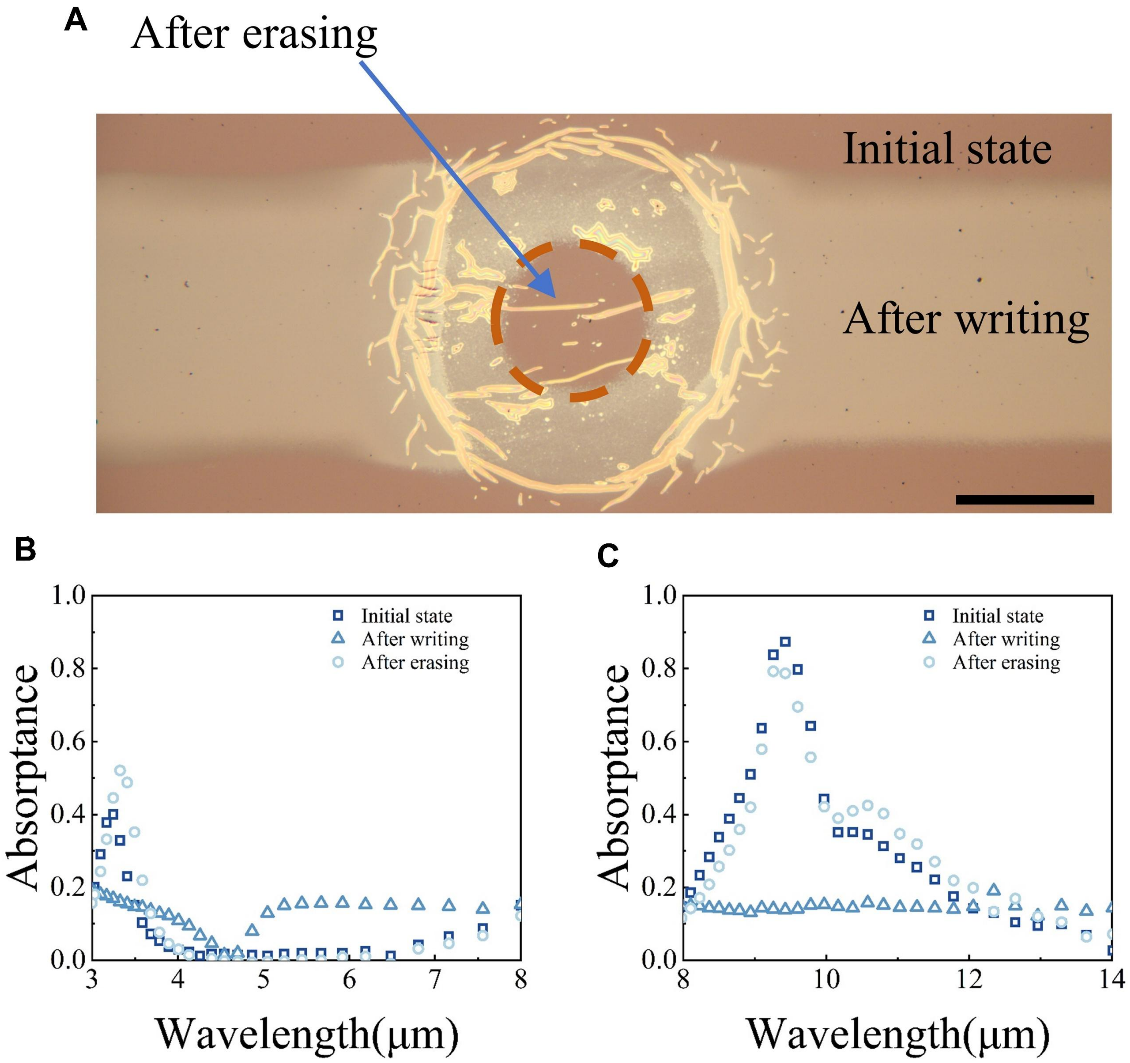

Supplementary Figure 37. Absorptance spectra and the top view of three types of regions. (**A**) Top view of three types of regions (Scale bar is 100μm). (**B**) and (**C**) are absorptance spectra diagrams.

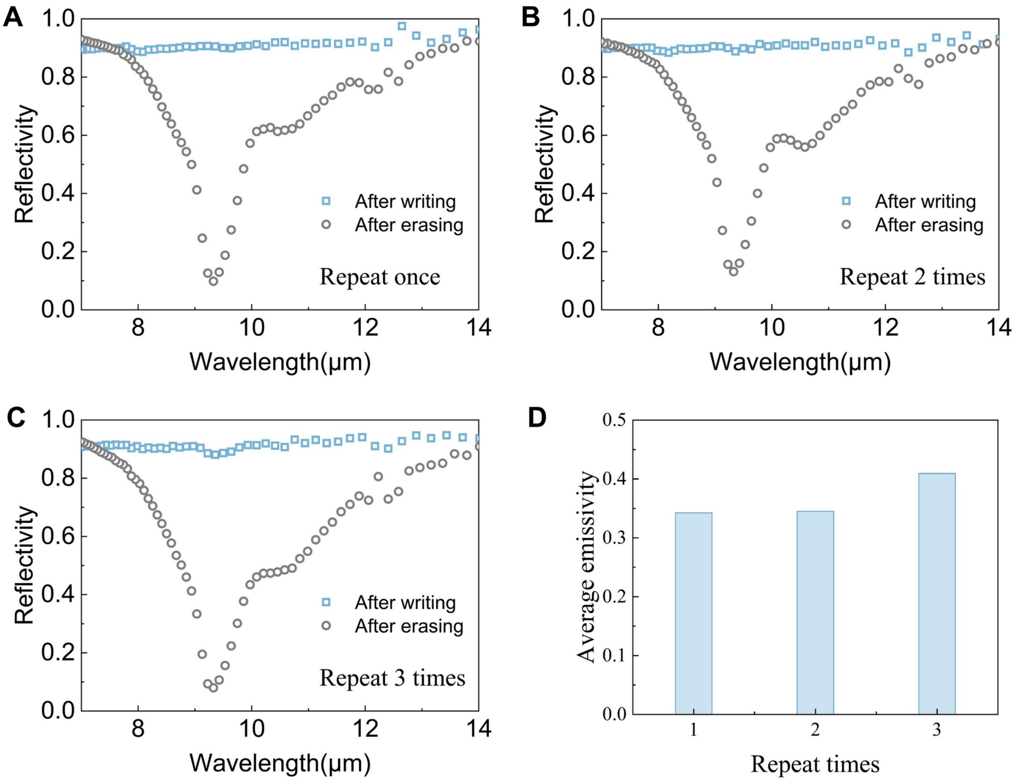

Supplementary Figure 38. Reflectivity spectra and average emissivity of the samples with different repeat times. (A) Repeat once. (B) Repeat 2 times. (C) Repeat 3 times. (D) Average emissivity.

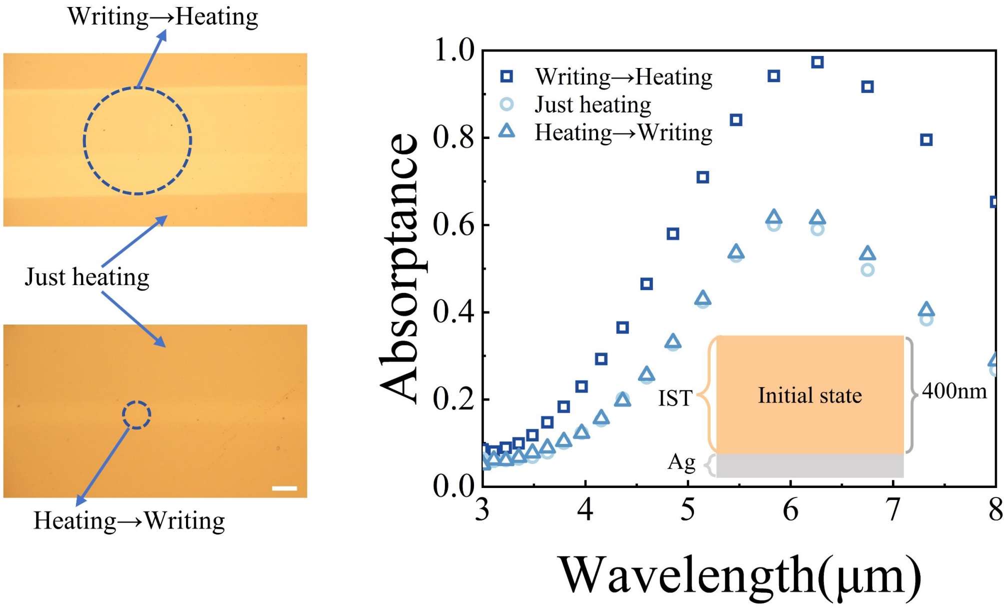

Supplementary Figure 39. Absorptance spectra and the top view of three different treated areas (Scale bar is 100μm).

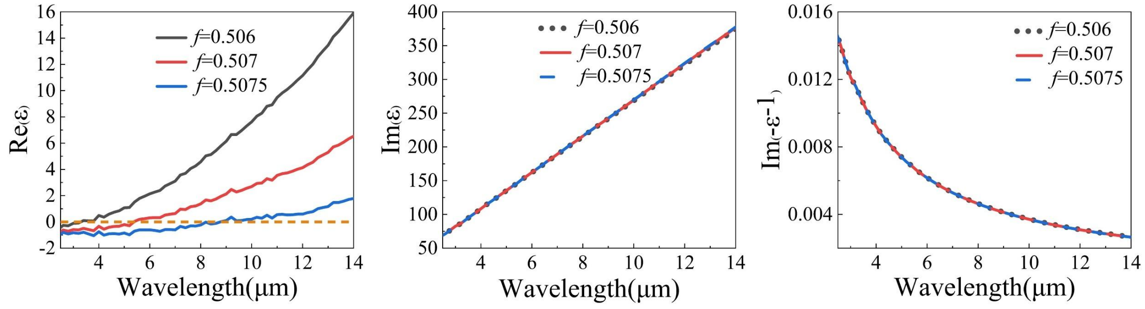

Supplementary Figure 40. Material permittivity of Ag-IST with different volume filling fractions *f*. (**A**) real part. (**B**) imaginary part. (**C**) energy-loss function.

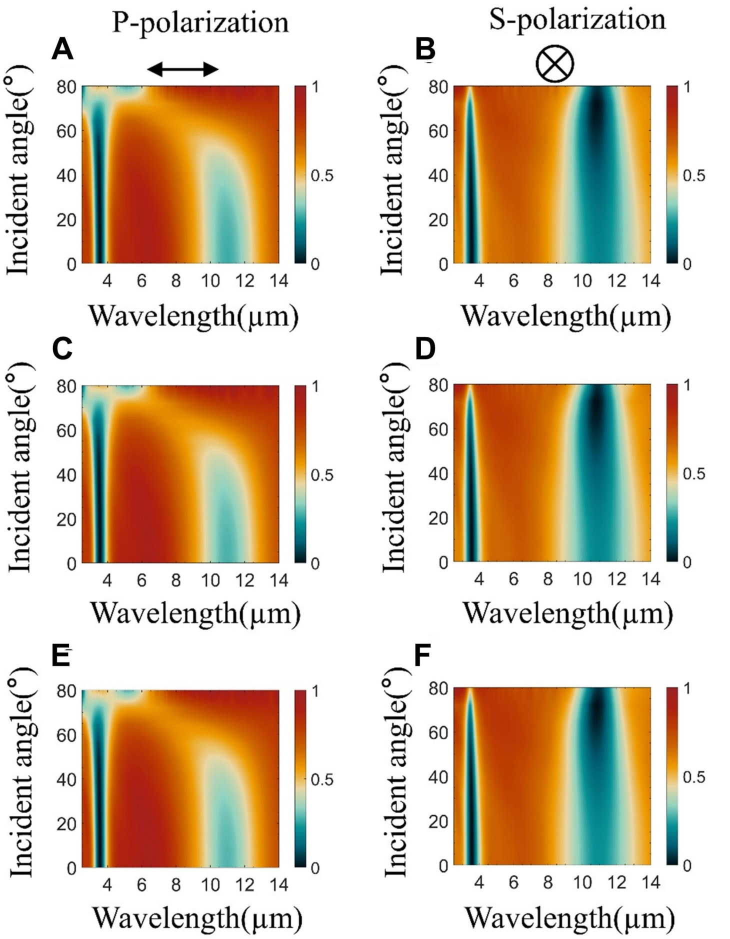

Supplementary Figure 41. Simulated reflection spectra varying with angle and wavelength in model2. (**A**) and (**B**) equivalent layer *f*=0.506, (**C**) and (**D**) equivalent layer *f*=0.507, (**E**) and (**F**) equivalent layer *f*=0.5075. Among them, incident beams in (**A**), (**C**), and (**E**) are P-polarization, while in (**B**), (**D**), and (**F**) are S-polarization.

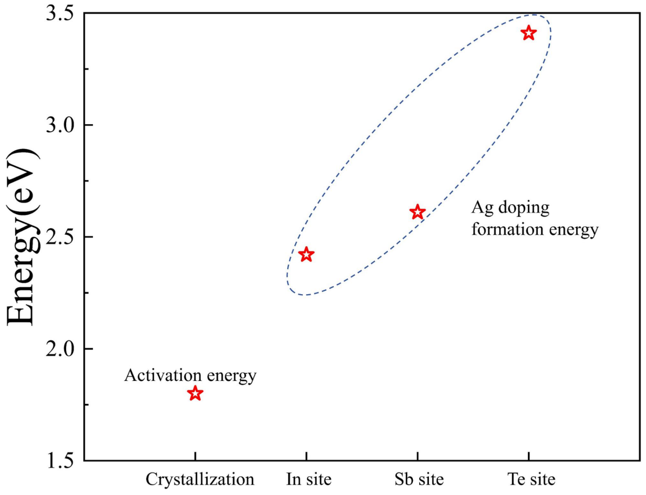

Supplementary Figure 42. The activation energy required for IST crystallization and the formation energy required for Ag to replace different sites of crystalline IST[19, 20].

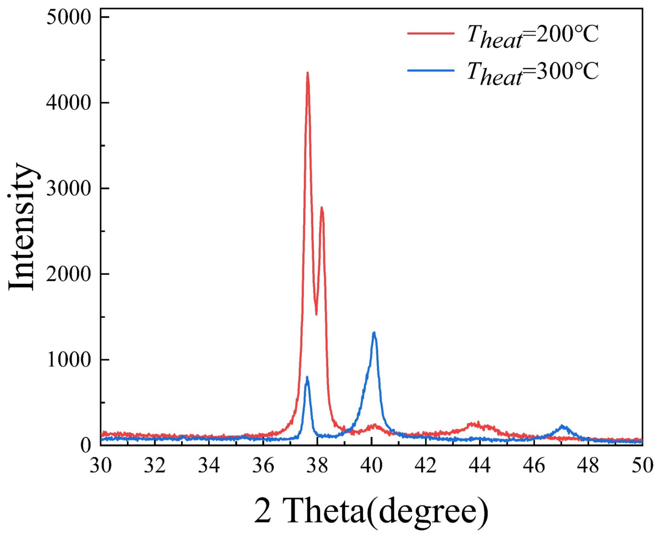

Supplementary Figure 43. XRD patterns of ARM (IST thickness : 300nm) after heating at different temperatures.

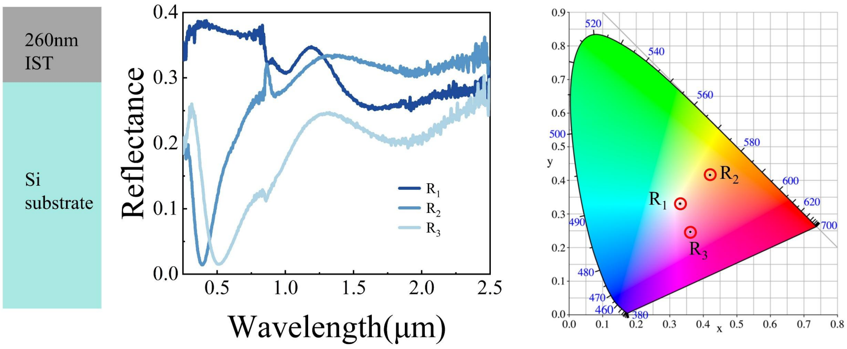

Supplementary Figure 44. Reflectance spectra and CIE 1931 chromaticity diagram of different samples.

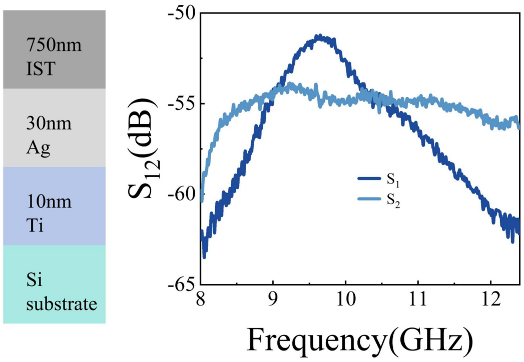

Supplementary Figure 45. S parameter (S_{12}) of different samples.

Supplementary Table 1. Comparison of IR regulators reported in previous studies

Adjustment method	Ref	Maintenance conditions	The MWIR emission rate	The LWIR emission rate	System thickness
**Mechanical control**	[21]	Mechanical stress	0.66	0.48	Thick,1mm
	[22]	Mechanical stress	Not reported	<0.448	Thick,>200μm
**Electrical control**	[23]	Electrical energy	<40%	~45%	Thick, not specified
	[24]	Electrical energy	51%	41%	Thick, not specified
**Thermal control**	[25]	Heating	40%	40%	Thick, not specified
	[26]	Heating	Not reported	70%	Thin,1.6μm
	[27]	None	Not reported	54%	Thin,525nm
	[28]	None	Not reported	~60%	Thin,570nm
	This work	None	64.74%	73.94%	Thin,950nm

Supplementary Table 2. Comparison of thermal control IR regulators reported in previous studies

**Ref**	**Materials**	**Robustness of emissivity variation**	**Effective temperature modulation range**	**Effective time modulation range**
[25]	VO_2	With a sudden change	Near 68°C	Not reported
[26]	$W_xV_{1-x}O_2$	With a sudden change	15℃-30℃	Not reported
[27]	GST	With a sudden change	130℃-160℃	Not reported
[28]	GST	With a sudden change (150°C -155°C, $\Delta T_{\Delta\varepsilon_{8\text{-}14}=0.01}\approx 0.15$℃)	140℃-170℃	1.5min while over 170℃
This work	Ag-IST	Without a sudden change (160℃-295℃ $\Delta T_{\Delta\varepsilon_{8\text{-}14}=0.01}\approx 6.21$℃)	100℃-350℃	Temperature controlled duration

Reference

1. Bruggeman, D. Calculation of various physics constants in heterogenous substances I Dielectricity constants and conductivity of mixed bodies from isotropic substances. *Annalen der Physik* **24**, 636-664 (1935).
2. Cai, W. *et al.* Optical Metamaterials. (Springer Science & Business Media, 2010).
3. Shalaev, V. M. Nonlinear optics of random media: fractal composites and metal-dielectric films. (Springer Science & Business Media, 1999).
4. Fick, A. Ueber Diffusion. *Annalen der Physik* **170**, 59-86, (2006).
5. Zhang, J., Wang, J. & Kong, D. Chloride diffusivity analysis of existing concrete based on Fick's second law. *Journal of Wuhan University of Technology-Mater. Sci. Ed.* **25**, 142-146, (2010).
6. Hu, G., Cai, X. & Rong, Y. *Fundamentals of Materials Science*, (2000).
7. Mackay, T. G. & Lakhtakia, A. The transfer-matrix method in electromagnetics and optics. (Springer Nature, 2022).
8. Snyder, W.C. *et al.* Thermodynamic constraints on reflection reciprocity and Kirchhoff's law. *Applied Optics* **37**, 3464-3470, (1998).
9. Chen, Y.-B. & Chiu, F.-C. Trapping mid-infrared rays in a lossy film with the Berreman mode, epsilon near zero mode, and magnetic polaritons. *Optics Express* **21**, (2013).
10. Ying, Y. *et al.* Whole LWIR Directional Thermal Emission Based on ENZ Thin Films. *Laser & Photonics Reviews* **16**, (2022).
11. Liu, M. *et al.* Broadband mid-infrared non-reciprocal absorption using magnetized gradient epsilon-near-zero thin films. *Nature Materials* **22**, 1196-1202, (2023).
12. Saha, S. *et al.* Engineering the temporal dynamics of all-optical switching with fast and slow materials. *Nature Communications* **14**, (2023).
13. Salinga, M. *et al.* Measurement of crystal growth velocity in a melt-quenched phase-change material. *Nature Communications* **4**, (2013).
14. Hase, M., Fons, P., Mitrofanov, K., Kolobov, A. V. & Tominaga, J. Femtosecond structural transformation of phase-change materials far from equilibrium monitored by coherent phonons. *Nature Communications* **6**, (2015).
15. Kim, D. *et al.* Phase-change mechanism and role of each element in Ag-In-Sb-Te: Chemical bond evolution. *Applied Surface Science* **544**, (2021).
16. Zhou, Y., Zhang, W., Ma, E. & Deringer, V. L. Device-scale atomistic modelling of phase-change memory materials. *Nature Electronics* **6**, 746-754, (2023).
17. Ding, J., Xu, Z., Ghosh, P. & Li, Q. A Simple Method to Reversibly Switch the Reflectance Spectra of a Layered Structure Consists of an Ultra-Thin Film Phase-Change Material GST. *Journal of Physics: Conference Series* **1838**, (2021).
18. Abdollahramezani, S. *et al.* Electrically driven reprogrammable phase-change metasurface reaching 80% efficiency. *Nature Communications* **13**, (2022).
19. Choi, M., Choi, H., Ahn, J. & Kim, Y. T. Understanding of relationship between dopant and substitutional site to develop novel phase-change materials based on In3SbTe2. *Japanese Journal of Applied Physics* **58**, (2019).

20. Maeda, Y., Andoh, H., Ikuta, I. & Minemura, H. Reversible phase-change optical data storage in InSbTe alloy films. *Journal of Applied Physics* **64**, 1715-1719, (1988).
21. Yao, B. *et al.* Cephalopod-inspired polymer composites with mechanically tunable infrared properties . *Science Bulletin* **68**, 2962-2972, (2023).
22. Zhang, Y. *et al.* Chameleon-inspired tunable multi-layered infrared-modulating system via stretchable liquid metal microdroplets in elastomer film. *Nature Communications* **15**, 5395, (2024).
23. Ergoktas, M, S. *et al.* Multispectral Graphene-Based Electro-Optical Surfaces with Reversible Tunability from Visible to Microwave Wavelengths. *Nature photonics* **15**, 493-498, (2021).
24. Jia, Y. *et al.* Transparent dynamic infrared emissivity regulators. *Nature communications* **14**, 5087, (2023).
25. Wang, S. *et al.* Scalable thermochromic smart windows with passive radiative cooling regulation. *Science* **374**, 1501-1504, (2021).
26. Tang, K. *et al.* Temperature-adaptive radiative coating for all-season household thermal regulation. *Science* **374**, 1504-1509, (2021).
27. Kim, Y., Kim, C. & Lee, M. Parallel Laser Printing of a Thermal Emission Pattern in a Phase-Change Thin Film Cavity for Infrared Camouflage and Security. *Laser and Photonics Reviews*. (2021).
28. Du, K. *et al.* Control over emissivity of zero-static-power thermal emitters based on phase-changing material GST. *Light: Science & Applications* **6**, e16194, (2016).
