# Supplementary figures and images for: Programmable Interface Atomic Rearrangement for Spatiotemporal Thermal Radiation Tailoring
Source: Research (Wash D C). 2026 Mar 6;9:1141. doi: 10.34133/research.1141 (PMC12963646; doi:10.34133/research.1141)

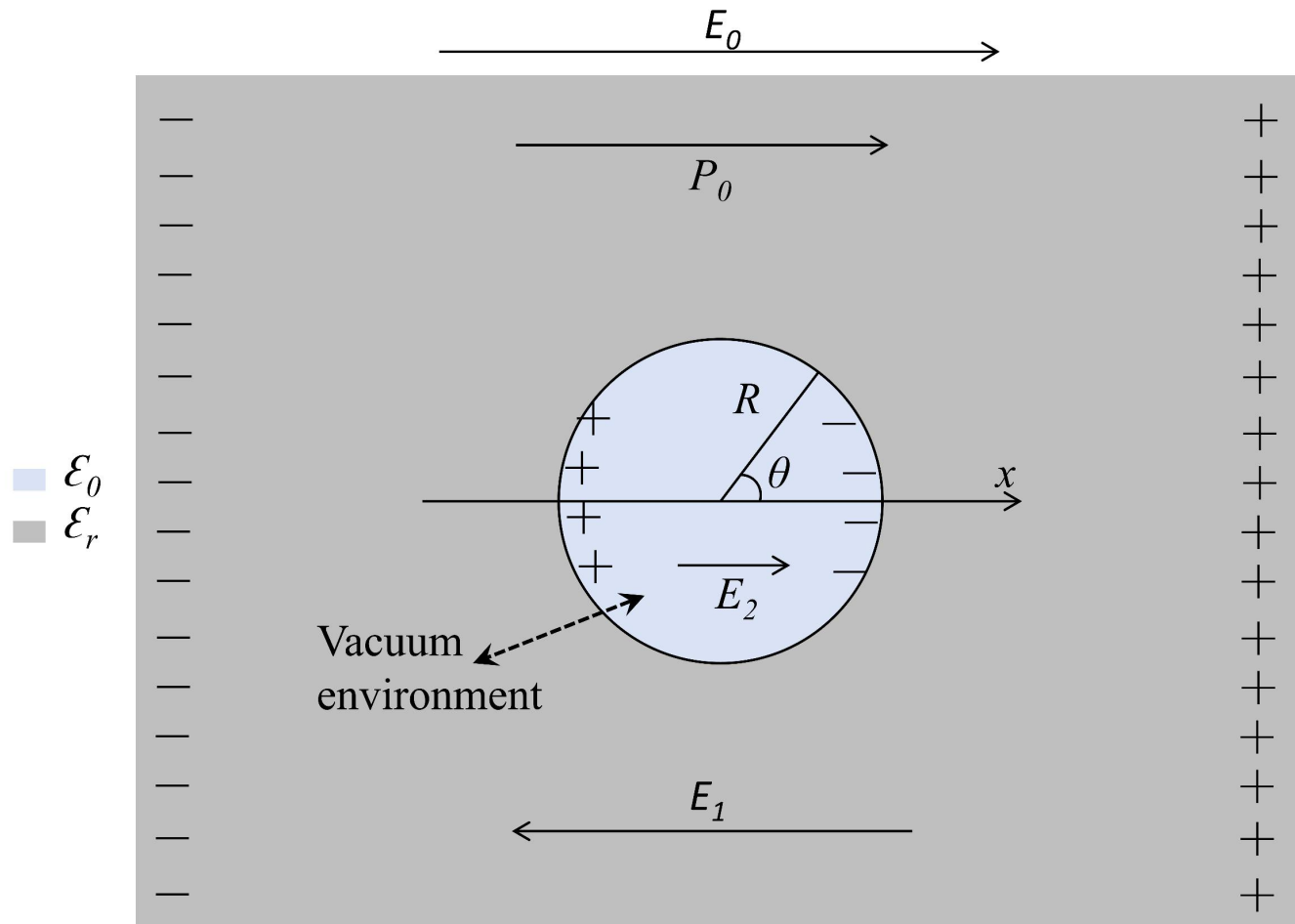

Supplement: Supplementary 1 — Texts S1 to S12 Figs. S1 to S45 Tables S1 and S2 Movies S1 to S3 [file research.1141.f1.zip › S1.pdf]

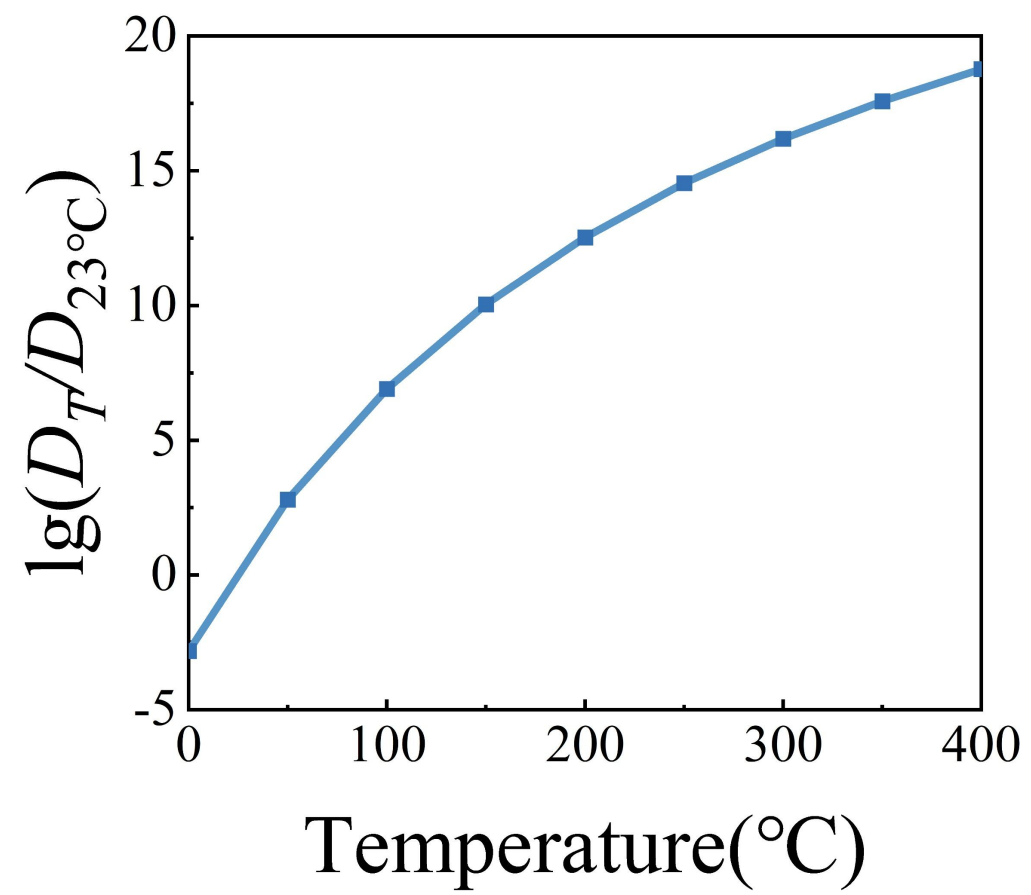

Supplement: Supplementary 1 — Texts S1 to S12 Figs. S1 to S45 Tables S1 and S2 Movies S1 to S3 [file research.1141.f1.zip › S10.pdf]

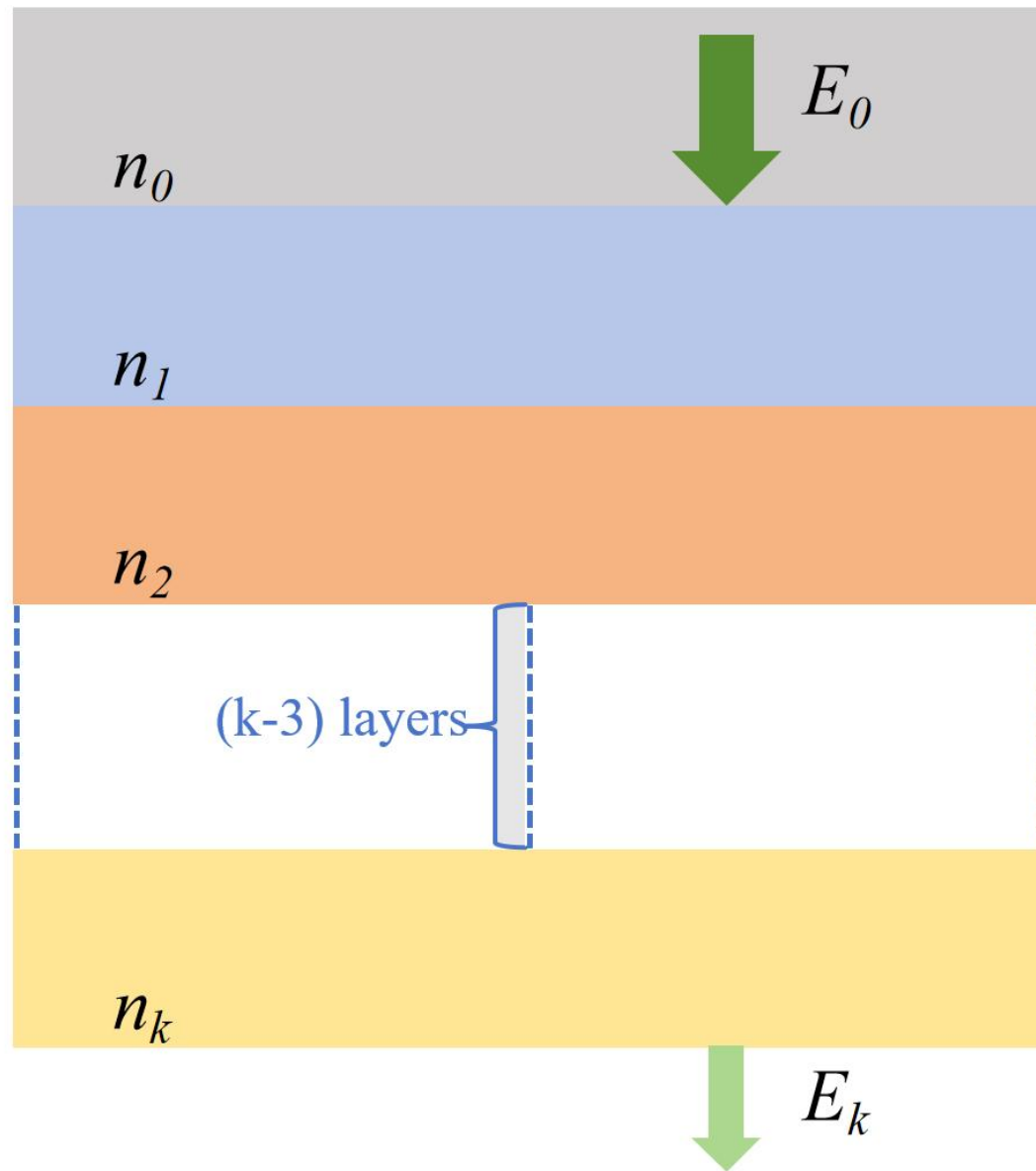

Supplement: Supplementary 1 — Texts S1 to S12 Figs. S1 to S45 Tables S1 and S2 Movies S1 to S3 [file research.1141.f1.zip › S11.pdf]

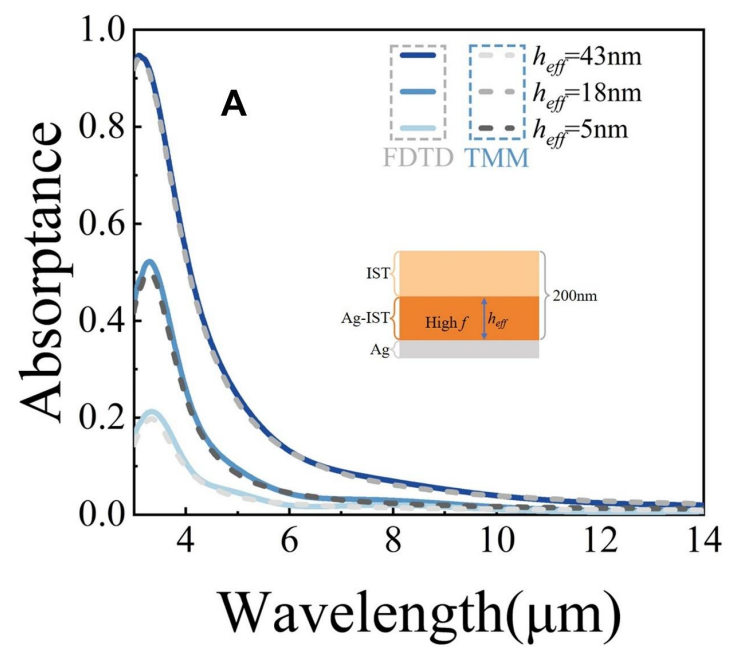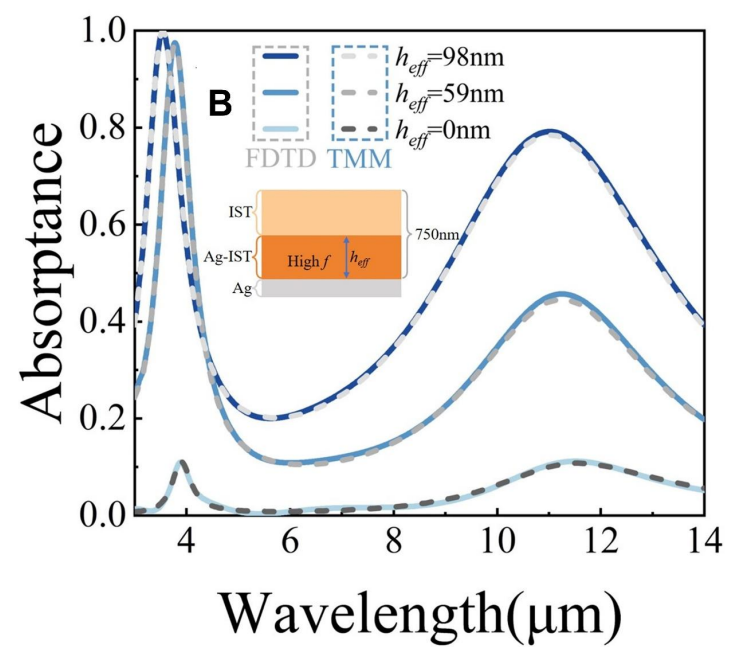

Supplement: Supplementary 1 — Texts S1 to S12 Figs. S1 to S45 Tables S1 and S2 Movies S1 to S3 [file research.1141.f1.zip › S12.pdf]

**A**

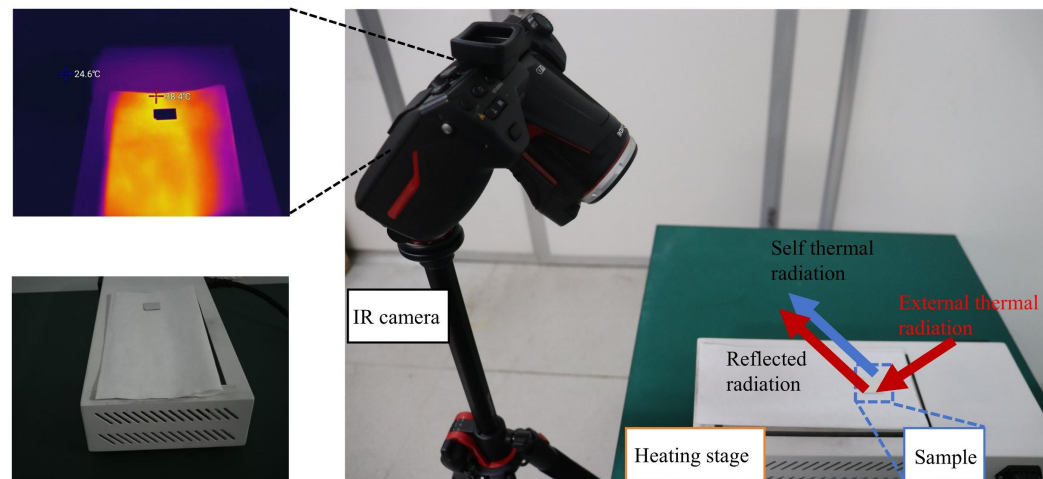

**B**

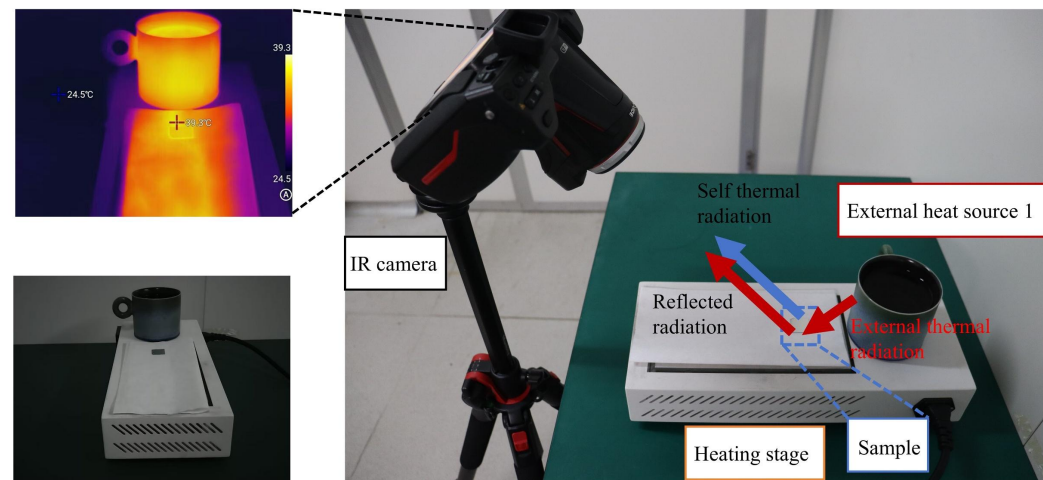

Supplement: Supplementary 1 — Texts S1 to S12 Figs. S1 to S45 Tables S1 and S2 Movies S1 to S3 [file research.1141.f1.zip › S13.pdf]

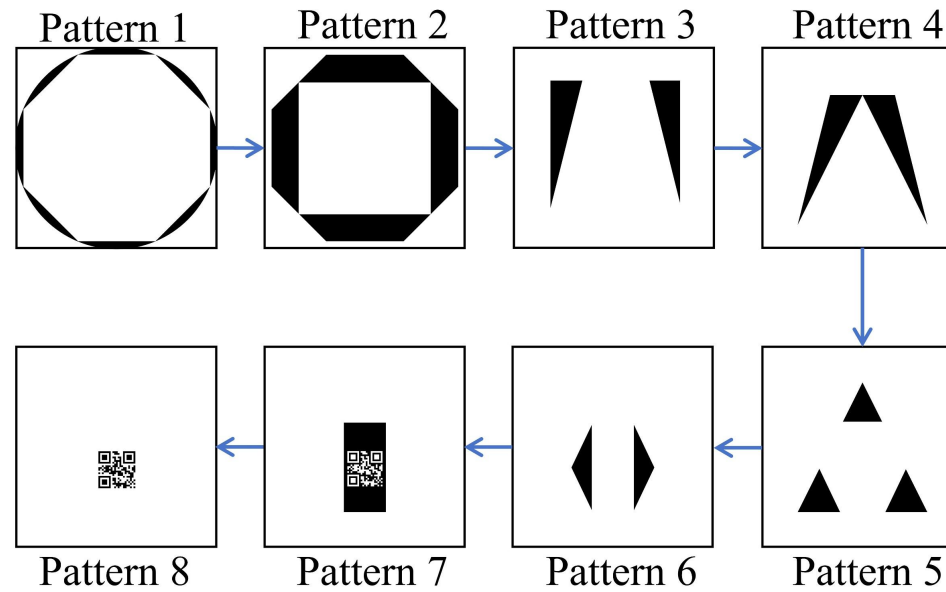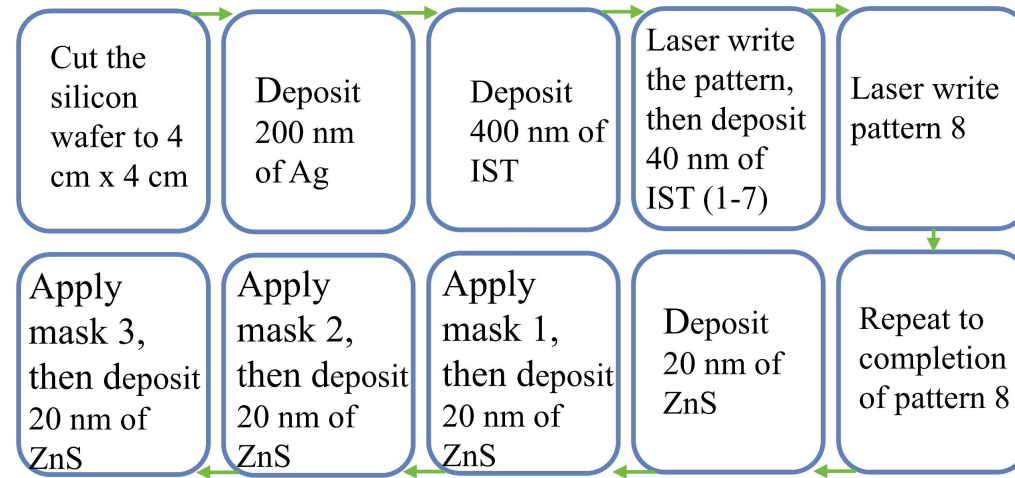

Supplement: Supplementary 1 — Texts S1 to S12 Figs. S1 to S45 Tables S1 and S2 Movies S1 to S3 [file research.1141.f1.zip › S15.pdf]

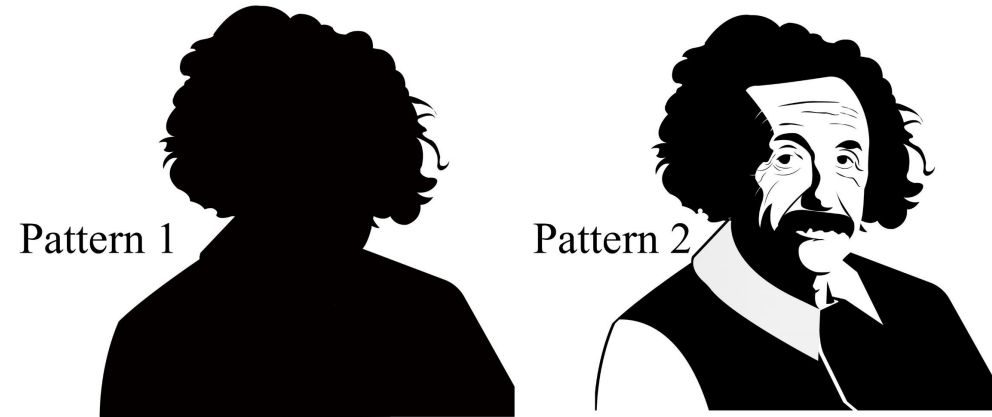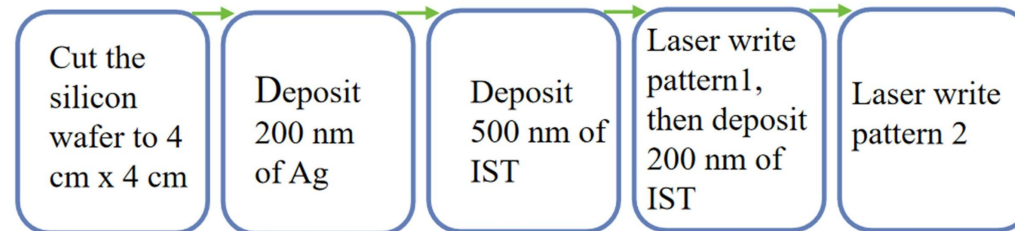

| Pattern   | 1  | 2   |
|-----------|----|-----|
| Power(mW) | 90 | 100 |

Supplement: Supplementary 1 — Texts S1 to S12 Figs. S1 to S45 Tables S1 and S2 Movies S1 to S3 [file research.1141.f1.zip › S16.pdf]

# Pattern 3 4

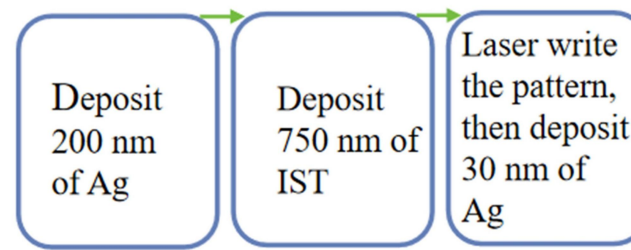

| Number    | 3    | 4    |
|-----------|------|------|
| Power(mW) | 26.5 | 27.5 |
| Counts    | 2    | 1    |

Supplement: Supplementary 1 — Texts S1 to S12 Figs. S1 to S45 Tables S1 and S2 Movies S1 to S3 [file research.1141.f1.zip › S17.pdf]

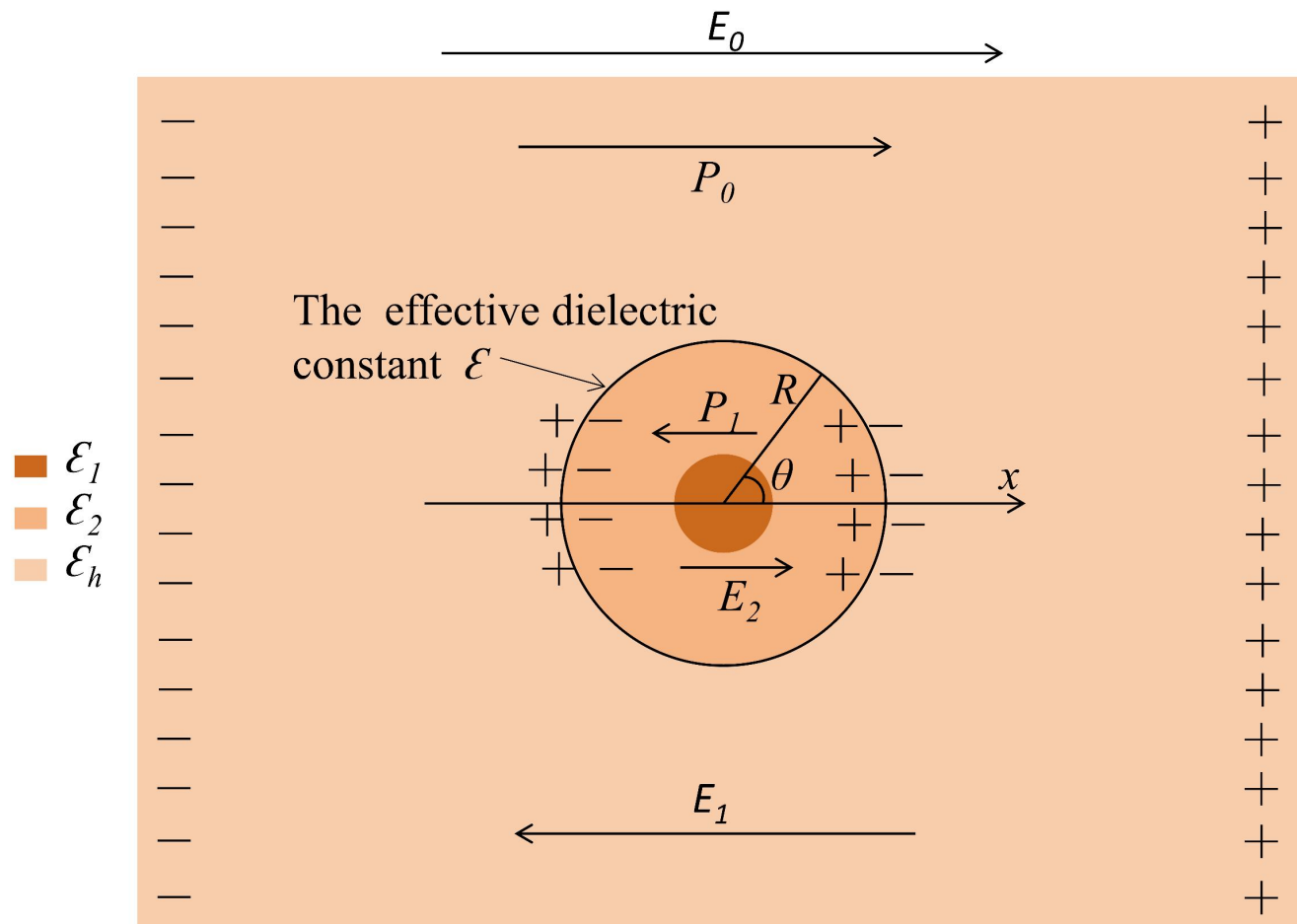

Supplement: Supplementary 1 — Texts S1 to S12 Figs. S1 to S45 Tables S1 and S2 Movies S1 to S3 [file research.1141.f1.zip › S2.pdf]

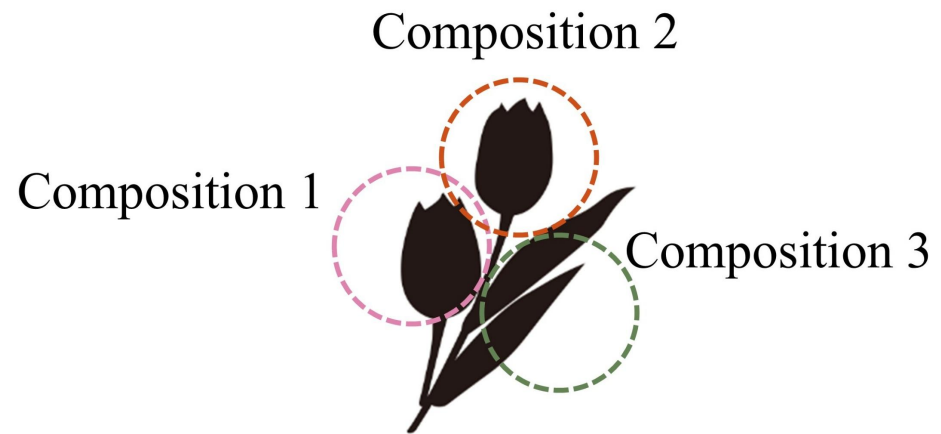

| Composition | 1  | 2  | 3  |
|-------------|----|----|----|
| Power(mW)   | 30 | 35 | 60 |

Supplement: Supplementary 1 — Texts S1 to S12 Figs. S1 to S45 Tables S1 and S2 Movies S1 to S3 [file research.1141.f1.zip › S21.pdf]

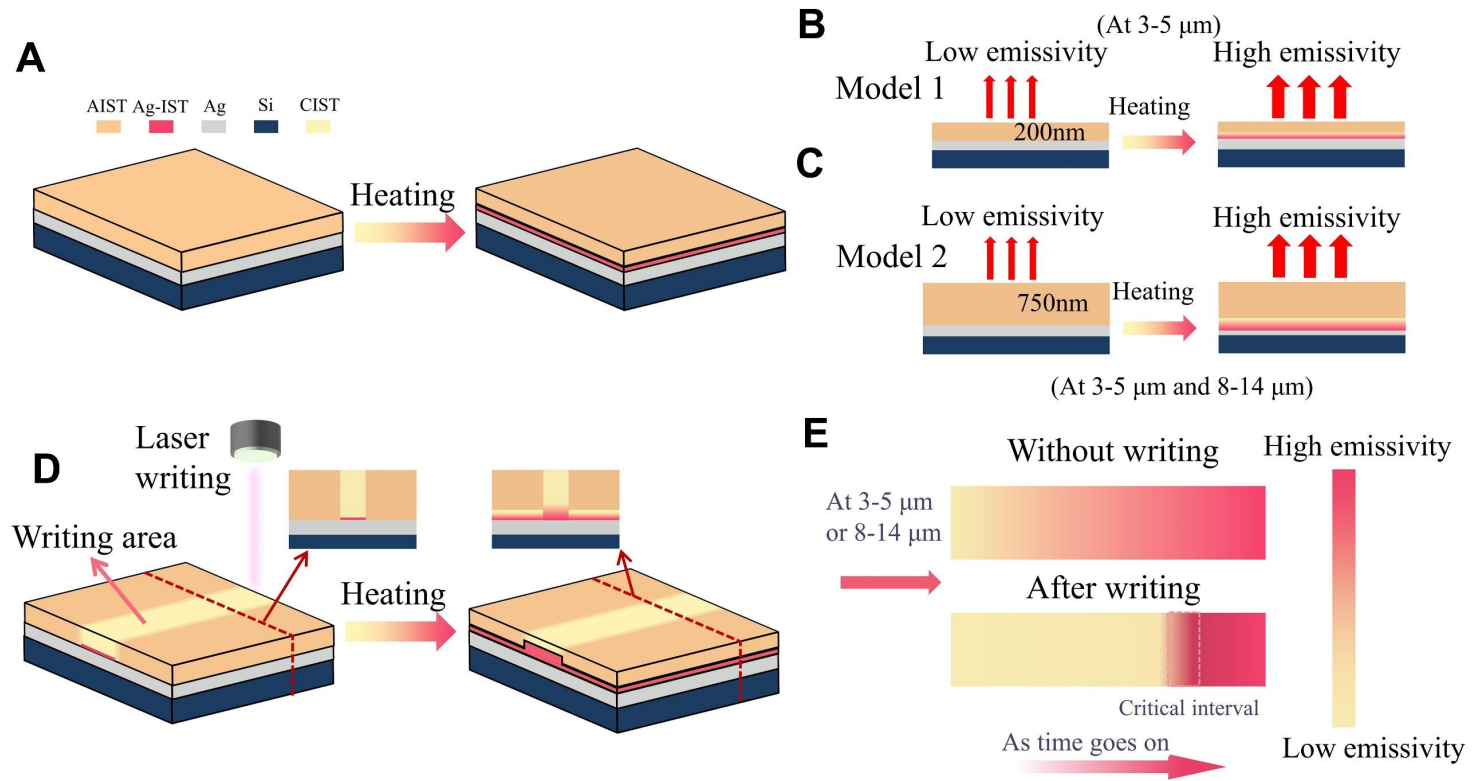

Supplement: Supplementary 1 — Texts S1 to S12 Figs. S1 to S45 Tables S1 and S2 Movies S1 to S3 [file research.1141.f1.zip › S22.pdf]

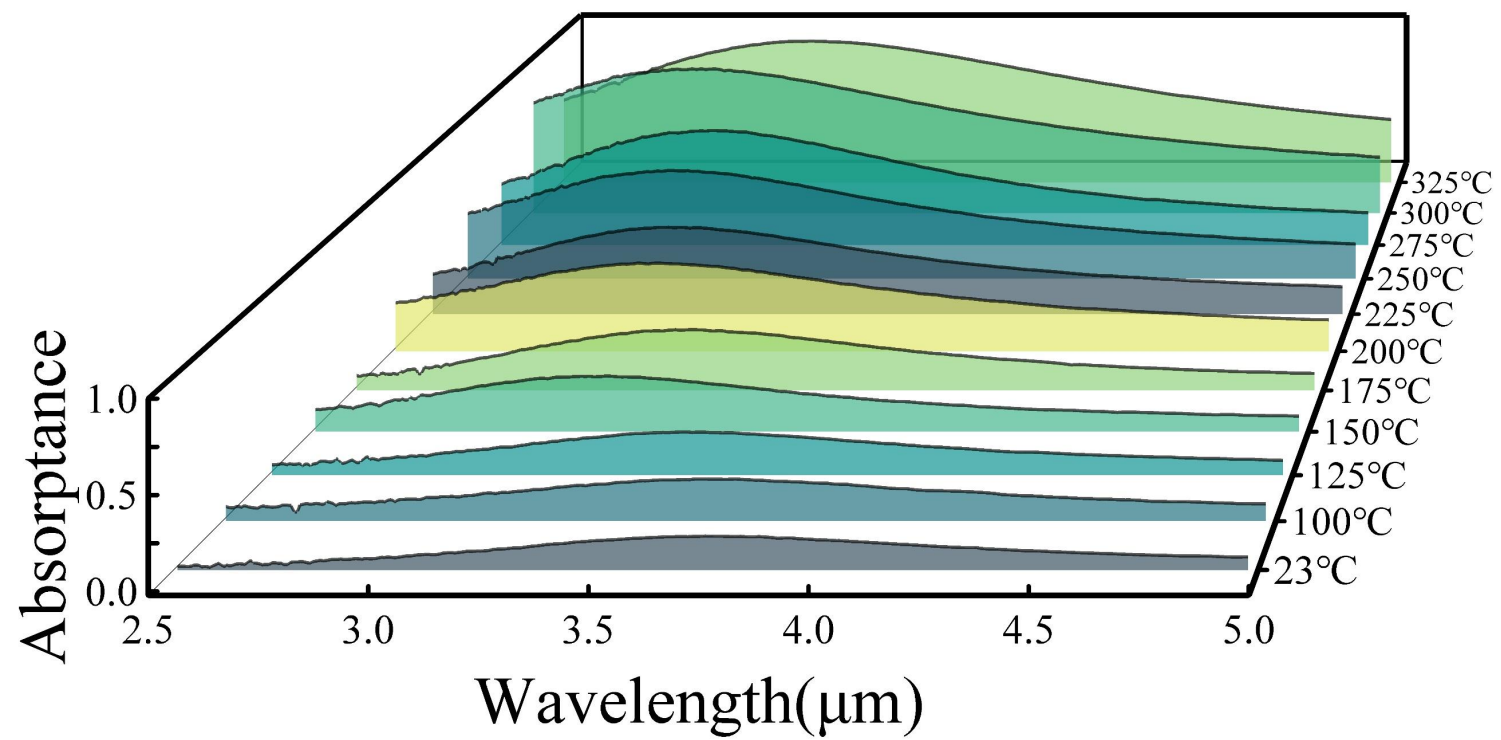

Supplement: Supplementary 1 — Texts S1 to S12 Figs. S1 to S45 Tables S1 and S2 Movies S1 to S3 [file research.1141.f1.zip › S23.pdf]

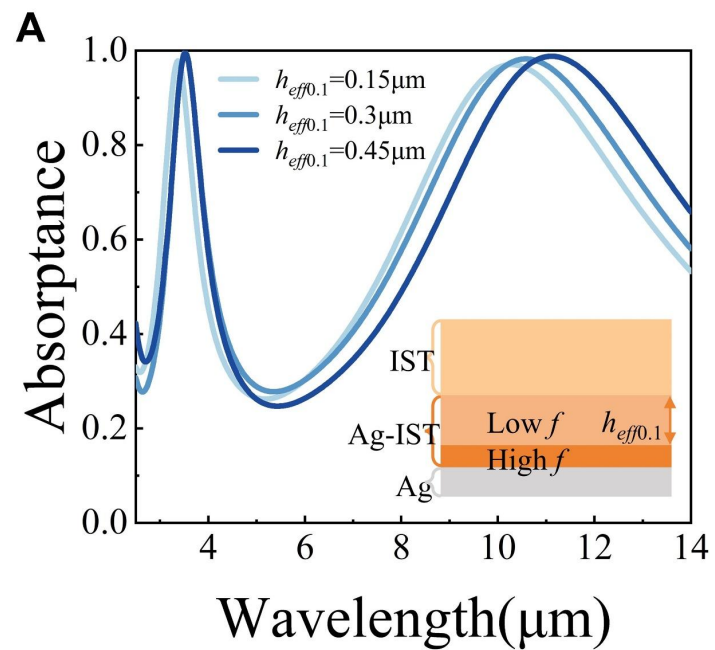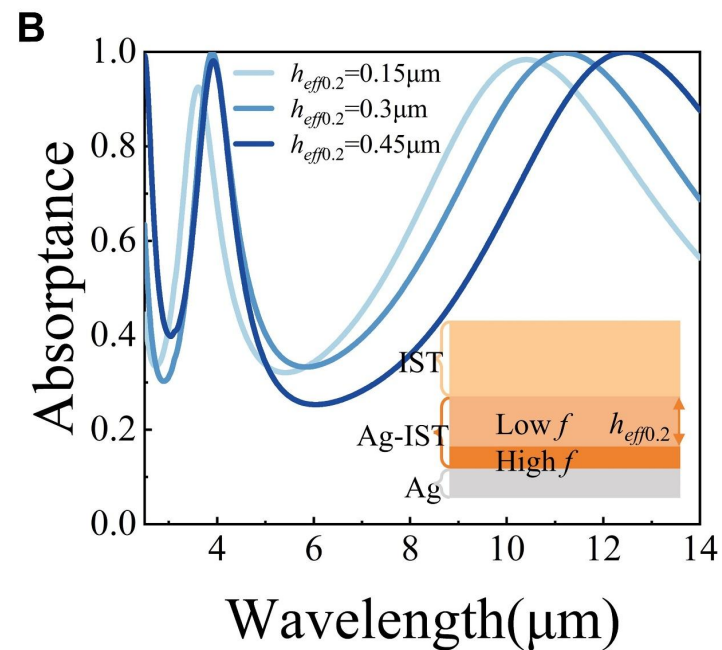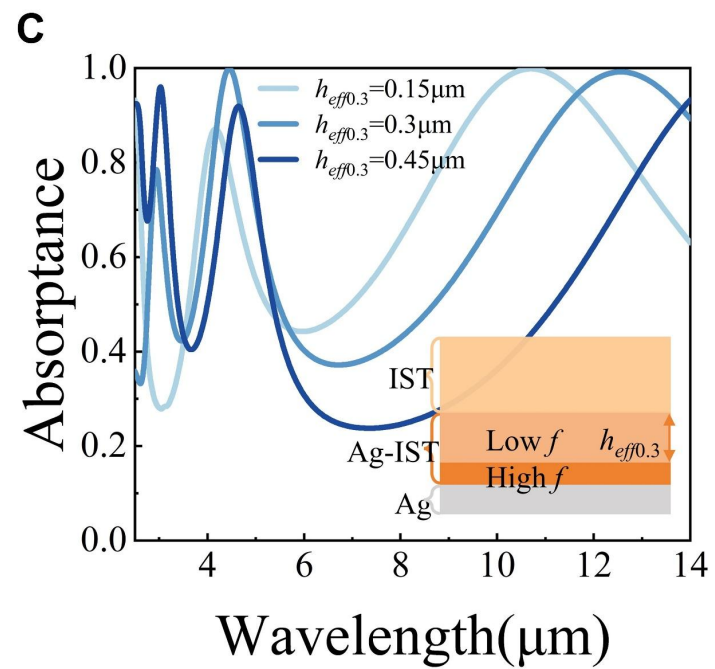

Supplement: Supplementary 1 — Texts S1 to S12 Figs. S1 to S45 Tables S1 and S2 Movies S1 to S3 [file research.1141.f1.zip › S24.pdf]

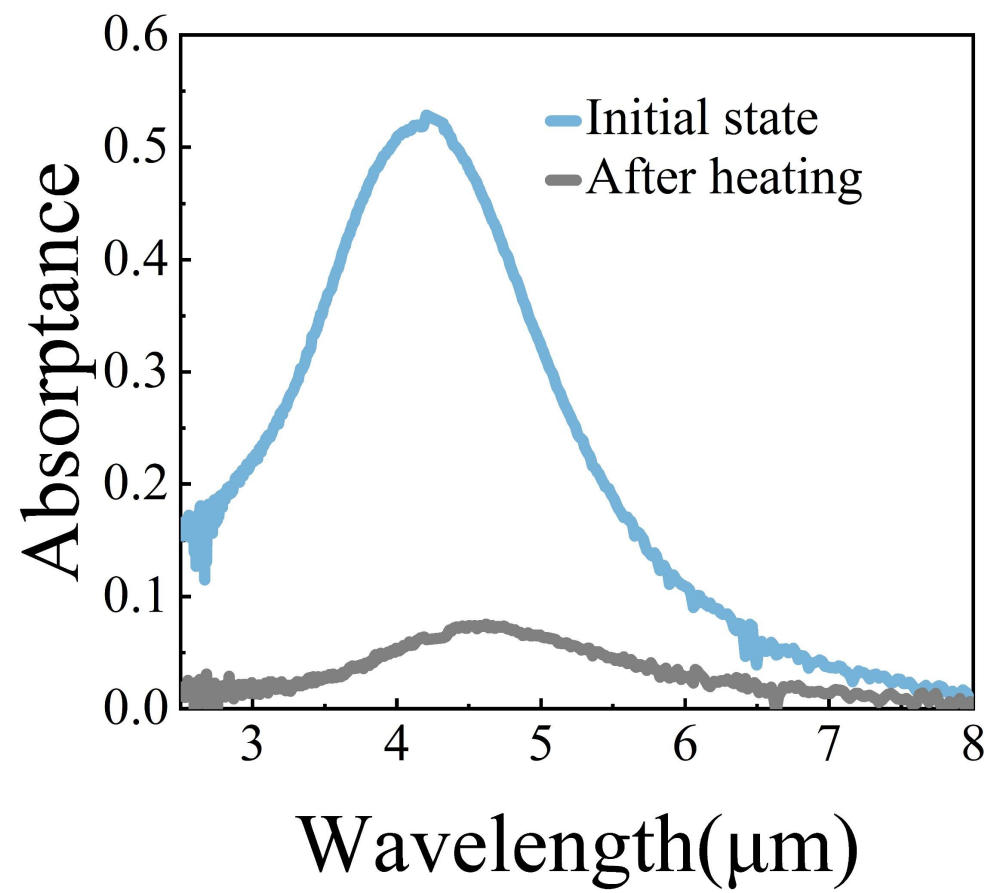

Supplement: Supplementary 1 — Texts S1 to S12 Figs. S1 to S45 Tables S1 and S2 Movies S1 to S3 [file research.1141.f1.zip › S25.pdf]

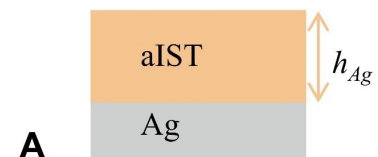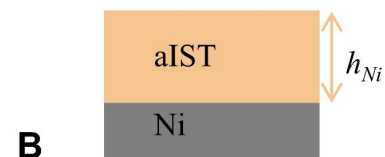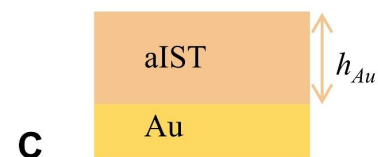

Unheated

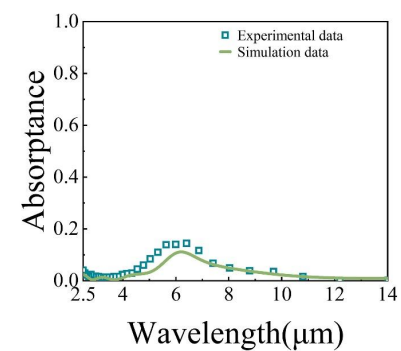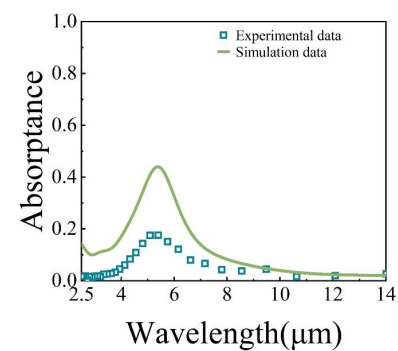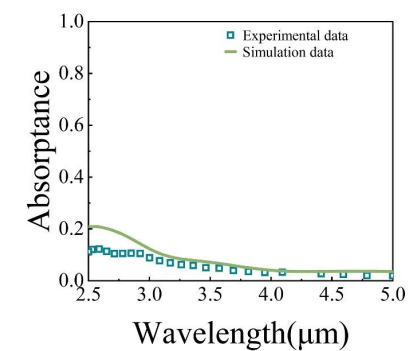

After heating

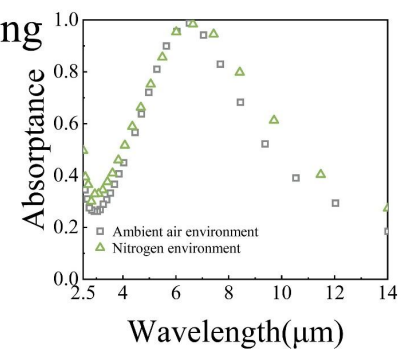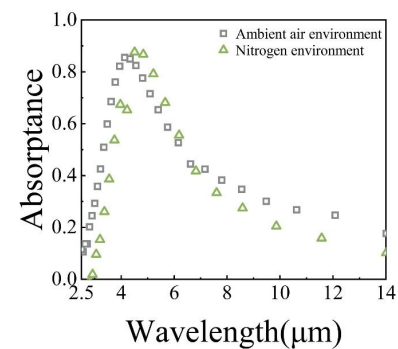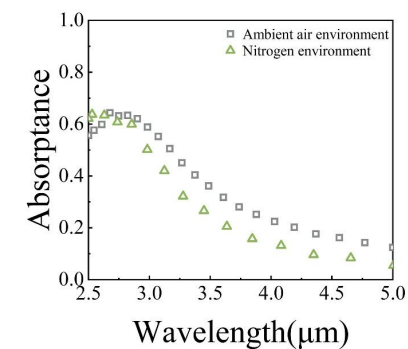

Supplement: Supplementary 1 — Texts S1 to S12 Figs. S1 to S45 Tables S1 and S2 Movies S1 to S3 [file research.1141.f1.zip › S26.pdf]

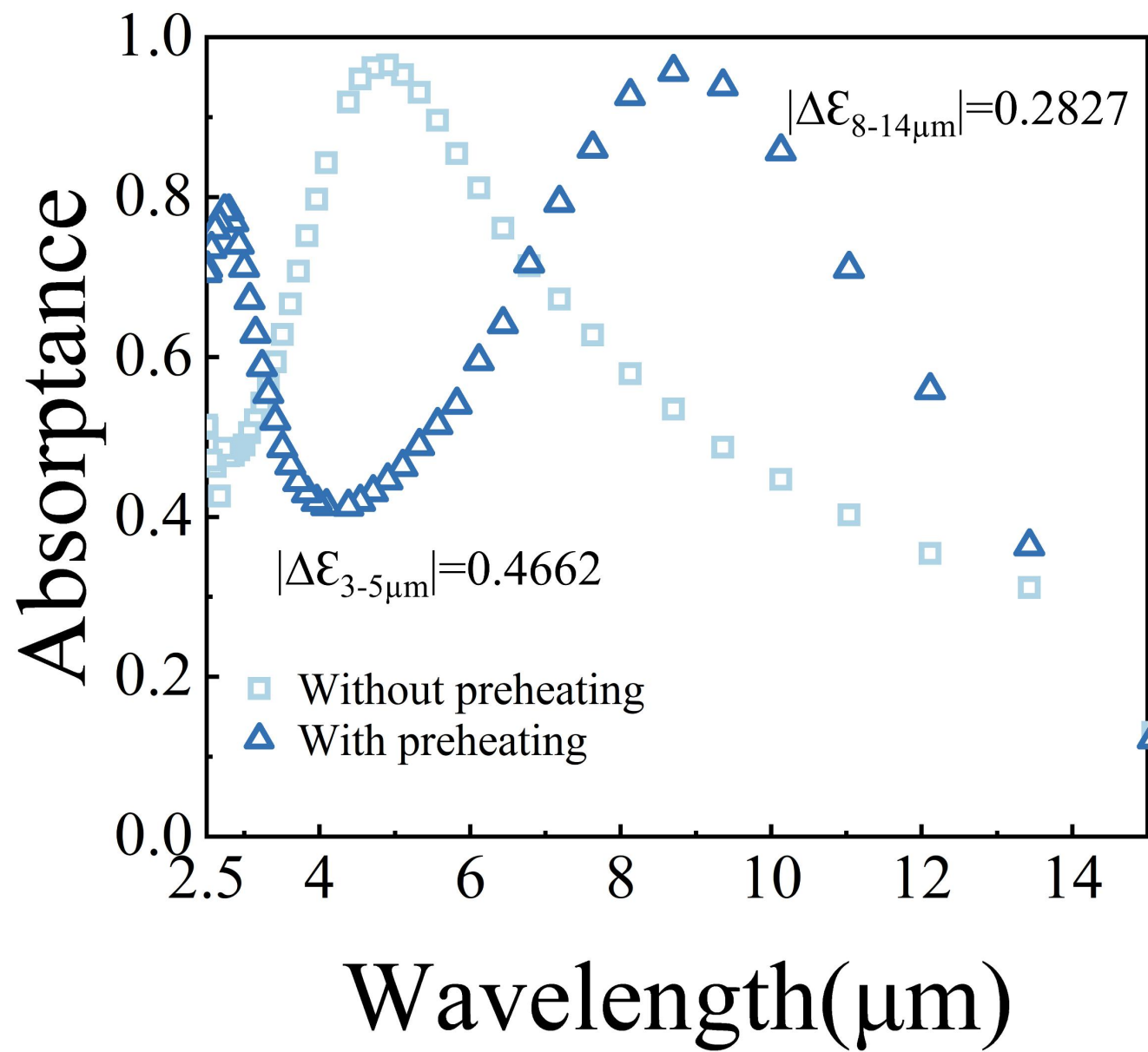

Supplement: Supplementary 1 — Texts S1 to S12 Figs. S1 to S45 Tables S1 and S2 Movies S1 to S3 [file research.1141.f1.zip › S27.pdf]

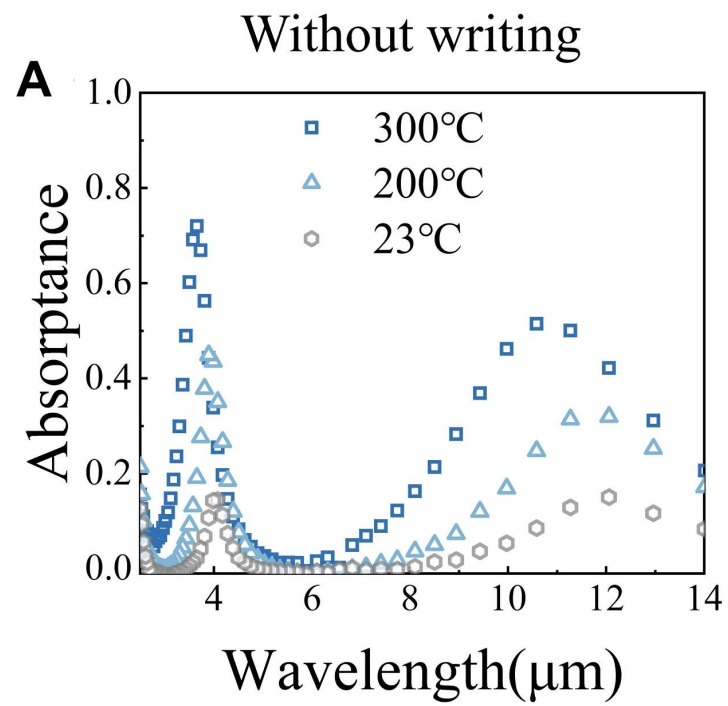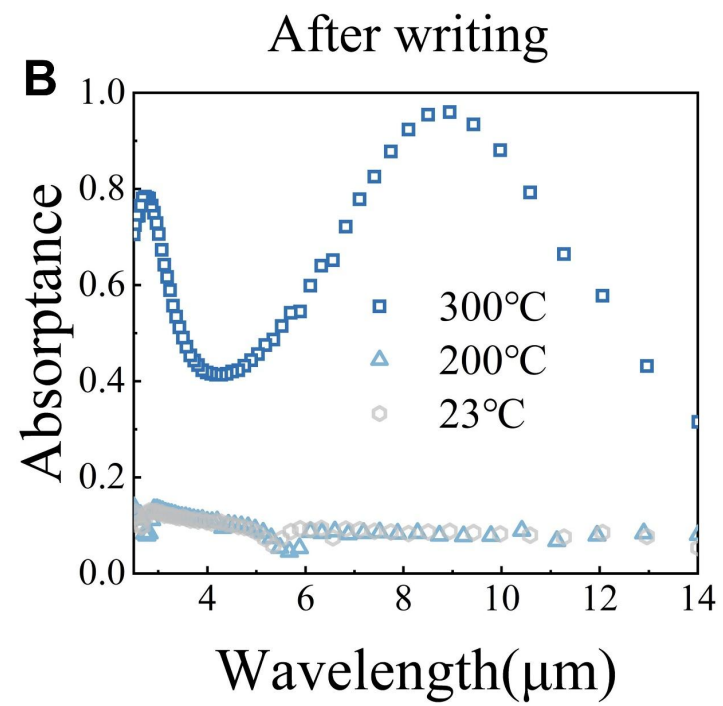

Supplement: Supplementary 1 — Texts S1 to S12 Figs. S1 to S45 Tables S1 and S2 Movies S1 to S3 [file research.1141.f1.zip › S28.pdf]

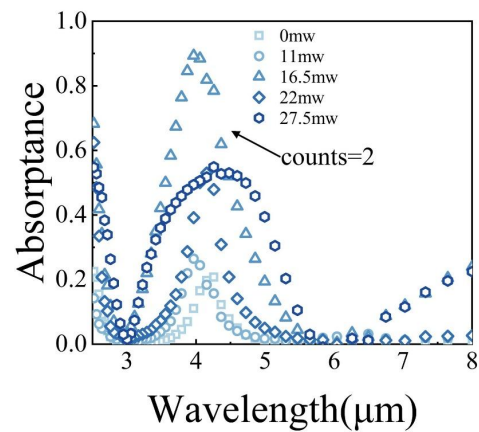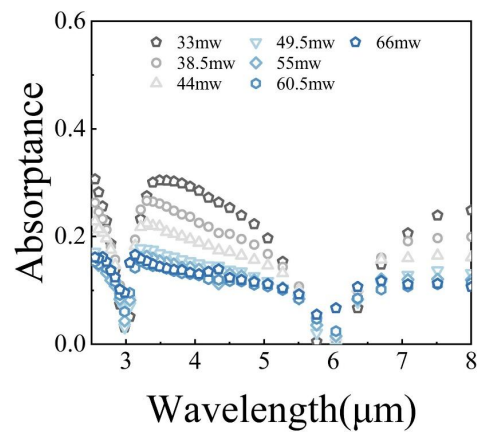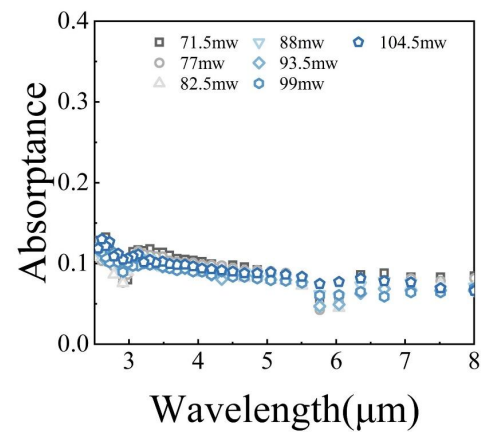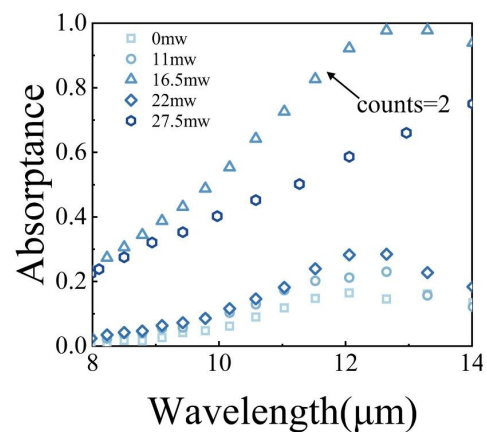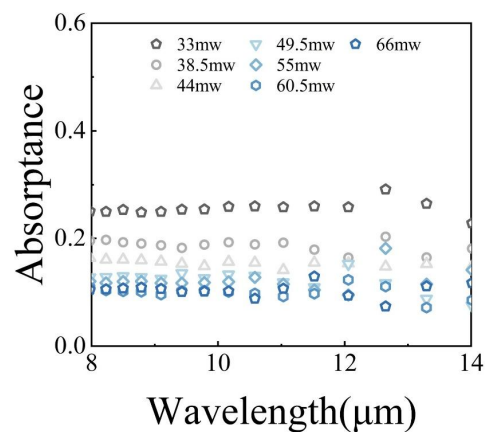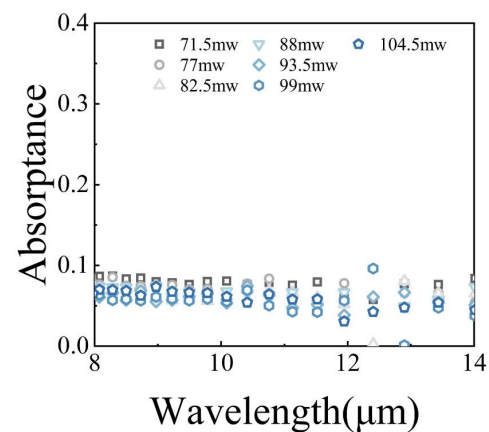

Supplement: Supplementary 1 — Texts S1 to S12 Figs. S1 to S45 Tables S1 and S2 Movies S1 to S3 [file research.1141.f1.zip › S29.pdf]

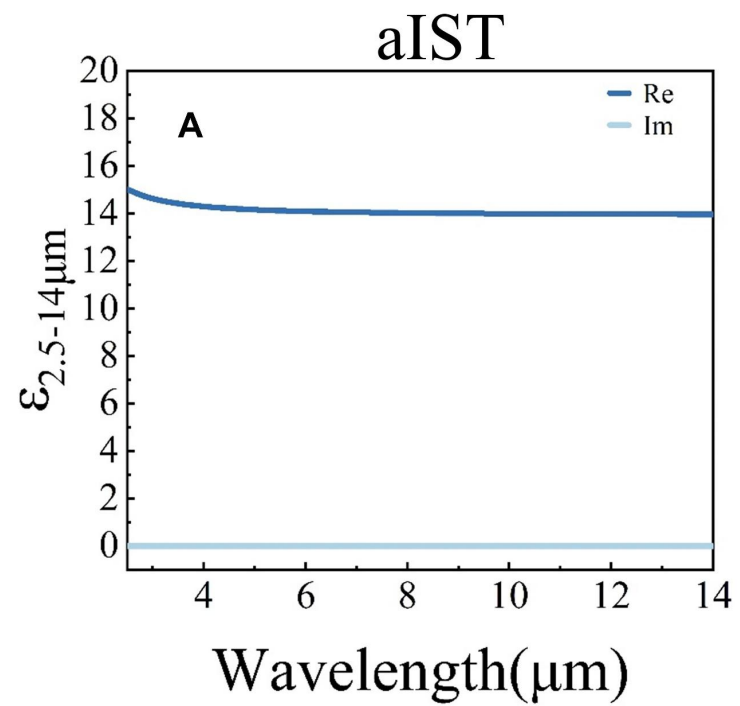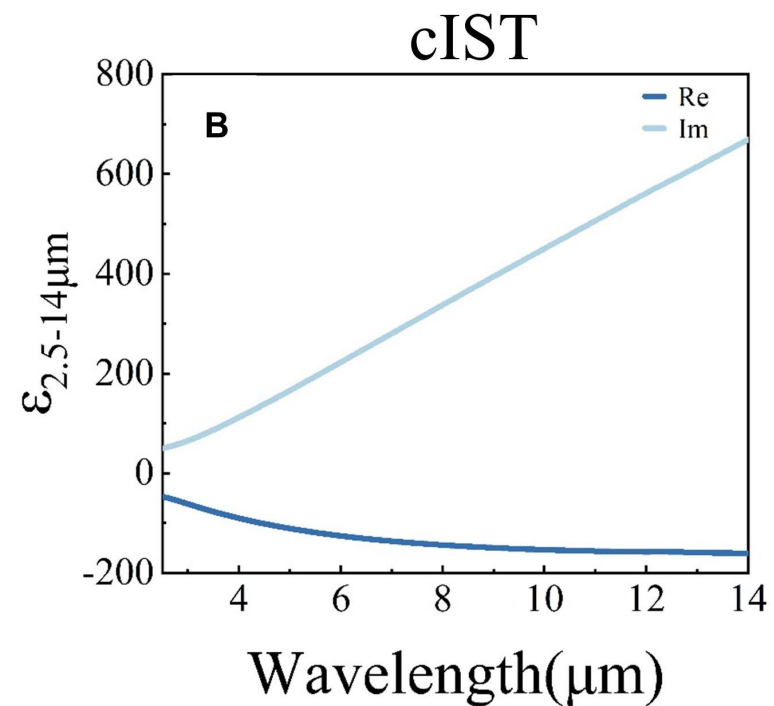

Supplement: Supplementary 1 — Texts S1 to S12 Figs. S1 to S45 Tables S1 and S2 Movies S1 to S3 [file research.1141.f1.zip › S3.pdf]

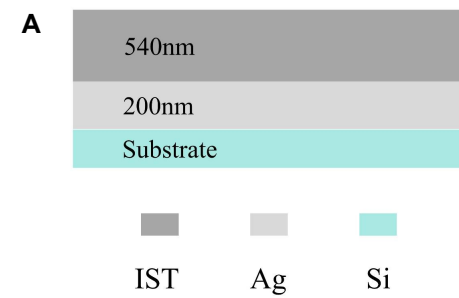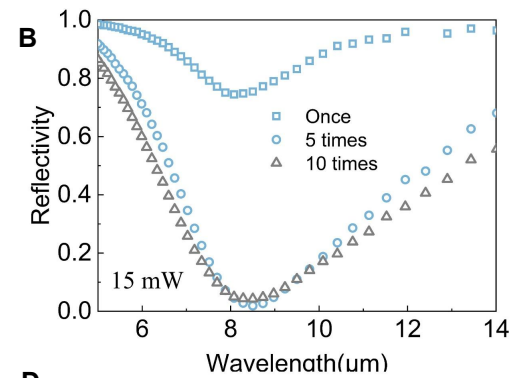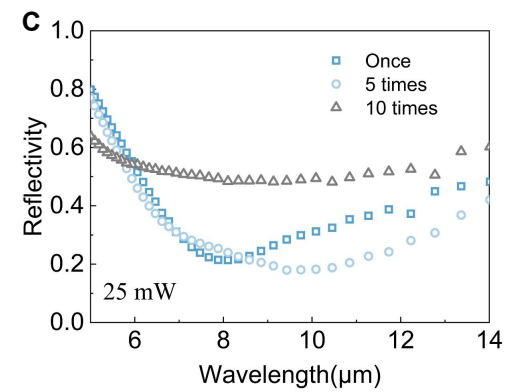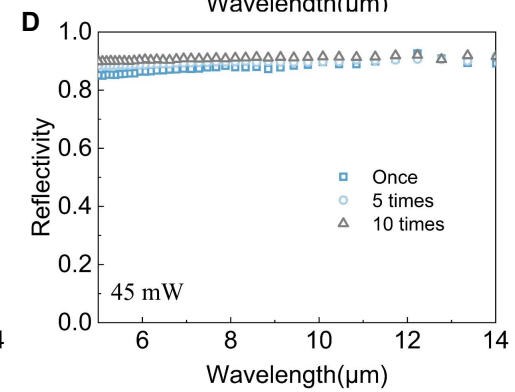

Supplement: Supplementary 1 — Texts S1 to S12 Figs. S1 to S45 Tables S1 and S2 Movies S1 to S3 [file research.1141.f1.zip › S30.pdf]

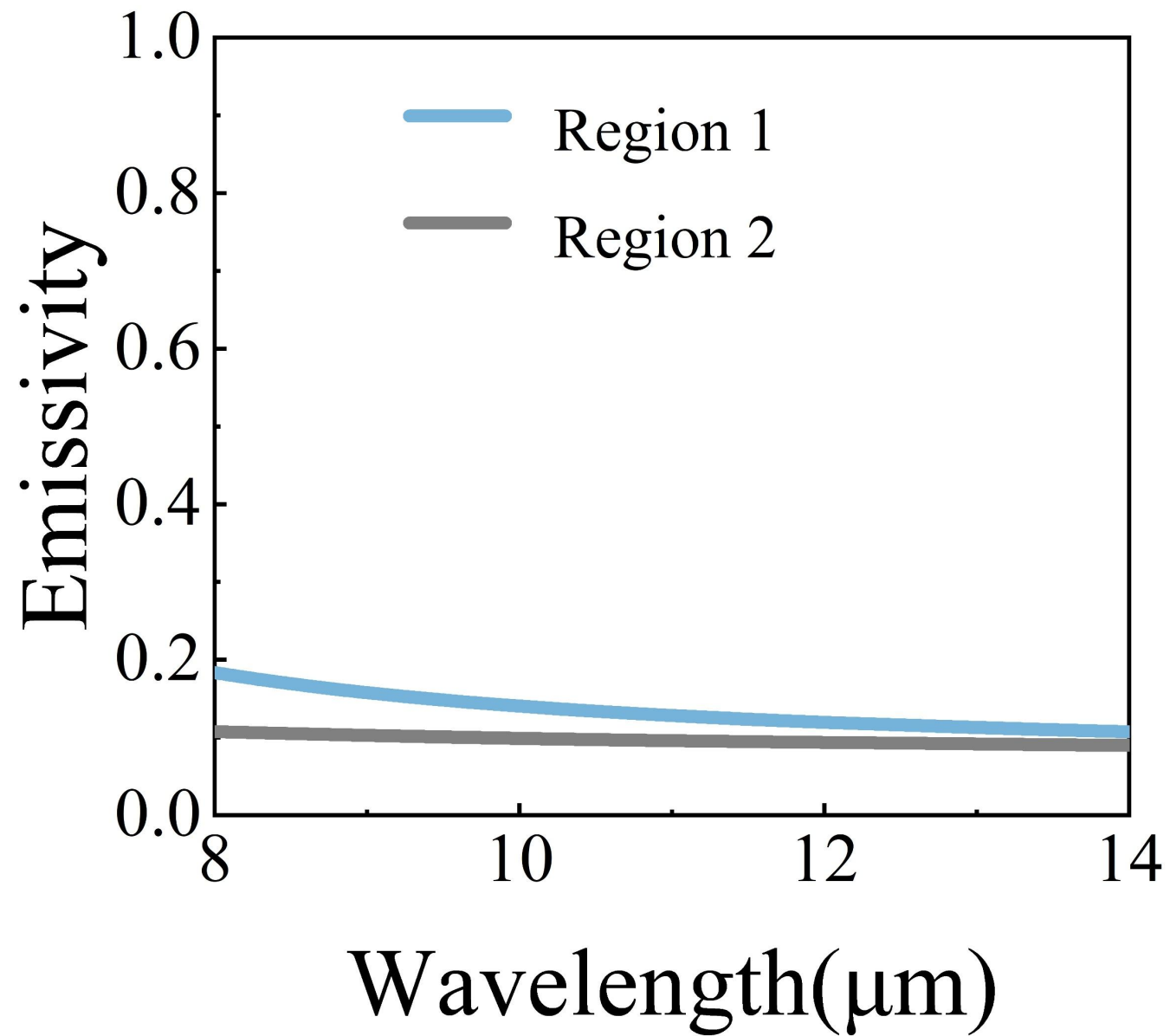

Supplement: Supplementary 1 — Texts S1 to S12 Figs. S1 to S45 Tables S1 and S2 Movies S1 to S3 [file research.1141.f1.zip › S31.pdf]

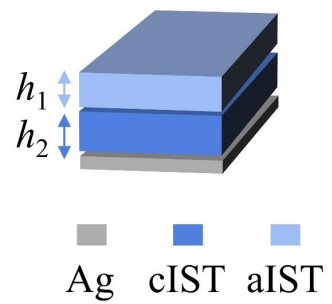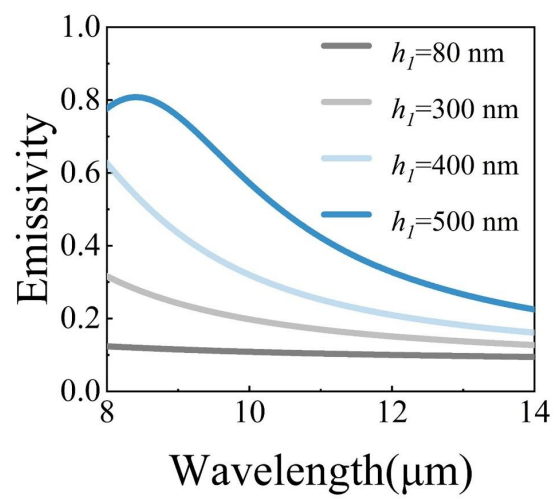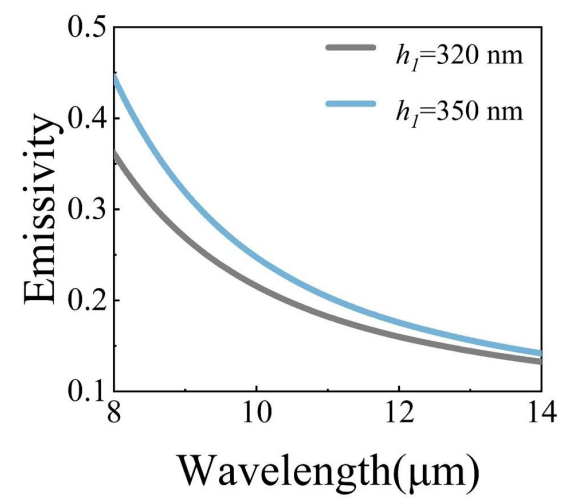

Supplement: Supplementary 1 — Texts S1 to S12 Figs. S1 to S45 Tables S1 and S2 Movies S1 to S3 [file research.1141.f1.zip › S32.pdf]

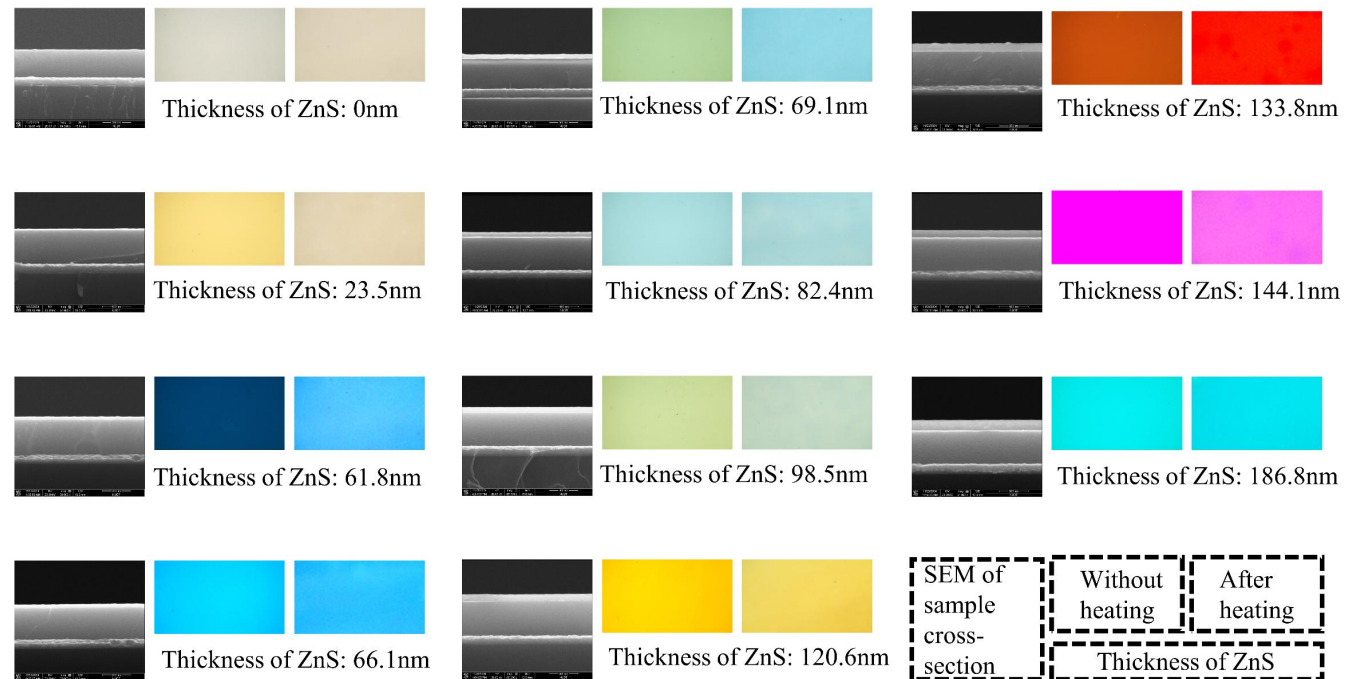

Supplement: Supplementary 1 — Texts S1 to S12 Figs. S1 to S45 Tables S1 and S2 Movies S1 to S3 [file research.1141.f1.zip › S33.pdf]

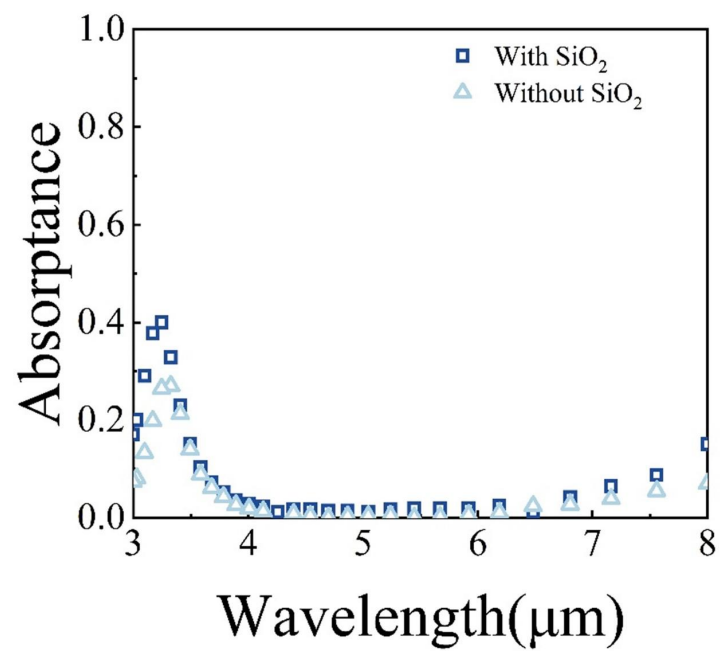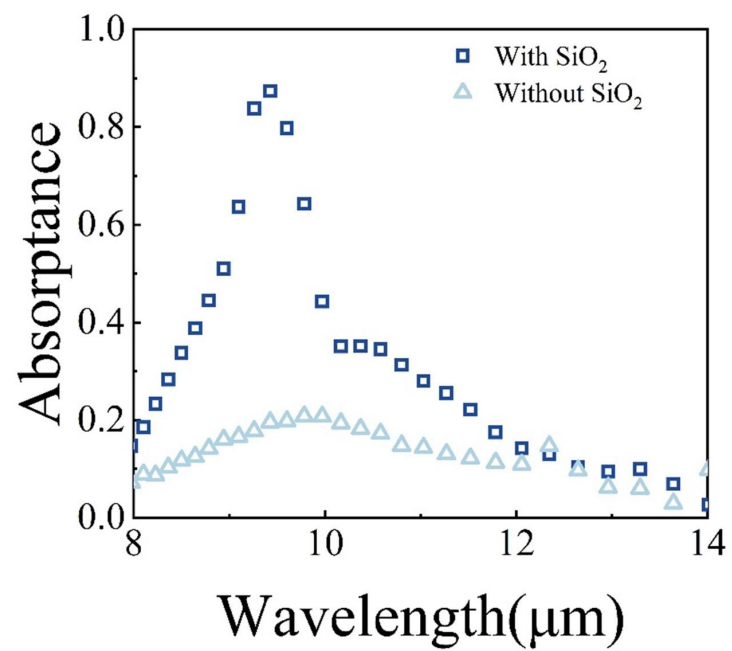

Supplement: Supplementary 1 — Texts S1 to S12 Figs. S1 to S45 Tables S1 and S2 Movies S1 to S3 [file research.1141.f1.zip › S34.pdf]

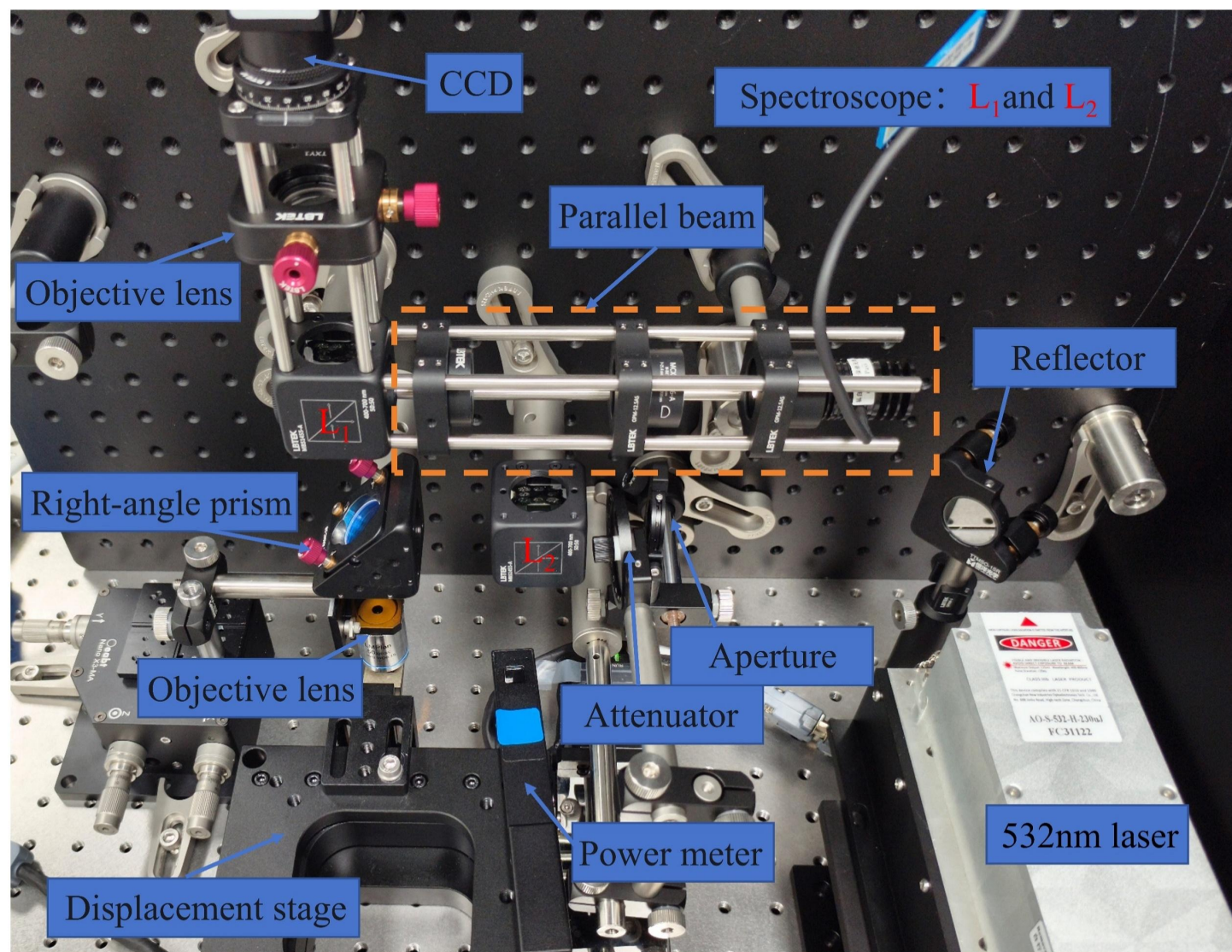

Supplement: Supplementary 1 — Texts S1 to S12 Figs. S1 to S45 Tables S1 and S2 Movies S1 to S3 [file research.1141.f1.zip › S35.pdf]

**A**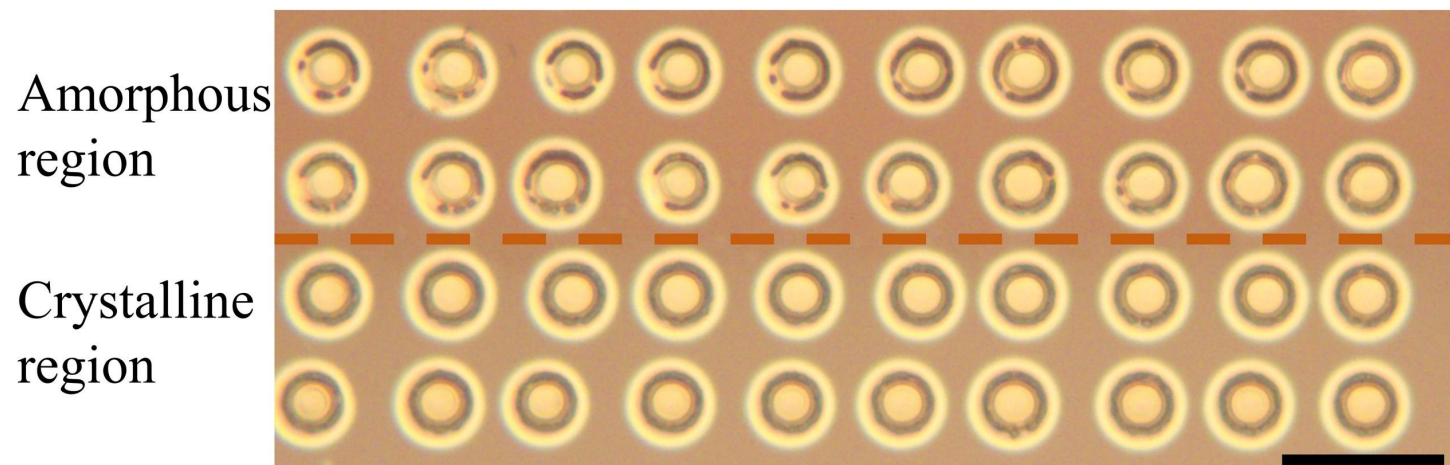**B**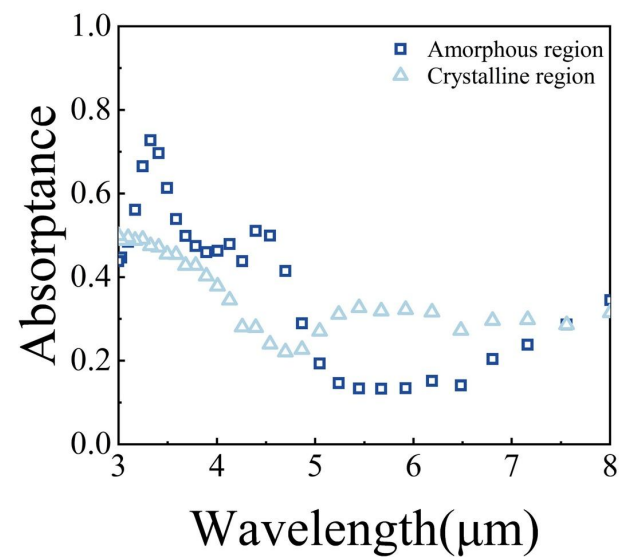**C**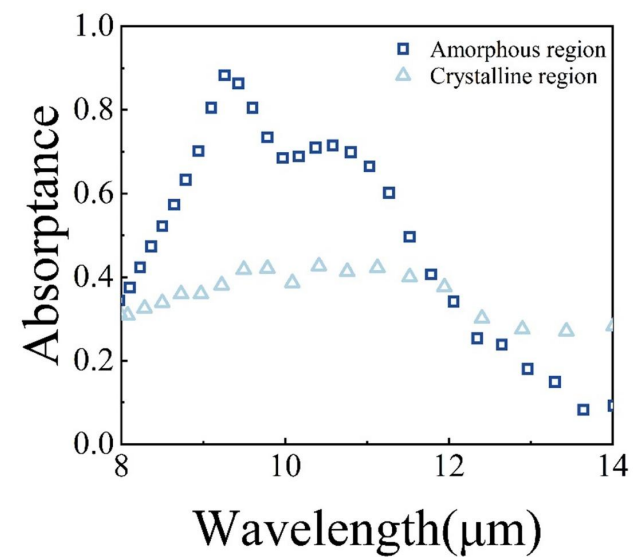

Supplement: Supplementary 1 — Texts S1 to S12 Figs. S1 to S45 Tables S1 and S2 Movies S1 to S3 [file research.1141.f1.zip › S36.pdf]

**A** After erasing

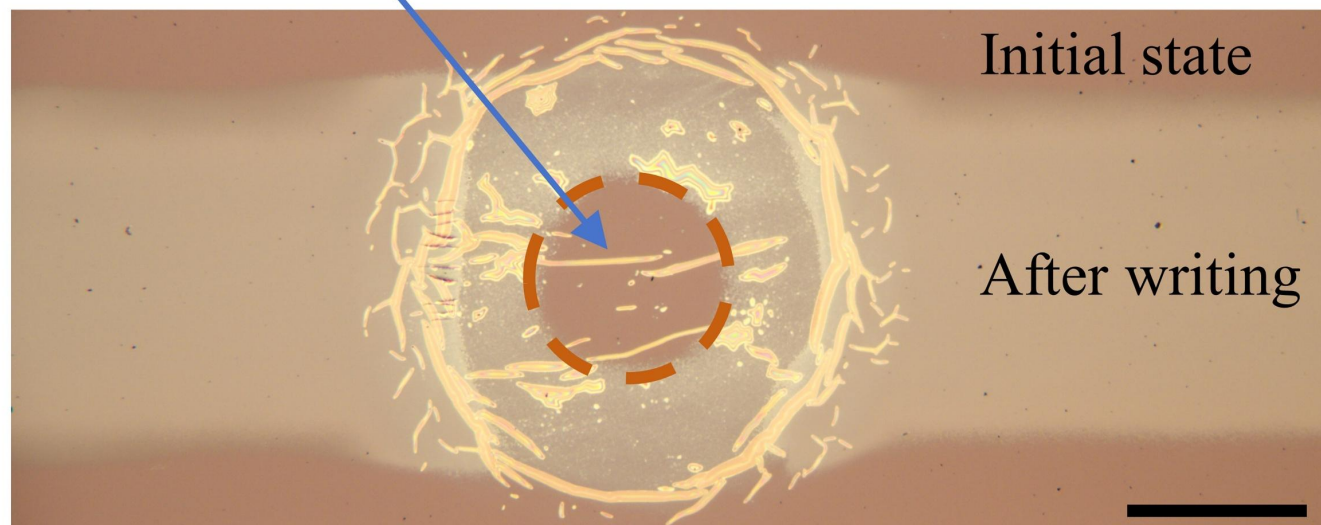

**B**

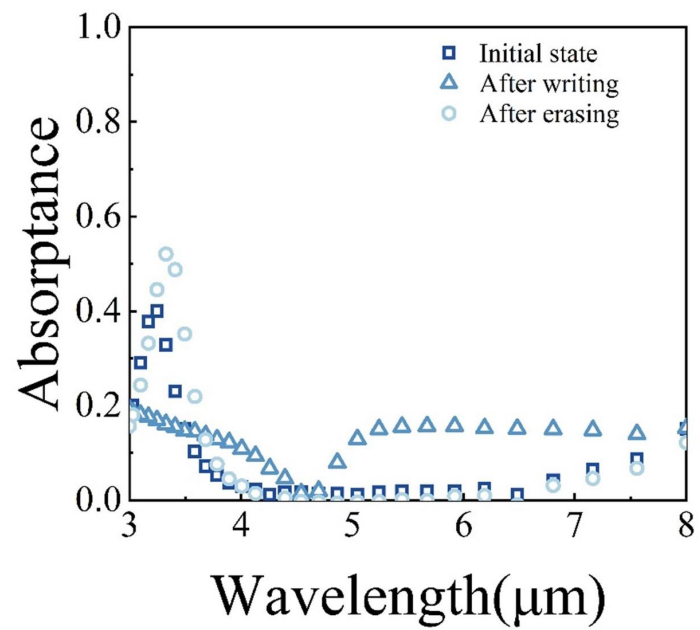

**C**

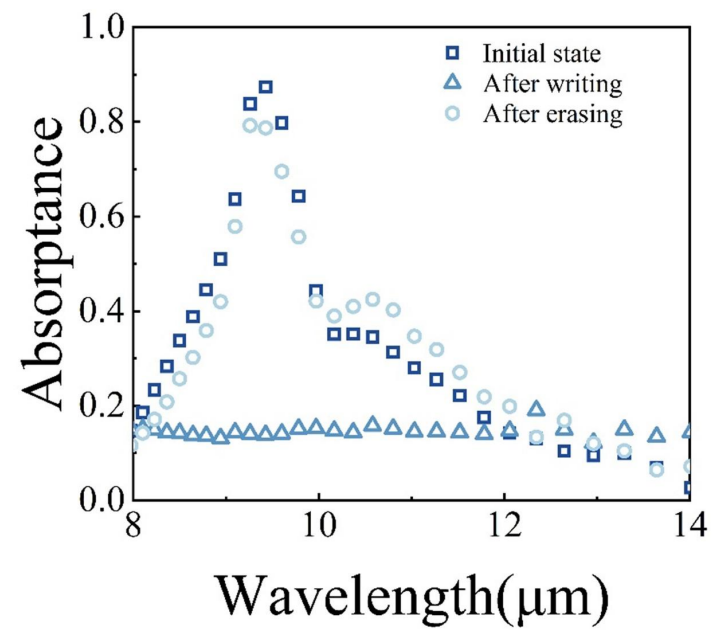

Supplement: Supplementary 1 — Texts S1 to S12 Figs. S1 to S45 Tables S1 and S2 Movies S1 to S3 [file research.1141.f1.zip › S37.pdf]

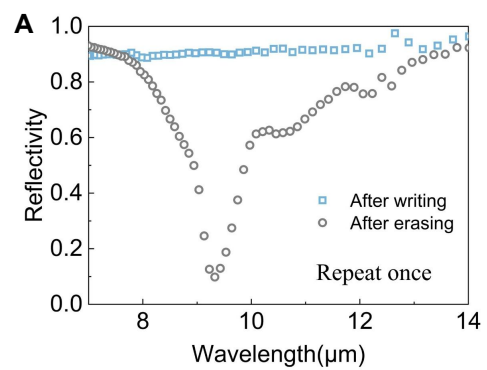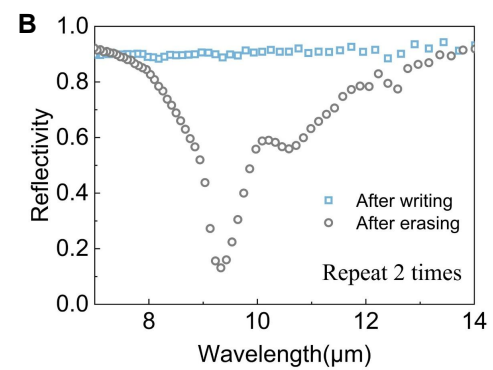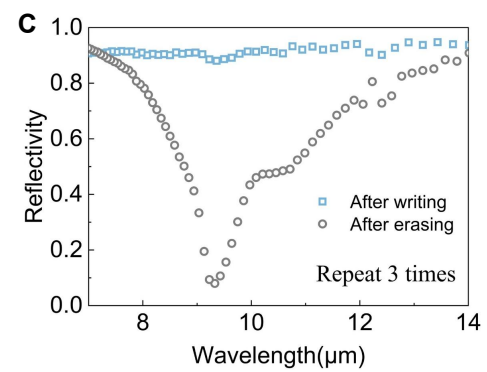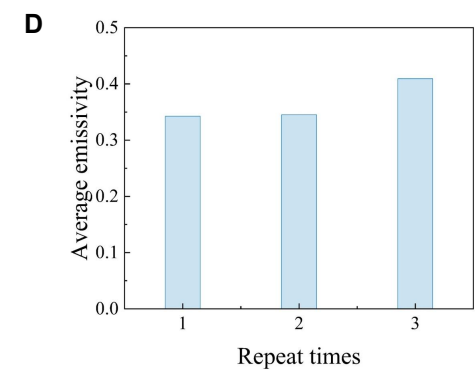

Supplement: Supplementary 1 — Texts S1 to S12 Figs. S1 to S45 Tables S1 and S2 Movies S1 to S3 [file research.1141.f1.zip › S38.pdf]

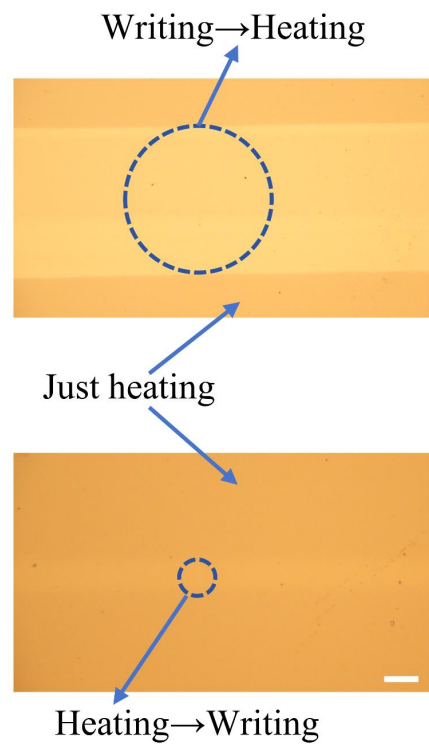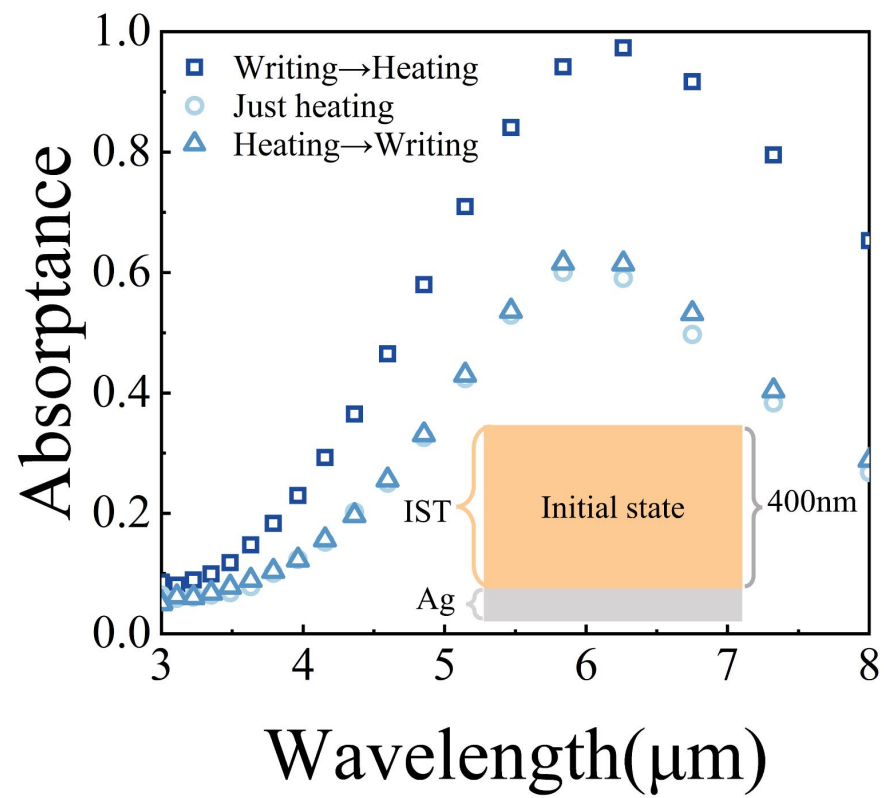

Supplement: Supplementary 1 — Texts S1 to S12 Figs. S1 to S45 Tables S1 and S2 Movies S1 to S3 [file research.1141.f1.zip › S39.pdf]

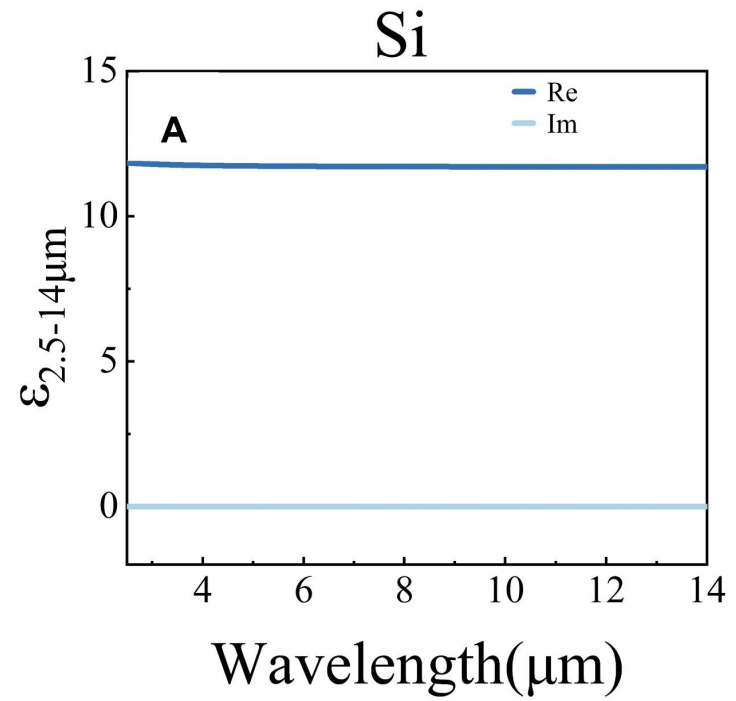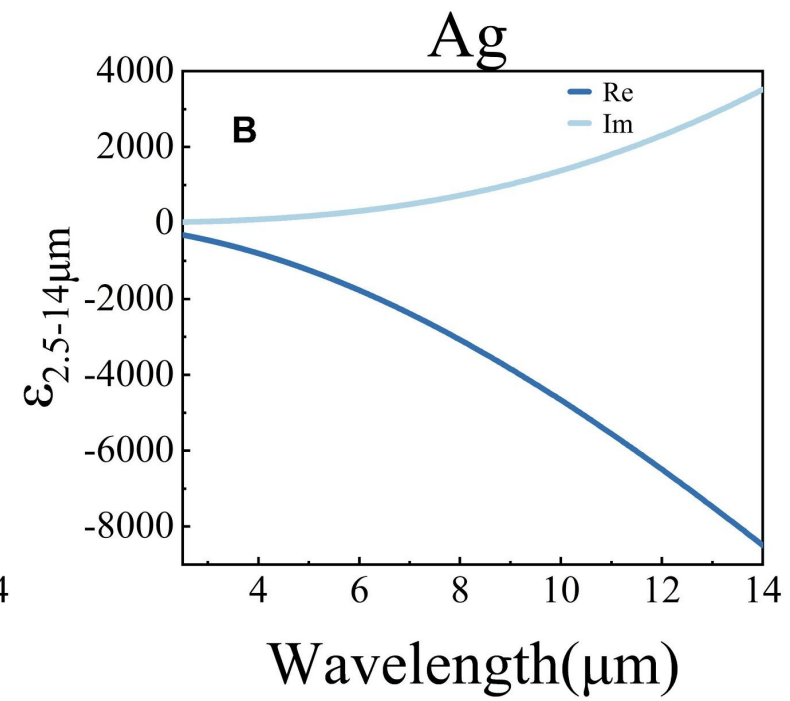

Supplement: Supplementary 1 — Texts S1 to S12 Figs. S1 to S45 Tables S1 and S2 Movies S1 to S3 [file research.1141.f1.zip › S4.pdf]

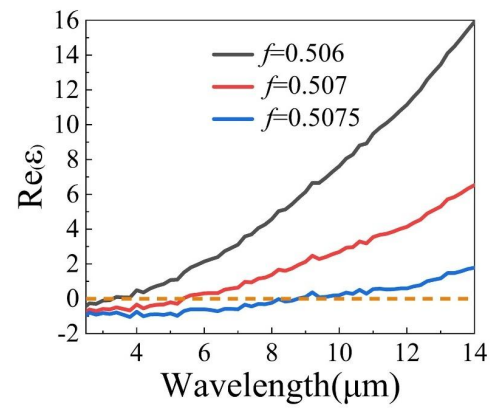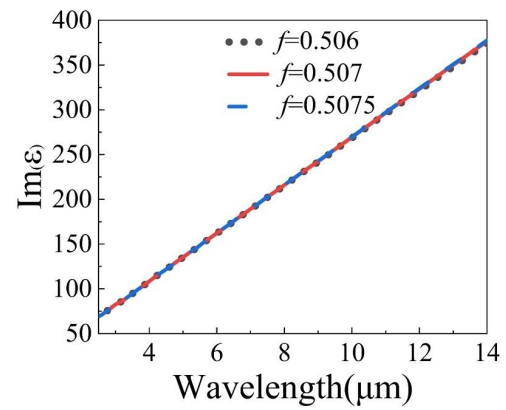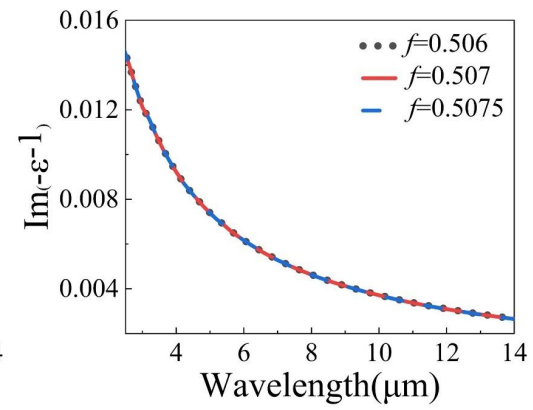

Supplement: Supplementary 1 — Texts S1 to S12 Figs. S1 to S45 Tables S1 and S2 Movies S1 to S3 [file research.1141.f1.zip › S40.pdf]

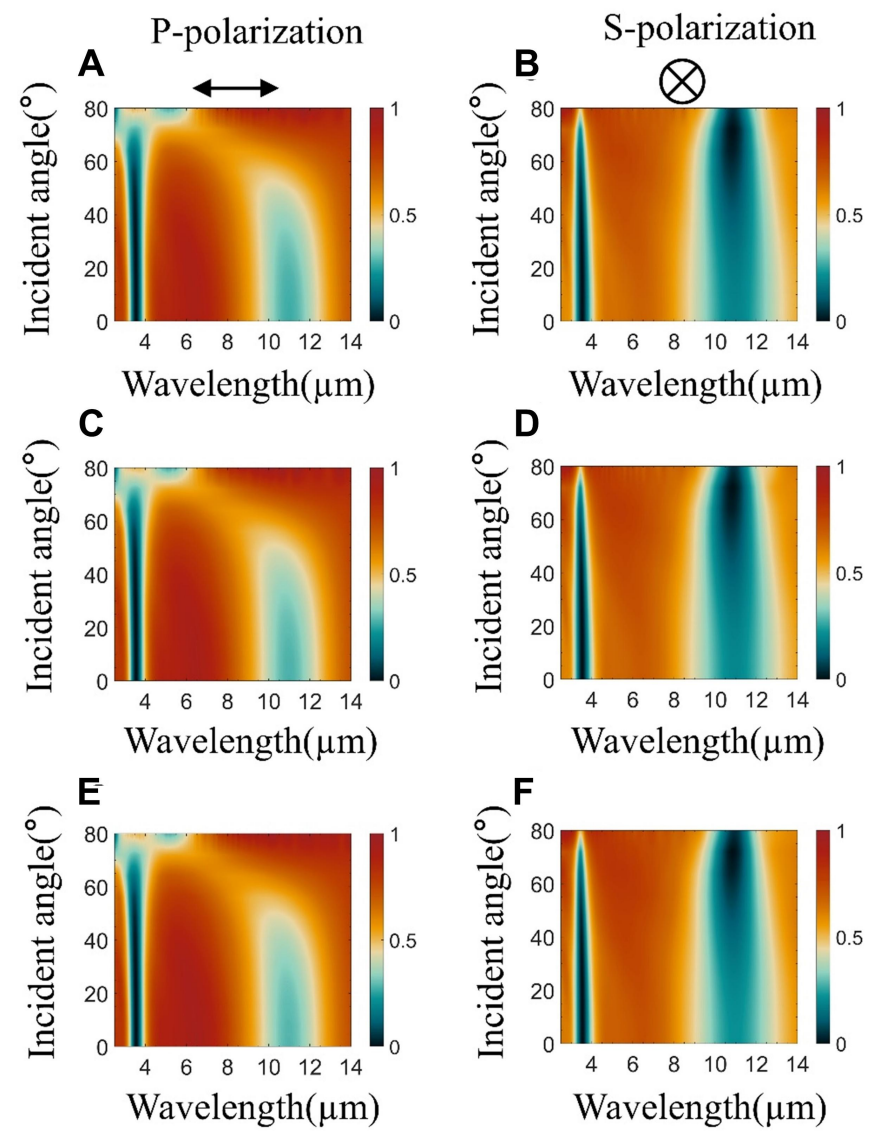

Supplement: Supplementary 1 — Texts S1 to S12 Figs. S1 to S45 Tables S1 and S2 Movies S1 to S3 [file research.1141.f1.zip › S41.pdf]

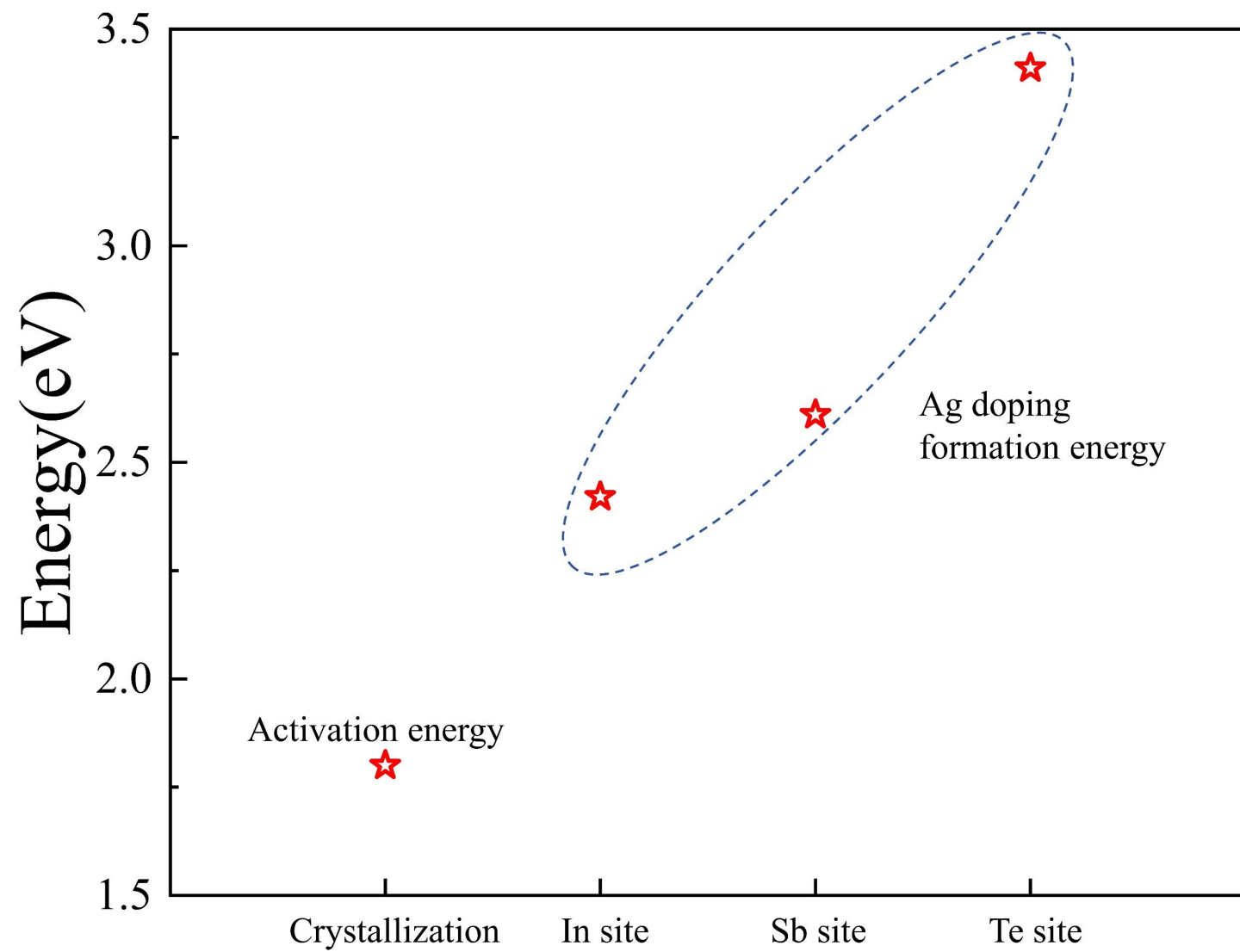

Supplement: Supplementary 1 — Texts S1 to S12 Figs. S1 to S45 Tables S1 and S2 Movies S1 to S3 [file research.1141.f1.zip › S42.pdf]

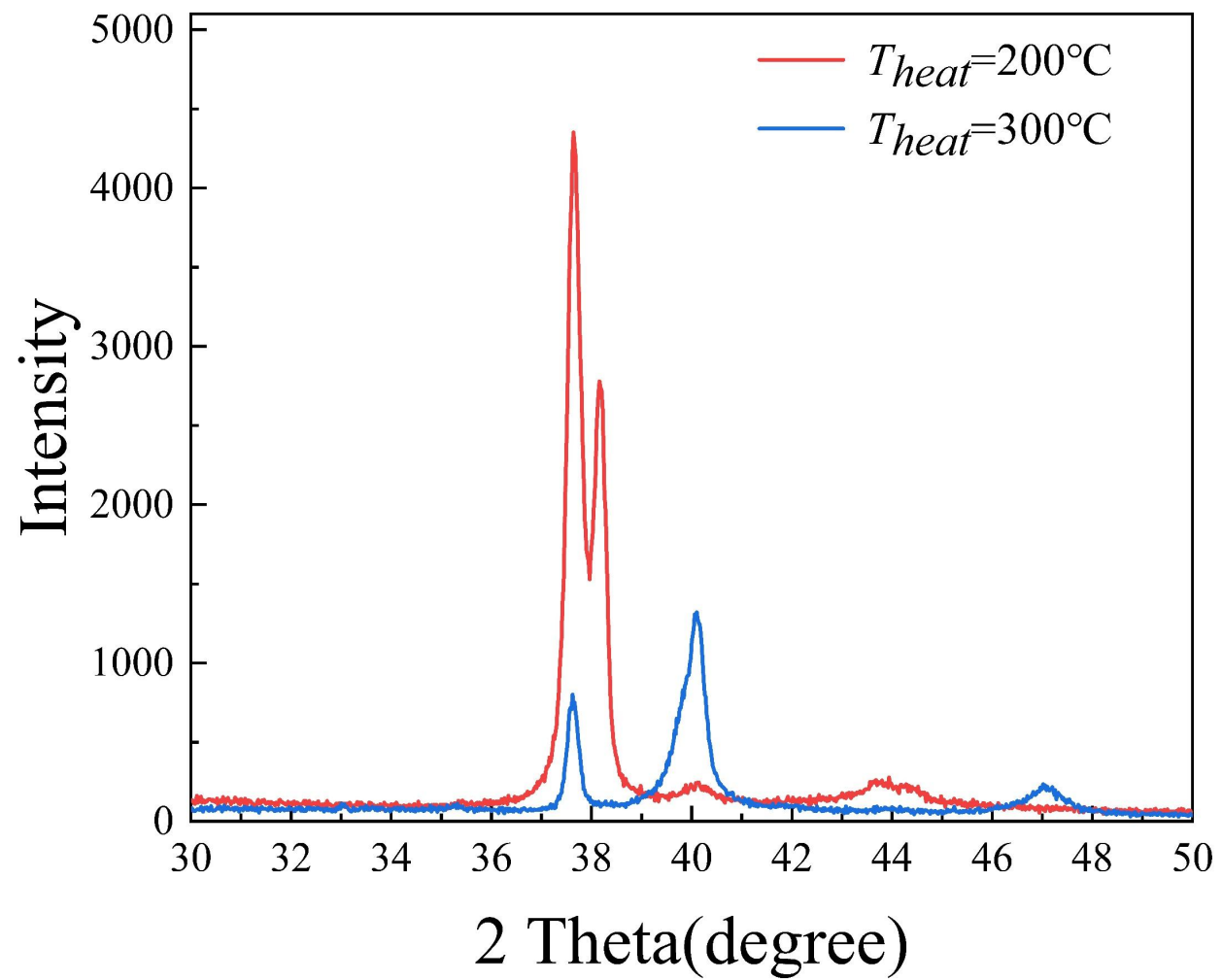

Supplement: Supplementary 1 — Texts S1 to S12 Figs. S1 to S45 Tables S1 and S2 Movies S1 to S3 [file research.1141.f1.zip › S43.pdf]

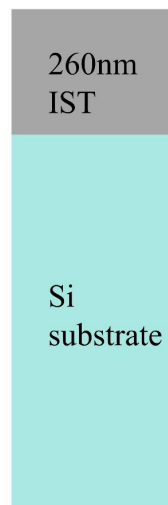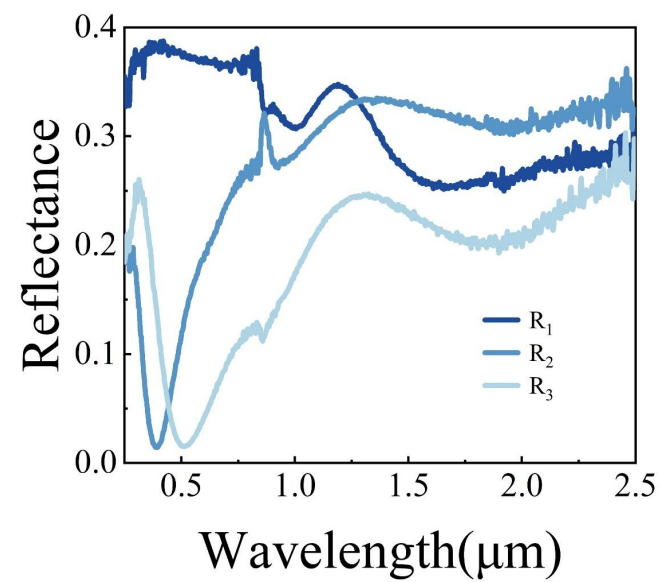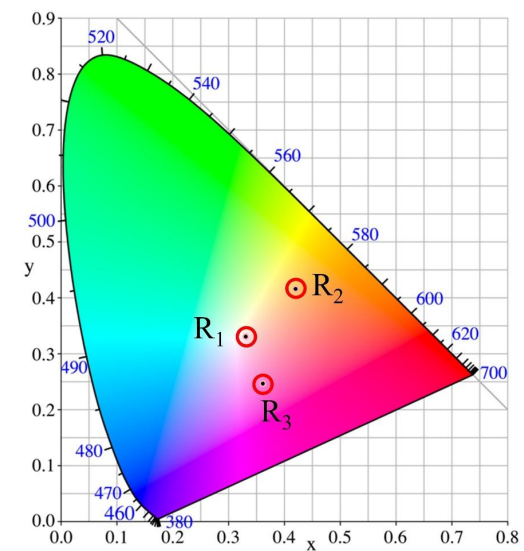

Supplement: Supplementary 1 — Texts S1 to S12 Figs. S1 to S45 Tables S1 and S2 Movies S1 to S3 [file research.1141.f1.zip › S44.pdf]

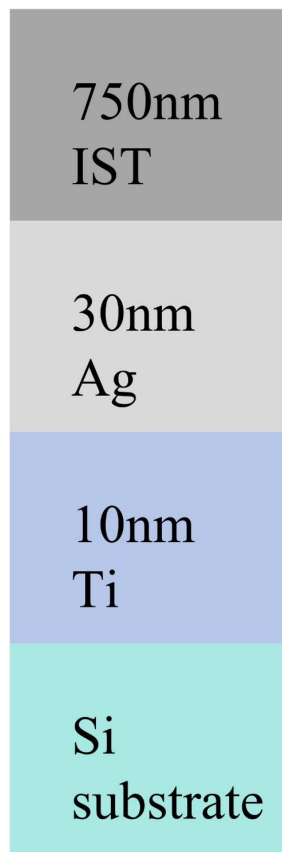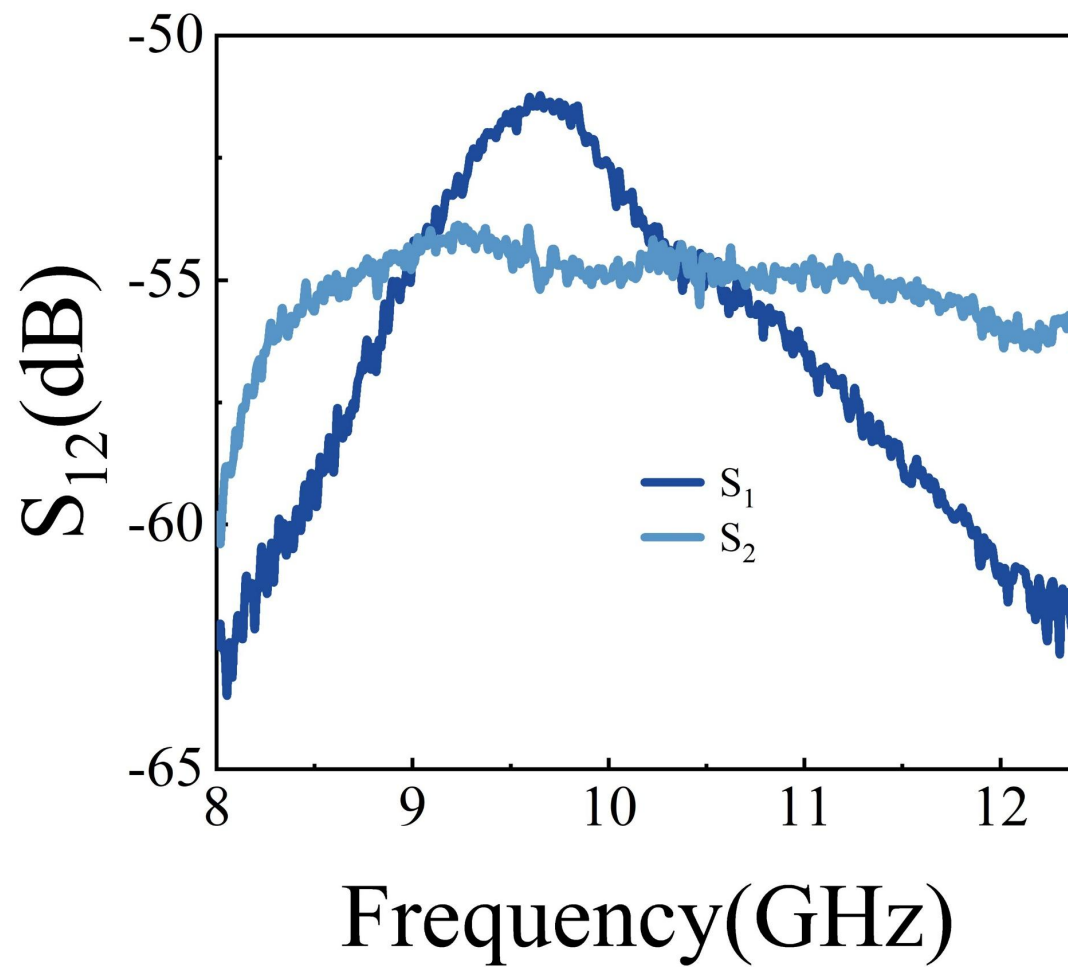

Supplement: Supplementary 1 — Texts S1 to S12 Figs. S1 to S45 Tables S1 and S2 Movies S1 to S3 [file research.1141.f1.zip › S45.pdf]

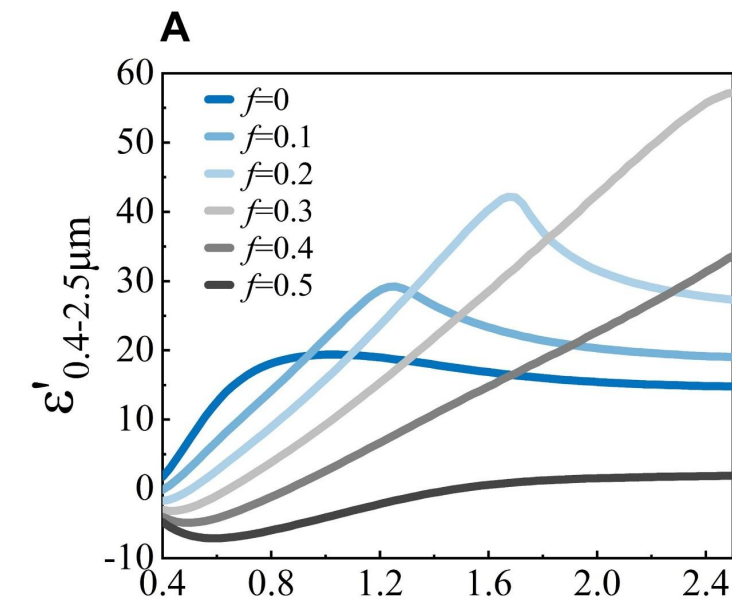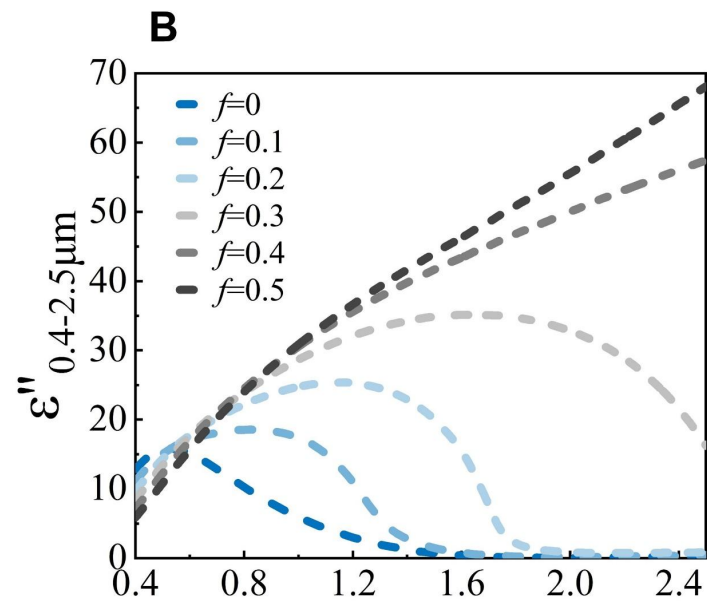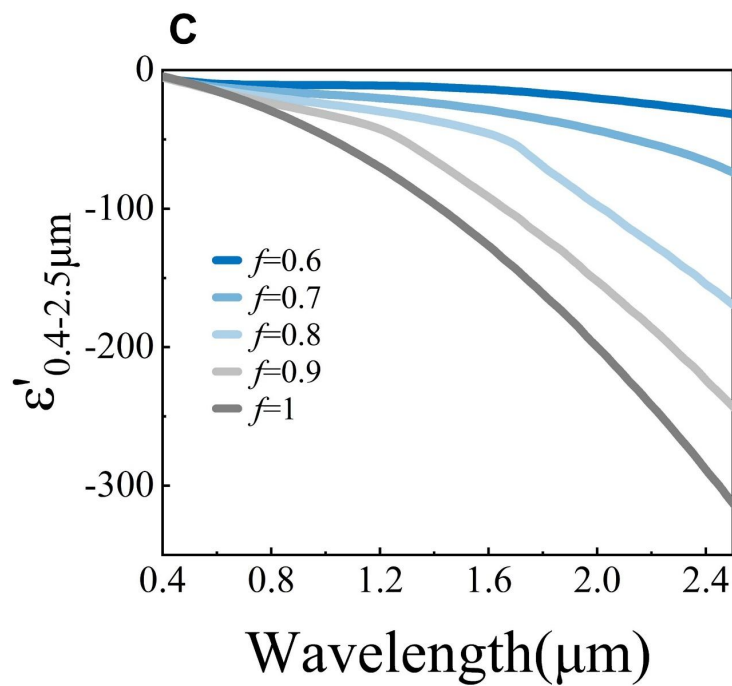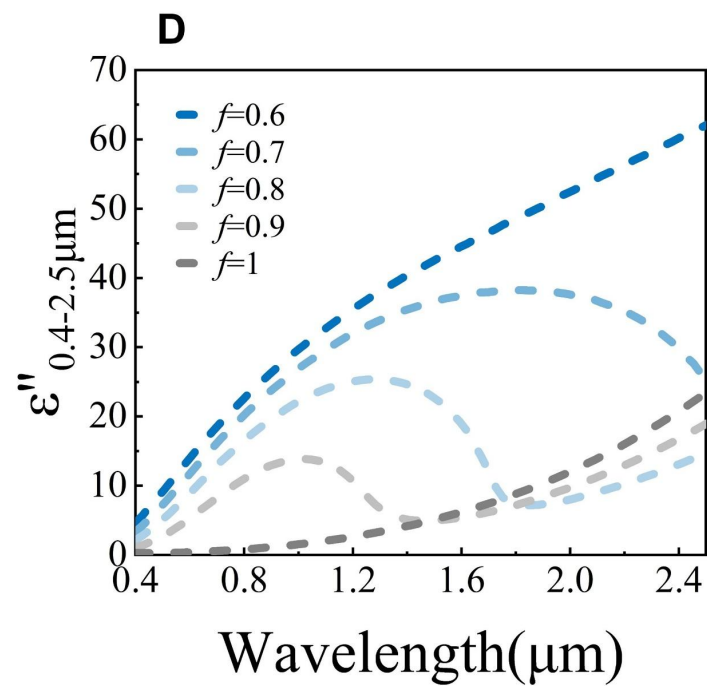

Supplement: Supplementary 1 — Texts S1 to S12 Figs. S1 to S45 Tables S1 and S2 Movies S1 to S3 [file research.1141.f1.zip › S5.pdf]

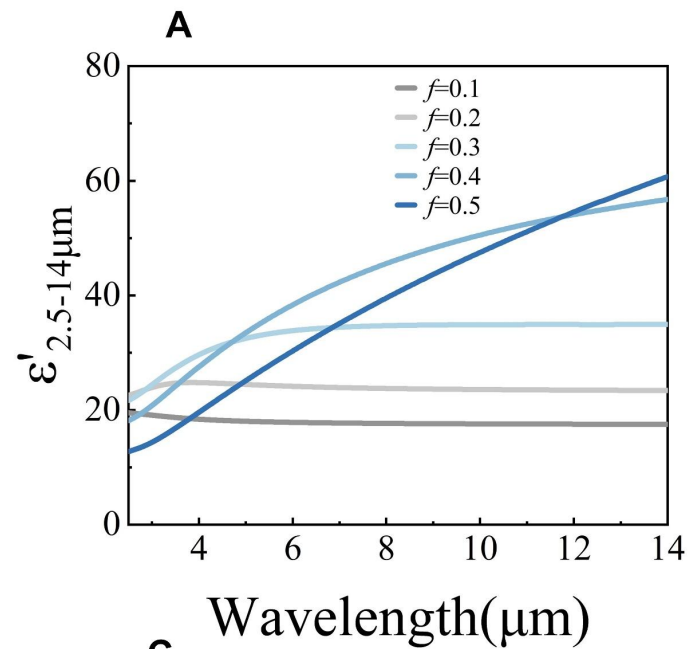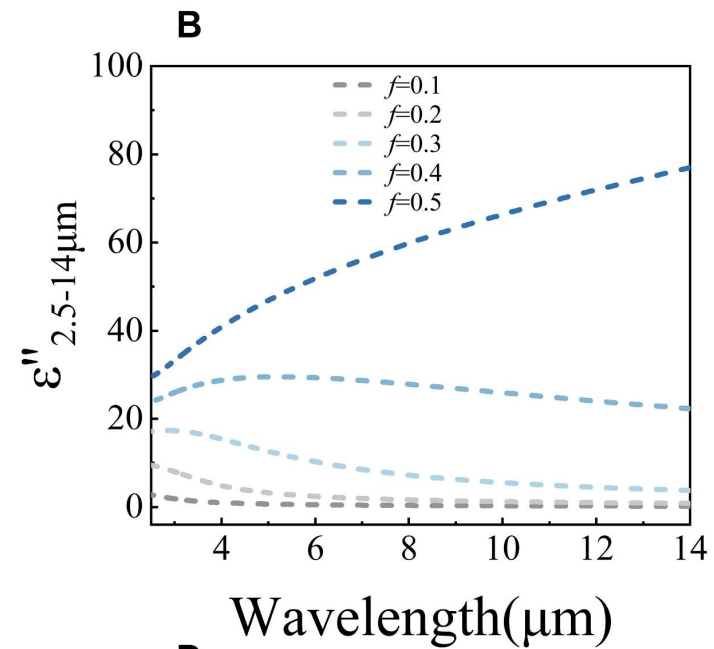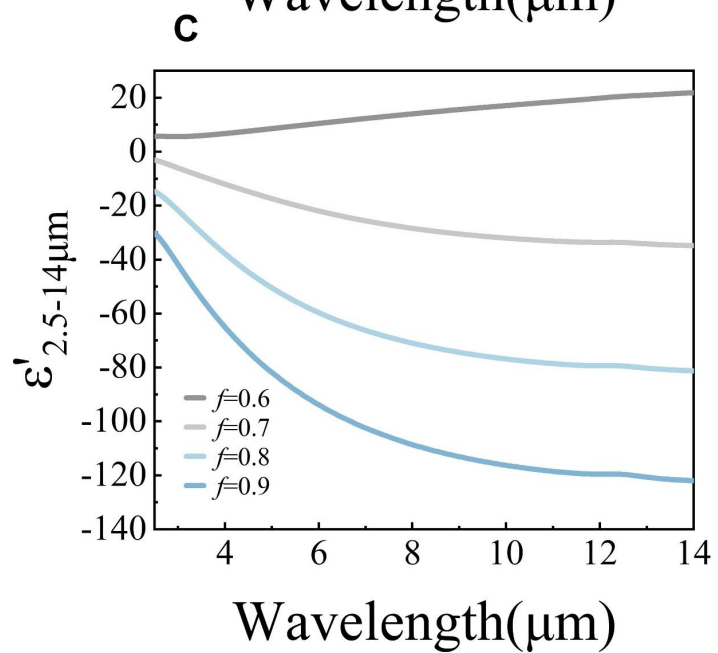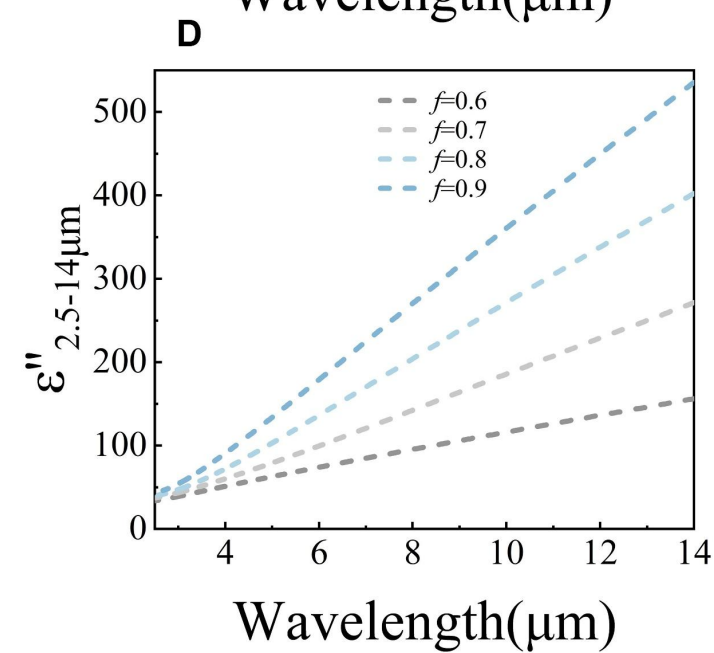

Supplement: Supplementary 1 — Texts S1 to S12 Figs. S1 to S45 Tables S1 and S2 Movies S1 to S3 [file research.1141.f1.zip › S6.pdf]

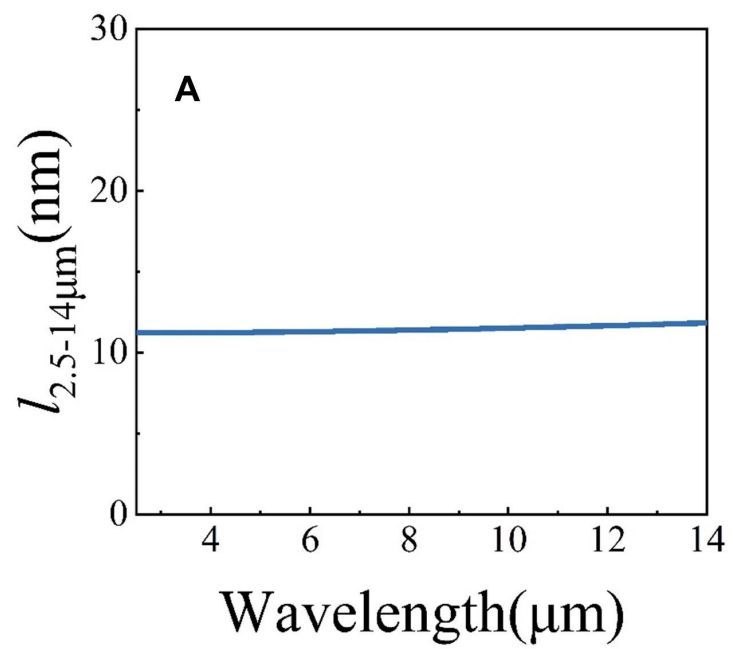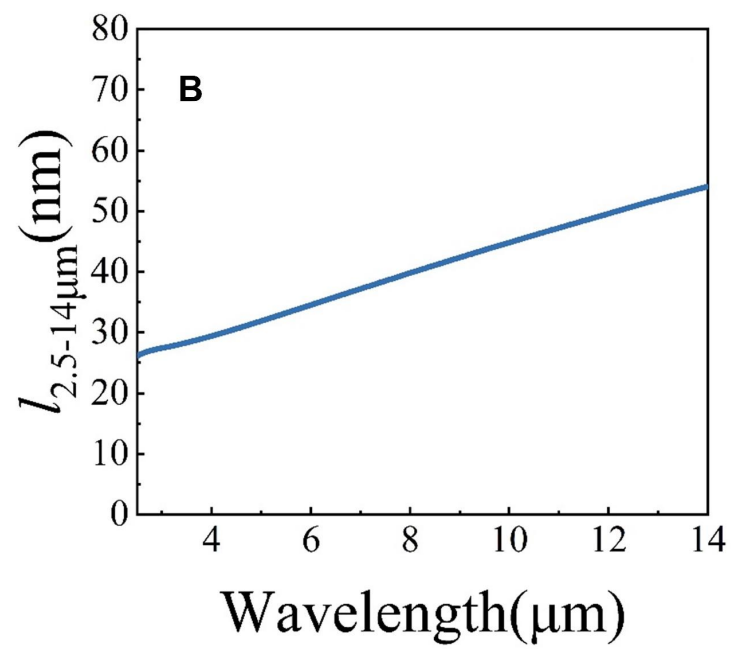

Supplement: Supplementary 1 — Texts S1 to S12 Figs. S1 to S45 Tables S1 and S2 Movies S1 to S3 [file research.1141.f1.zip › S7.pdf]

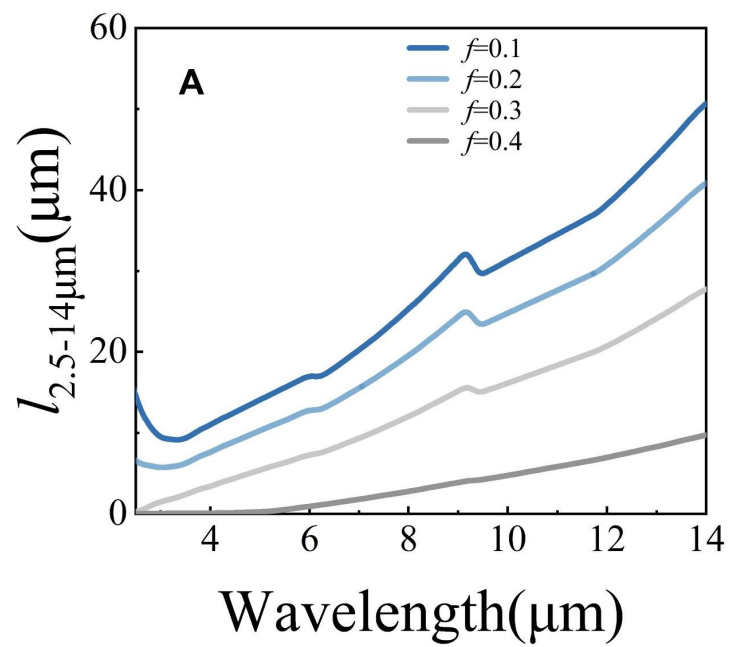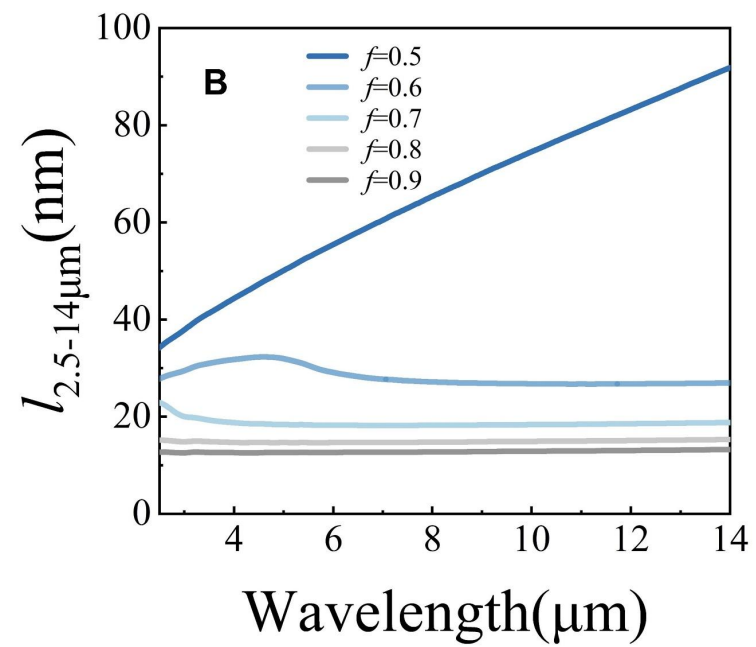

Supplement: Supplementary 1 — Texts S1 to S12 Figs. S1 to S45 Tables S1 and S2 Movies S1 to S3 [file research.1141.f1.zip › S8.pdf]

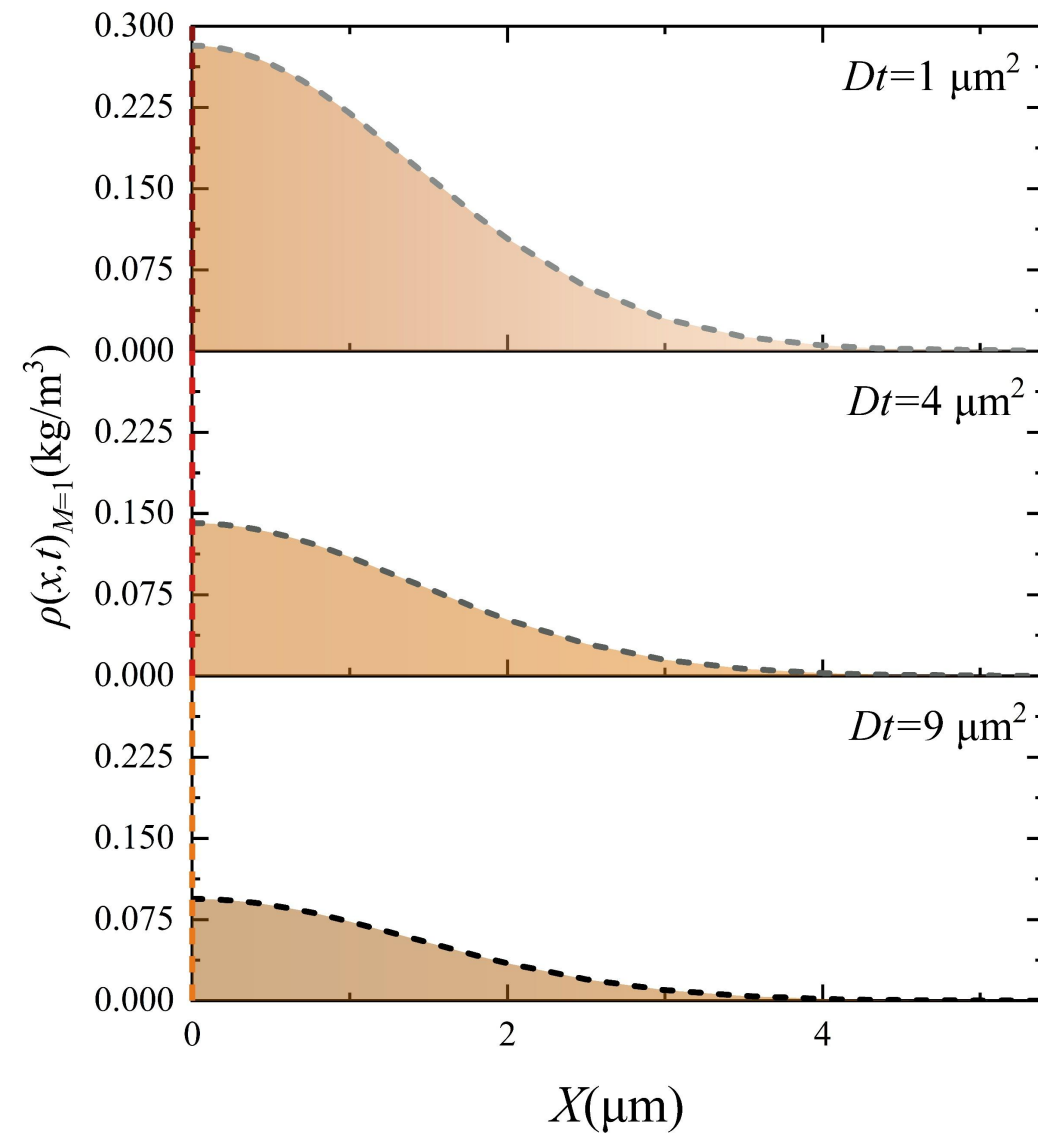

Supplement: Supplementary 1 — Texts S1 to S12 Figs. S1 to S45 Tables S1 and S2 Movies S1 to S3 [file research.1141.f1.zip › S9.pdf]
